# What Predicts Gene Flow During Speciation? The Relative Roles of Time, Space, Morphology and Climate

**DOI:** 10.1111/mec.17580

**Published:** 2024-11-07

**Authors:** Jeffrey W. Streicher, Shea M. Lambert, Fausto R. Méndez de la Cruz, Norberto Martínez‐Méndez, Uri Omar García‐Vázquez, Adrián Nieto Montes de Oca, John J. Wiens

**Affiliations:** ^1^ Natural History Museum London UK; ^2^ Department of Ecology and Evolutionary Biology University of Arizona Tucson Arizona USA; ^3^ Laboratorio de Herpetología, Instituto de Biología Universidad Nacional Autónoma de México Mexico City Mexico; ^4^ Laboratorio de Bioconservación y Manejo, Departamento de Zoología Escuela Nacional de Ciencias Biológicas del Instituto Politécnico Nacional Mexico City Mexico; ^5^ Unidad Multidisciplinaria de Investigación, Facultad de Estudios Superiores Zaragoza Universidad Nacional Autónoma de México Mexico City Mexico; ^6^ Departamento de Biología Evolutiva, Facultad de Ciencias Universidad Nacional Autónoma de México Mexico City Mexico

**Keywords:** climate, gene flow, lizards, morphology, phylogeny, speciation

## Abstract

The processes that restrict gene flow between populations are fundamental to speciation. Here, we develop a simple framework for studying whether divergence in morphology, climatic niche, time and space contribute to reduced gene flow among populations and species. We apply this framework to a model system involving a clade of spiny lizards (*Sceloporus*) occurring mostly in northeastern Mexico, which show striking variation in morphology and habitat among closely related species and populations. We developed a new time‐calibrated phylogeny for the group using RADseq data from 152 individuals. This phylogeny identified 12 putative species‐level clades, including at least two undescribed species. We then estimated levels of gene flow among 21 geographically adjacent pairs of species and populations. We also estimated divergence in morphological and climatic niche variables among these same pairs, along with divergence times and geographic distances. Using Bayesian generalised linear models, we found that gene flow between pairs of lineages is negatively related to divergence time and morphological divergence among them (which are uncorrelated), and not to geographic distance or climatic divergence. The framework used here can be applied to study speciation in many other organisms having genomic data but lacking direct data on reproductive isolation. We also found several other intriguing patterns in this system, including the parallel evolution of a strikingly similar montane blue–red morph from more dull‐coloured desert ancestors within two different, nonsister species.

## 1 Introduction

The processes by which populations become reproductively isolated from each other and thus cease or reduce their gene flow are fundamental to speciation (Coyne and Orr 2004; Futuyma 2005). Various factors may help predict levels of gene flow between a given pair of populations or species. These factors include their similarity in ecology (e.g., habitat) and morphology (e.g., body size), the geographic distance between them (i.e., isolation by distance) and how long they have been separated. Yet few studies (if any) have considered all four factors simultaneously to predict levels of gene flow among lineages. Here, we develop a simple framework for studying the contribution of these factors to gene flow and then apply it to a model system involving lizards in Mexico.

There is considerable precedent for considering these four factors (ecology, morphology, distance and time) to be potentially important for gene flow, reproductive isolation and speciation. But there are also counterexamples for each one. For example, the importance of ecological divergence to speciation is well established (Nosil 2012; Schluter 2009) and found across the tree of life (Hernández‐Hernández et al. 2021). Relevant ecological divergence can occur over small spatial scales (e.g., different host plants) or large spatial scales (e.g., different climates and elevations; Ju et al. 2023; Streicher et al. 2014). However, there are also many known cases of allopatric sister species with limited ecological divergence (Anderson and Weir 2022). Morphological divergence can also strongly impact reproductive isolation and gene flow, through factors such as mechanical isolation based on body size (e.g., Richmond and Jockusch 2007; Richmond, Jockusch, and Latimer 2011) and mate choice based on colour (e.g., Boughman 2001). Yet, there is evidence for morphologically cryptic species in many groups, at least in animals (Li and Wiens 2023; Pfenninger and Schwenk 2007). Geographic distance is a well‐established factor influencing gene flow among conspecific populations, but distinct species can also occur in sympatry with limited gene flow. Finally, divergence time may be particularly important, independently of morphological and ecological divergence (e.g., Singhal and Moritz 2013). For example, incipient species may evolve intrinsic genetic barriers to gene flow if given enough time, even if they occur in very similar environments (e.g., mutation order speciation; Schluter 2009). But in some groups, gene flow may remain possible for tens of millions of years after lineage splitting (Jancúchova‐Lasková, Landova, and Frynta 2015). In summary, there is evidence that each of these four factors can be important for predicting gene flow but also ample precedent that they might not be in particular cases. The relative importance of these factors is therefore not obvious in advance.

Our framework for studying the importance of these four factors for gene flow involves four steps. First, we use genomic data to estimate evolutionary relationships within a clade of closely related species. This clade has considerable variation in levels of morphological and ecological divergence among species and populations. Second, we use these genomic data to estimate levels of gene flow among many geographically proximate pairs of lineages, ranging from pairs that are clearly conspecific to those that clearly are not. Third, we estimate levels of divergence in morphology and ecology among these pairs of populations, along with their divergence times and geographic distances. Fourth, we use general linear models to determine which of these four factors (alone or in combination) best predicts levels of gene flow among conspecific and heterospecific populations.

Many studies have applied a broadly similar approach to analyse speciation. For example, many studies have examined the correlates of reproductive isolation between species pairs, using divergence in genes, ecology, morphology and/or behaviour as predictors of isolation (e.g., Coyne and Orr 1989; Christie and Strauss 2018; Funk, Nosil, and Etges 2006; Martin and Mendelson 2016; review in Matute and Cooper 2021). These studies typically used relatively direct measures of reproductive isolation (e.g., mating success and hybrid offspring produced) but relatively indirect measures of gene flow. Here, we use population genomic data to estimate gene flow in a clade where more traditional measures of reproductive isolation would be relatively difficult to apply. The general approach used here may be applicable to many other nonmodel systems. In a similar approach, Singhal et al. (2021) examined the impacts of divergence time, geographic distance and past climate change velocity (last 21,000 years) on introgression between many species pairs of birds using phylogenomic data. However, they did not examine morphological or climatic divergence between species.

**FIGURE 1:**
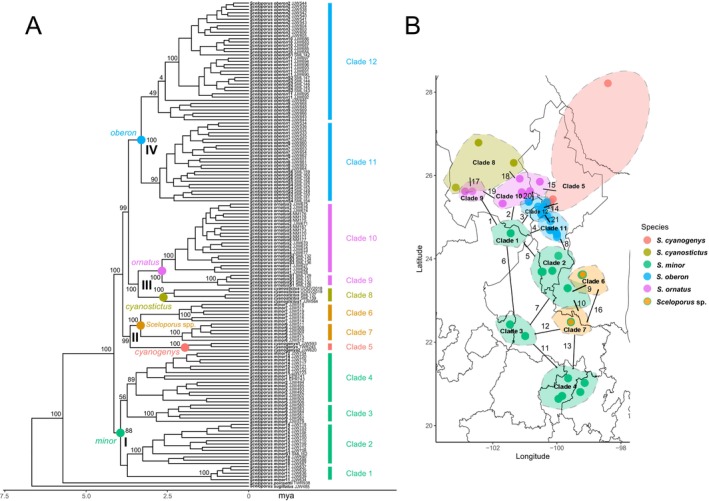
(A) Time‐calibrated (RelTime) phylogeny for select species of *Sceloporus* based on a concatenated maximum‐likelihood analysis of 116,773 SNPs. Bootstrap support is shown on branches among 12 clades (numbered on the right). *Sceloporus jarrovii* and *S. torquatus* were used as outgroups but do not appear because they were removed by RelTime. The complete maximum‐likelihood phylogeny is available for download on the NHM Data Portal (Streicher et al. 2024, https://doi.org/10.5519/5p2mvay1). Given space constraints, support values are not shown for nodes within these 12 clades. Roman numerals indicate four deeper clades that were subsampled for species delimitation analyses. Details on each individual sampled are given in Table S1. (B) Map depicting the geographical distribution of the sampled individuals and of the clades inferred in the phylogeny. The 21 comparisons of lineages are indicated by numbers corresponding to Table 1. Note that the colours around clades are only shown to highlight where these clades occur in general and are not polygons showing the precise geographic range of each clade.

Our study system involves a clade of lizard species (spiny lizards, genus *Sceloporus*) in northeastern Mexico (Figure 1). *Sceloporus* is among the most species‐rich clades of lizards in North America, with most of the 117 currently described species occurring in the United States and Mexico (Uetz et al. 2024). Many of these species are known to hybridise (Arévalo et al. 1993; Wiens et al. 1999; Leaché and Cole 2007; Lambert et al. 2019; Pavón‐Vázquez et al. 2024). A particularly interesting clade of ~6 species belongs to the *S. poinsettii* species group (*sensu* Wiens et al. 2010). These include *S. cyanostictus*, *S. cyanogenys*, *S. minor*, *S. oberon* and *S. ornatus*. The recently described *S. gadsdeni* (Díaz‐Cárdenas et al. 2017) also belongs to this clade.

**FIGURE 2:**
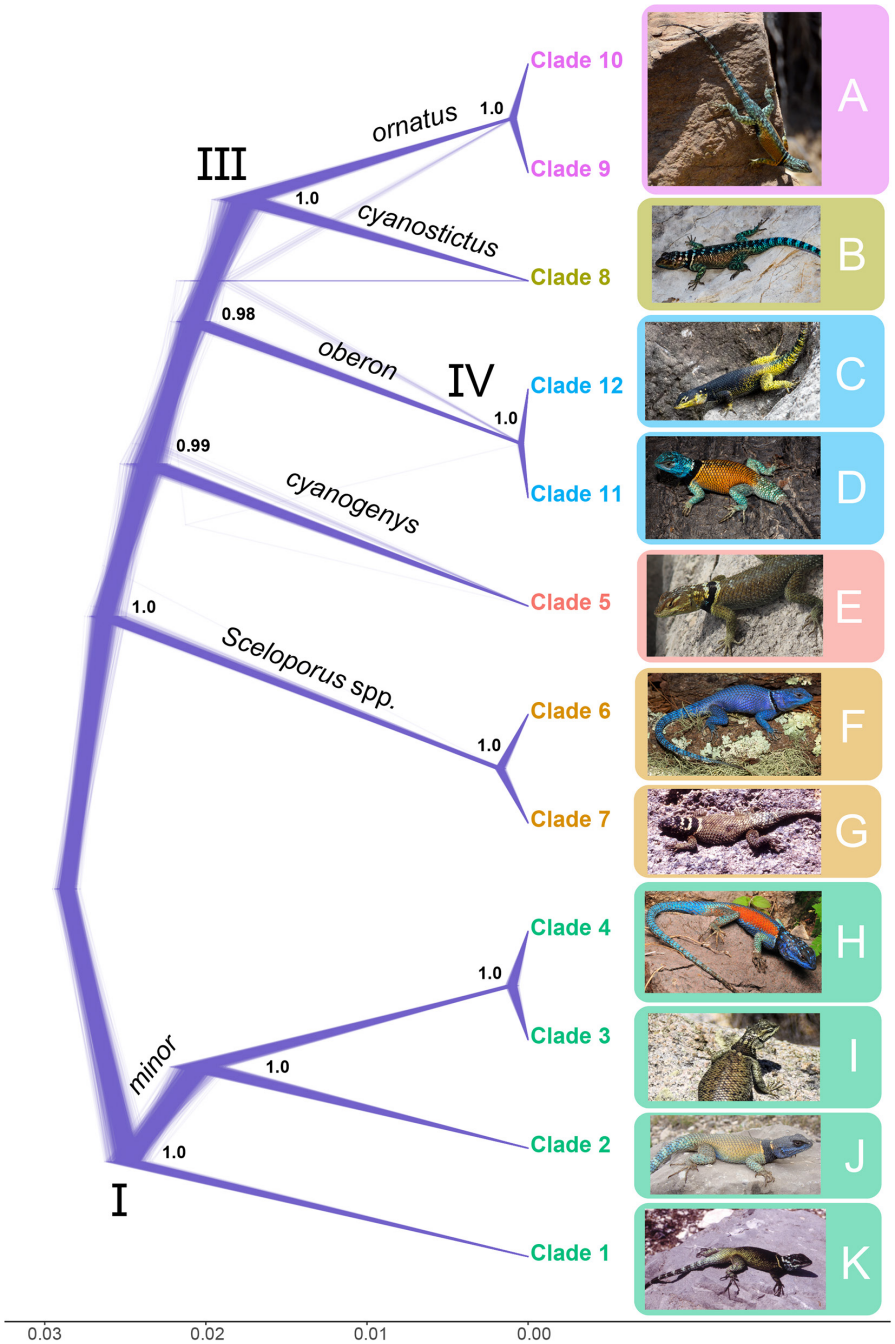
Inferred tree from the multispecies coalescent analysis (SNAPPER). A total of 58,551 biallelic SNPs were analysed, using as tip taxa the two most complete individuals (least missing data) from each of the 12 clades identified by the concatenated analysis (Figure 1). Post burn‐in trees are depicted using a DensiTree function from ggtree in R (Yu et al. 2016). No outgroup taxa were included (see Appendix S4), and trees were rooted automatically by SNAPPER, which infers species trees directly by estimating the probability of allele frequency change across ancestor to descendent branches (Stoltz et al. 2020). Roman numerals correspond to the three deep clades that were also recovered by the concatenated analysis (Figure 1). Support values are posterior probabilities, and the scale bar is in expected mutations per site. Images to the right are of representative individuals of the focal species. From top to bottom: (A) *S. ornatus* from García in Nuevo León, photograph by Daniel Montoya; (B) *S. cyanostictus* from Mina in Nuevo León, photograph by Daniel Montoya; (C) *S. oberon* from Coahuila, photograph by Eric Centenero; (D) *S. oberon* from Coahuila, photograph by Daniel Arteaga; (E) *S. cyanogenys* from Travis County, Texas, photograph by Elijah Wostl; (F) *Sceloporus* sp. from Jaumave in Tamaulipas, photograph by Sergio Terán Juárez; (G) *Sceloporus* sp. from San Luis Potosí, photograph by John J. Wiens; (H) *S. minor* from Meztitlan in Hidalgo, photograph by Leonardo Badillo; (I) *S. minor* from Salinas in San Luis Potosí, photograph by Luis Stevens; (J) *S. minor* from Galeana in Nuevo León, photograph by Daniel Montoya; and (K) *S. minor* from Concepcion del Oro in Zacatecas, photograph by Shea Lambert. All individuals are presumed to be adult males, except for E, I and K.

This clade is interesting for several reasons. First, the group is morphologically diverse (Figure 2), with striking variation in adult male colouration within and among species (Wiens et al. 1999). For example, males in some populations of *S. minor* have bright blue dorsal colouration (often with red patches; Figure 2F,H), whereas in others, males are dull brown or grey (similar to females and juveniles; Figure 2G). This bright blue colouration appears to have evolved twice within a single species, each time in a different montane region (Wiens et al. 1999). Another striking example is *S. oberon*, in which males in southern populations typically have bright blue or green heads, limbs and tails with bright red or yellow bodies (Figure 2D), whereas the nearby northern populations are predominantly black (Figure 2C). Second, the species collectively occur across a broad range of habitats, from lowland Chihuahuan desert scrub, to montane oak woodland to high‐elevation pine‐fir forest. Third, hybridisation is known to occur among some species pairs, including those with very different morphologies and ecologies (e.g., montane black northern *S. oberon* and brightly coloured lowland desert‐dwelling *S. ornatus*; Lambert et al. 2019). Fourth, many of the species in this group are parapatrically distributed with respect to each other (Figure 1).

In this study, we first used genome‐wide SNP data to infer a phylogeny for this clade. Using this phylogeny, we next identified 21 pairs of lineages and estimated levels of gene flow between each pair. We then tested for correlations between gene flow and multiple factors characterising each pair, including their divergence time, geographical distance, morphological similarity and climatic niche similarity. Our results allowed us to parse the relative roles of multiple factors in predicting gene flow across lineages.

## 2 Materials and Methods

### 2.1 Genome‐Wide SNP Processing and Phylogenetic Inference

We used double‐digest restriction‐site–associated DNA sequencing (ddRADseq; Peterson et al. 2012). These data were generated for a previous study on *Sceloporus* (Lambert et al. 2019; NCBI BioProject: PRJNA504030), but are mostly unpublished. Here, we used RADseq data from 154 individuals (Table S1). Ninety‐four of these individuals were analysed for the first time here, as only 60 were included by Lambert et al. (2019). In total, our sampling included three individuals of *S. cyanogenys*, five individuals of *S. cyanostictus*, 52 individuals of *S. minor*, 62 individuals of *S. oberon* and 28 individuals of *S. ornatus*. We also included four outgroup species: *S. jarrovii*, *S. poinsettii*, *S. sugillatus* and *S. torquatus*. We were unable to include the recently described *S. gadsdeni*, given that we lacked samples for this species. Population identification numbers for *S. cyanostictus*, *S. minor* and *S. oberon* follow Lambert et al. (2019). Note that *S. serrifer* also belongs to this group (Wiens et al. 2010; Wiens, Kozak, and Silva 2013; Lambert et al. 2019) but the three sampled individuals belonging to this species yielded very little data.

We used dDocent 2.9.4 (Puritz, Hollenbeck, and Gold 2014) to assemble ddRADseq data and identify SNPs. We used the automated pipeline of dDocent with the following settings: we quality‐trimmed reads, conducted a single‐end assembly (from the first of the two reads, to reduce the number of linked SNPs in our dataset), *de novo* assembled reads with CD‐HIT (Li and Godzik 2006), used the default *c*‐value of 0.85, mapped reads with default settings in BWA (Li and Durbin 2009) and called SNPs using FreeBayes (Garrison and Marth 2012). Based on the dDocent‐generated coverage plot, we selected a minimum value of five reads. Using the dDocent‐generated unique sequences plot, we selected a minimum value of 15 individuals. This meant that the resulting vcf file contained only SNPs from RADtags that had a coverage of at least five reads (within an individual) and were identified in at least 10% of all sampled individuals. Although a coverage depth of five reads has been shown to correspond to genotyping errors in simulation, these errors are rare and only occur in ~1% of sites (Bresadola et al. 2020). We chose this 10% threshold based on previous work suggesting that the phylogenetic placement of highly incomplete samples can be accurate despite large levels of missing data (e.g., Crotti et al. 2019; Barrientos et al. 2021; Portik et al. 2023). We then used a Python script (available at: https://github.com/edgardomortiz/vcf2phylip) to generate an alignment file for phylogenetic analyses. The resulting alignment contained only variable sites and was derived from the genotypes called in the dDocent‐produced VCF file. This alignment sometimes included more than one SNP per RAD locus. We retained all SNPs from each RAD locus (instead of using only the first SNP from each) because previous comparisons suggest this approach leads to larger, more complete datasets that retain more signatures of gene flow (Buckingham et al. 2022). Sites that were genotyped as heterozygous were included in the final alignment using IUPAC ambiguity codes.

To estimate the phylogeny among these 155 individuals, we first conducted maximum‐likelihood phylogenetic inference in RAxML 8.2.12 (Stamatakis 2014). We used a single partition, which is standard practice for RADseq data (see Lambert et al. 2019). We applied the standard GTRCAT model used in RAxML (general time‐reversible model using the CAT approximation to the gamma model to incorporate rate heterogeneity among sites) and a standard model of ascertainment‐bias correction to account for the alignment only containing variable sites (Lewis 2001). We included only variable sites following standard practice for RADseq data since the full alignment would be very large and difficult to analyse. We performed a thorough search (using exhaustive subtree‐pruning‐regrafting moves) to find the best‐fitting maximum‐likelihood tree. We followed this with 100 rapid bootstrap replicates to estimate branch support.

We also inferred a species tree using the multispecies coalescent model as implemented with the SNAPPER method in BEAST 2.7.6 (Bouckaert et al. 2014; Stoltz et al. 2020). While SNAPPER is more computationally efficient than similar coalescent methods (e.g., SNAPP; Bryant et al. 2012), it still requires substantial time to process large numbers of site patterns and calculate likelihoods. Based on preliminary tests, we observed that using the full sample of individuals and SNPs from our dataset was not a viable strategy with SNAPPER, as it would have taken months to sample 1 million generations using a high‐performance computing cluster. Given this, we reduced the number of individuals to make the analysis feasible. In total, we performed five analyses to explore the influence of (1) number of individuals used per putative species and (2) outgroup sampling (see Section 3 for additional details). In all SNAPPER analyses, we ran 1 million generations on each sampling strategy and then checked convergence statistics and topological support using the R package *babette* (Bilderbeek and Etienne 2018). We utilised R version 4.2.1 (R Core Team 2023).

### 2.2 Clade Identification and Species Delimitation

We compared gene flow between pairs of lineages that were geographically proximate. To identify lineages for this analysis, we selected 12 groups of individuals that were monophyletic in the tree and geographically separated from other clades (Figure 1). Note that pairs of clades differed greatly in how close they were to each other spatially. We accounted for this by including the geographic distance between them as a variable (see below). We did not include pairs of clades that were separated from each other by another clade (e.g., we did not include pairs consisting of Clades 2 and 4, 1 and 5, 1 and 8 or 9 and 12; Figure 1).

We recognise that some clades could be further subdivided and that geographic separation can be fuzzy in some cases. However, using smaller and smaller clades would have reduced sample sizes of individuals within them. Importantly, these criteria allowed for a mixture of intra‐ and interspecific lineages that represent a spectrum of evolutionary divergence. However, this required knowing what the species limits were. Importantly, our species delimitation analyses did not consistently support further subdivision of these clades into additional species.

To better understand species boundaries, we performed two analyses that are often used to infer species limits with RADseq data (e.g., Erickson et al. 2020; Ivanov et al. 2021; Streicher et al. 2014). First, we performed probability‐based clustering analyses in STRUCTURE 2.3.4 (Hubisz et al. 2009). We used a three‐step filter with the program VCFtools (Danecek et al. 2011) to reduce the raw SNP data to only those variants that were called for at least 50% of individuals and that had a minimum quality score of Q30 and a minor allele count of 3. We used the populations module of Stacks 2.64 (Catchen et al. 2013) to convert filtered vcf files into files formatted for STRUCTURE. We then used Structure_threader (Pina‐Martins et al. 2017) to run each analysis for 100,000 generations with a burn‐in of 10,000 generations.

As a second approach to species delimitation, we performed an ordination procedure via principal component analysis (PCA) to explore the distribution of RADseq‐derived genotypes in multivariate space. To import RADseq data for PCA, we used the R packages *vcfR* (Knaus and Grünwald 2017) and *adegenet* (Jombart 2008). We used the function ‘dudi.pca’ in *adegenet* to conduct PCA. In contrast to our STRUCTURE analyses, we used all available SNP data for PCA. Because missing data can bias PCA inference of genetic structure (Yi and Latch 2021), we tested for correlations between PC scores and levels of missing data using nonparametric correlation tests (Spearman's rho) in R. We calculated levels of missing data using the '–missing‐indv' command in VCFtools. For comparative purposes, we also performed PCA using the datasets from the STRUCTURE analyses, in which only SNPs with up to 50% missing data were included.

Not all sampled individuals were included in a single STRUCTURE or PC analysis. Instead, we separately analysed four relatively deep clades (I–IV; Figure 1) that together comprised all the individuals sampled in the ingroup. We analysed these four clades to maximise the number of SNPs included in each analysis because as lineages diverge the number of RADseq loci they share decreases (Catchen et al. 2013). In STRUCTURE analyses, the number of clusters (*K*) in each deep clade analysis was set to the number of younger clades they contained (inferred from the maximum‐likelihood analysis) to test posterior assignments of individuals to the putative species represented by these younger clades.

Based on these two sets of analyses, we identified each putative species as a set of individuals that: (1) was assigned with high probability (> 90%) to a unique cluster in STRUCTURE, relative to other individuals included in the analysis; and (2) occupied a distinct region of genotypic multivariate space that did not include individuals of other putative species, based on plotting individuals for the three PCs that explained the greatest variance in the RADseq data. If both criteria were met, we considered a set of individuals to be a potentially distinct species. Similar procedures have been used elsewhere to delimit species with RADseq data including studies in corals (Erickson et al. 2020), spiders (Ivanov et al. 2021) and amphibians (Streicher et al. 2014). Nonetheless, we acknowledge that this is a preliminary species delimitation and we use it here primarily to provide context for which lineages are likely intraspecific versus interspecific.

### 2.3 Quantifying Gene Flow Between Pairs

After identifying lineages and species boundaries, we estimated levels of gene flow between geographically proximate pairs of lineages. We did this in two ways. First, we estimated gene flow using all individuals in each clade. Second, we estimated gene flow using only individuals from the two geographically closest populations (sampling sites) from a given pair of lineages. Hereafter we refer to these as the ‘clade‐based’ and ‘population‐based’ approaches, respectively. We used these two approaches because we recognise that the levels of gene flow between the two geographically closest populations might differ substantially from the overall levels of gene flow between two lineages.

To approximate gene flow across pairs, we conducted a series of analyses using STRUCTURE 2.3.4. We used the same quality‐filtering criteria for SNP datasets as in the species delimitation analyses. For each clade‐based comparison, we included all individuals from the two selected clades and ran the analysis assuming two clusters (*K* = 2). Based on how individuals with 100% assignment probabilities were partitioned among the two clusters, we were able to associate clades with clusters. We then estimated introgression levels (mean frequency of admixture) by tallying the assignment probabilities across individuals for both clusters (K1 and K2). Specifically, for a given pair of clades, we determined the mean frequency of assignment to the other clade among the sampled individuals of both clades. For example, for Comparison 3 (Clades 1 and 12), there is one individual in Clade 1 with 2.7% probability assignment to Clade 2 and four others with 0%, whereas in Clade 12, the five individuals have probabilities of assignment to Clade 1 of 7.1%, 5.3%, 5.8%, 4.3% and 3.3%. The average among the 10 individuals is 2.8%. We also generated an alternative estimate of introgression levels by recording the proportion of individuals that had any evidence of admixture (i.e., that had a nonzero probability of assignment to the other clade).

For three comparisons, all individuals had mixed assignments to the two clusters (e.g., Comparisons 14, 18 and 19; Table 1). In these cases, we used *adegenet* to determine the optimal number of clusters (using the ‘find.clusters’ command) and reran STRUCTURE using that value (in all cases this value was *K* = 3). For these three plots, we determined the mean frequency of admixture by using the procedure described above on the clusters that corresponded with the clades in each comparison.

**TABLE 1:** Pairwise differences among 21 populations of *Sceloporus* from northeastern Mexico.

Comparison	*N*	SNPs	Clade admixture (mean freq.)	Clade admixture (prop. ind.)	Population admixture (mean freq.)	Population admixture (prop. ind.)	Morphology (PC)	Climate (PC)	Time	Geographic distance (km)
1. Clade 1 (*S. minor*) vs. Clade 9 (*S. ornatus*)	7	6251	0.00	0.00	0.00	0.00	14.7	79.2	4.14	173.1
2. Clade 1 (*S. minor*) vs. Clade 10 (*S. ornatus*)	24	15,059	0.31	0.08	0.00	0.00	14.7	39.2	4.14	113.6
3. Clade 1 (*S. minor*) vs. Clade 12 (*S. oberon*)	38	15,312	0.75	0.16	2.85	0.60	5.7	20.4	4.14	116.0
4. Clade 1 (*S. minor*) vs. Clade 11 (*S. oberon*)	21	13,204	0.33	0.10	0.10	0.11	8.0	9.2	4.14	135.1
5. Clade 1 (*S. minor*) vs. Clade 2 (*S. minor*)	18	14,580	0.00	0.00	0.00	0.00	1.8	5.7	3.73	168.1
6. Clade 1 (*S. minor*) vs. Clade 3 (*S. minor*)	10	10,012	0.00	0.00	0.00	0.00	2.6	20.0	3.94	259.6
7. Clade 2 (*S. minor*) vs. Clade 3 (*S. minor*)	18	15,587	6.55	0.39	11.21	0.22	2.9	14.3	3.94	191.9
8. Clade 2 (*S. minor*) vs. Clade 11 (*S. oberon*)	29	18,811	1.24	0.21	2.65	0.33	8.2	12.7	4.14	118.4
9. Clade 2 (*S. minor*) vs. Clade 6 (*Sceloporus* sp.)	18	15,065	0.69	0.11	0.00	0.00	10.3	53.6	4.14	96.1
10. Clade 2 (*S. minor*) vs. Clade 7 (*Sceloporus* sp.)	19	15,200	0.59	0.11	0.54	0.11	4.6	89.6	4.14	145.8
11. Clade 3 (*S. minor*) vs. Clade 4 (*S. minor*)	15	15,283	0.23	0.07	0.57	0.06	4.6	42.7	3.73	238.1
12. Clade 3 (*S. minor*) vs. Clade 7 (*Sceloporus* sp.)	11	10,794	0.26	0.09	0.29	0.10	3.6	104.0	4.14	172.1
13. Clade 4 (*S. minor*) vs. Clade 7 (*Sceloporus* sp.)	16	14,912	1.39	0.19	0.71	0.13	3.8	61.7	4.14	182.0
14. Clade 5 (*S. cyanogenys*) vs. Clade 12 (*S. oberon*)	36	14,565	4.76	0.28	7.16	0.80	5.7	40.9	3.87	156.9
15. Clade 5 (*S. cyanogenys*) vs. Clade 10 (*S. ornatus*)	22	14,553	0.66	0.09	2.92	0.20	15.0	93.3	3.87	176.5
16. Clade 6 (*Sceloporus* sp.) vs. Clade 7 (*Sceloporus* sp.)	11	9051	0.73	0.09	0.73	0.09	8.1	39.1	3.32	131.4
17. Clade 8 (*S. cyanostictus*) vs. Clade 9 (*S. ornatus*)	6	5797	0.00	0.00	0.00	0.00	7.6	21.7	3.40	84.2
18. Clade 8 (*S. cyanostictus*) vs. Clade 10 (*S. ornatus*)	23	14,891	0.81	0.13	0.20	0.33	7.6	18.3	3.40	140.2
19. Clade 9 (*S. ornatus*) vs. Clade 10 (*S. ornatus*)	21	12,614	13.33	0.14	87.32	1.00	0	40	2.67	162.3
20. Clade 10 (*S. ornatus*) vs. Clade 12 (*S. oberon*)	52	20,556	1.10	0.25	0.90	0.14	12.4	59.6	3.70	79.2
21. Clade 11 (*S. oberon*) vs. Clade 12 (*S. oberon*)	49	17,318	7.00	0.45	42.63	1.00	4.9	11.7	3.30	61.2

*Note: N* is the combined number of individuals in the two populations being compared. Estimates of admixture are based on clades and populations and either the mean frequency of admixture (mean freq.) or the proportion of individuals showing admixture (prop. ind.). Divergence in morphology and climate is based on multiple PCs. Divergence times (Time, millions of years ago) correspond to the tree depicted in Figure 1. Geographic distances are from clade‐based estimates.

For each population‐based comparison, we used the same methods as for the clade‐based STRUCTURE analyses but included only individuals from the two adjacent populations. The comparisons of closest populations (and which individuals were used) are described in detail in Appendix S1.

To provide additional context for our STRUCTURE‐based gene flow estimates, we used migration edges inferred by TREEMIX 1.13 (Pickrell and Pritchard 2012). We used the SNAPPER dataset (two individuals per ingroup tip) with outgroups included (*S. jarrovii*, *S. poinsettii*, *S. sugillatus* and *S. torquatus*). Prior to analysis, we removed all missing data using VCFTOOLs. To determine the optimal number of migration edges (= admixture events), we used OptM (Fitik 2021) to assess TREEMIX results under the conditions of 1–12 edges using three iterations for each edge setting. We otherwise used default settings (i.e., the Evanno method) in OptM. TREEMIX uses a covariance matrix to identify those populations/tips that are more closely related than suggested by the modelled bifurcating tree. As such, it is unable to infer admixture events as occurring between sister taxa in the tree, and this differs from our STRUCTURE‐based analysis where we estimated gene flow for several pairs of sister taxa (Table 1). Given this difference, we compared TREEMIX results to STRUCTURE‐based results by comparing inferred gene flow among nonsister clades in each analysis.

### 2.4 Temporal Divergence and Geographic Distance Between Pairs

We estimated divergence dates for the concatenated maximum‐likelihood tree using the RelTime method (Tamura et al. 2012) in MEGA 11 (Tamura, Stecher, and Kumar 2021). We used relative rates and a Tamura‐Nei substitution model in a maximum‐likelihood framework to infer the time tree. We used a single calibration with an age of 5.72 million years ago (±0.25 million years [normally distributed confidence interval]) for the split between *S. poinsettii* and our ingroup. This age was based on the Bayesian estimate from a previous large‐scale analysis, which included many species and genera and multiple fossil calibration points (Leaché et al. 2016). We recorded the divergence time between each pair of clades. The age estimates were the same for both clade‐based and population‐based comparisons.

To estimate geographic distances between pairs, we recorded the average coordinates of all individuals we sampled in a clade (clade‐based comparisons) or population (population‐based comparisons). We then used the R package *geosphere* (Hijmans, Williams, and Vennes 2015) to calculate Haversine distances (in km) between the averaged coordinates of pairs. Although this is a standard approach, we recognise that these distances are simplistic, and do not account for elevational variation between locations. However, given the large distances and limited elevational variation, we assume that alternative distance estimates would give similar results. Furthermore, dispersing populations could presumably disperse around the tallest peaks or ridges separating a given pair of locations.

### 2.5 Morphological and Climatic Niche Divergence Between Pairs

We used the morphological dataset from Wiens and Penkrot (2002), which included data from all clades analysed here (if not every population). We excluded two characters that had missing data in some populations. These were the number of subdigital lamellae on the fourth toe of the hindlimb (Character 24) and male dorsal colouration (Character 41). This resulted in a dataset of 42 morphological characters (character definitions in Appendix S2). These consisted of 23 scalation characters, 16 colouration characters and three morphometric characters (body size and limb and head size relative to body size). The data consisted of mean values and frequencies for each population. Note that these characters were selected by Wiens and Penkrot (2002) for their use in systematics (i.e., species delimitation) and were not chosen to necessarily be relevant for speciation nor to be evolving neutrally. Nevertheless, these characters could potentially reflect adaptation to different habitats or microhabitats (e.g., morphometric and scale characters) and sexual selection and mate choice (e.g., sex‐specific colouration characters).

To calculate morphological divergence between pairs of clades, we first performed PCA on the full morphological datasets for clade‐based and population‐based comparisons. We then estimated divergence between pairs of clades and populations using PCA to identify major axes of morphological variation and then weighed PC scores based on the total variance they explained. We retained the PC axes necessary to cumulatively explain 99% of the variance. We acknowledge that there are other strategies for retaining PCs. However, we weighted axes proportionally to the variance that they explained (see equation below). Therefore, our results should not be heavily influenced by including minor axes that might be excluded using other strategies. We found that > 99% of morphological variance was described by nine PCs for the clade‐based comparison and 13 PCs for the population‐based comparison. We used the following equations to calculate PC‐weighted average morphological differences between each pair of clades or populations:

$$\text{Clade divergence in morphology}=\frac{\sum_{k=1}^{9}\left|x-y\right|*z}{9}$$

$$\text{Population divergence in morphology}=\frac{\sum_{k=1}^{13}\left|x-y\right|*z}{13}$$

where *x* is the PC score for Taxon 1 (for a given PC), *y* is the PC score for Taxon 2, *z* is the percentage of variance explained by the PC and *k* is the number of the PC.

The dataset of Wiens and Penkrot (2002) lacked morphological data for several populations sampled here. Therefore, we assigned morphological data from Wiens and Penkrot (2002) to clades and populations using justifications detailed in Appendix S3. In some cases, morphological data collected from a single population of a clade were applied to multiple populations of that clade. For one comparison (*S. ornatus* [Clade 9] vs. *S. ornatus* [Clade 10]), we used a value of 0 for morphological divergence because the comparison was within the same species, and we only had morphological data for one population corresponding to Clade 9 of *S. ornatus*. Because the similarity of these clades was potentially inflated by doing this, we also examined the effects of removing this comparison on the analyses.

We obtained climate data for each locality from the WorldClim 2 dataset (Fick and Hijmans 2017). The dataset consisted of 19 temperature and precipitation variables. We used QGIS 2.18 (QGIS.org, 2018) to extract climatic data from each georeferenced locality. Climatic data were obtained at the smallest spatial resolution (30 arc sec; equivalent to ~1 km^2^). We used these data for both clade‐based and population‐based comparisons. To calculate the level of climatic niche divergence between each pair of clades or populations, we used an approach similar to that used for the morphological dataset. The only difference was that 19 climatic variables were used to calculate divergence and four (clade‐based) or three (population‐based) PCs were used to calculate PC‐weighted divergence (the first three to four PCs explained > 99% of the variance in the climate datasets). Specifically, we calculated PC‐weighted average values to estimate the overall difference in climatic niches. The following equations were used:

$$\text{Clade divergence in climate}=\frac{\sum_{k=1}^{4}\left|x-y\right|*z}{4}$$

$$\text{Population divergence in climate}=\frac{\sum_{k=1}^{3}\left|x-y\right|*z}{3}$$

where variables represent the corresponding elements described above for the morphological comparisons. For clades with multiple geographic coordinates, we averaged the climatic data from these coordinates before conducting PC‐based comparisons.

We note that we focused here on climatic similarity between populations and species, assuming that there may be reduced gene flow between populations occupying habitats that are climatically unsuitable for each other. This follows from a model of ecological speciation (Rundle and Nosil 2005; Schluter 2009; Nosil 2012) and specifically speciation through climatic‐niche divergence (Hua and Wiens 2013). On the other hand, allopatric populations can potentially be isolated by a barrier of climatically unsuitable habitat (e.g., speciation through climatic‐niche conservatism; Wiens 2004; Hua and Wiens 2013). This could be tested by comparing climatic data from each population pair and from the areas separating them (e.g., Kozak and Wiens 2006; Jezkova and Wiens 2018) or potentially using an isolation‐by‐resistance approach (McRae 2006). However, many populations and species analysed here were more‐or‐less parapatric and were not (to our knowledge) separated by areas of climatically unsuitable habitat. Furthermore, we are not fully confident that both taxa were absent between all sampled population pairs, especially given the lack of obvious barriers.

### 2.6 Testing for Associations Between Gene Flow and Other Variables

We analysed the predictors of gene flow (time, geographic distance, morphological divergence and climatic divergence) using Bayesian generalised linear models (GLM) implemented in the R package *MCMCglmm* v.2.29 (Hadfield 2010). We selected this method because our data were not normally distributed, included count data and similar variables (e.g., with many repeated values including zero), and variance that was greater than the mean. This approach is known to be robust to these issues (Browne and Draper 2006; Zhao et al. 2006). We did not use Mantel tests (often used for comparing pairwise distance matrices) because we were not comparing all possible combinations of pairwise distances between clades and populations, but rather only those from selected geographically adjacent clades.

In all cases, we used estimated gene flow as the response (dependent) variable. The predictor (independent) variables included time, space, morphological divergence and climatic divergence. We treated all the predictor variables as fixed and used a default prior in MCMCglmm v.2.29. We did not perform phylogenetically corrected GLMMs because our data points were pairwise comparisons and not species measurements. We think it would be nonsensical and misleading to treat these pairwise comparisons (e.g., morphological differences between species) as a trait that is inherited phylogenetically.

We generated a series of 14 models including each of the single predictor variables and models featuring multiple predictors (see Section 3 for list of models). These models represent all combinations of predictor variables. Each model was run for 1 million iterations with a burn‐in of 1000 and a sampling interval of 200. We then assessed model convergence using standard diagnostic plots and ensured that effective sample sizes exceeded 3000 for variables in all models (Hadfield 2010). We determined the best fit GLMM using model ranking based on deviance‐information criterion (DIC) scores. To calculate DIC scores, we used Gaussian error distributions.

Given that several focal lineages were likely conspecific (see below), we also compared gene flow estimates between and within species to determine the relative levels of intra‐ and interspecific gene flow. This was done by categorising clade and population comparisons based on the results of our species‐delimitation analyses. We performed four comparisons of intra‐ to interspecific gene flow: (1) clade‐based mean admixture; (2) clade‐based proportion of individuals experiencing admixture; (3) population‐based mean admixture; and (4) population‐based proportion of individuals experiencing admixture.

## 3 Results

### 3.1 Phylogenetic Results, Species Delimitation and Clades

The final alignment consisted of 116,773 SNPs (= base pairs). The average and range of missing data per individual were 37.9% and 10.0%–95.5%, respectively. Prior to phylogenetic analysis, we identified two individuals of *S. ornatus* (SML 126 and JAM 652) and one individual of *S. oberon* (JJW 665) that had large amounts of missing data. These were removed from the alignment. After their removal, the range of missing data per individual was 10.0%–89.9% and the mean was 37.3%. We conducted maximum‐likelihood concatenated phylogeny estimation on the remaining 152 individuals. Across the alignment, there were 115,189 distinct patterns of variation and 37.1% missing data cells in total.

The ingroup was well supported as monophyletic (bootstrap support, BS = 100). We identified 12 focal clades (Figure 1). These were generally well supported (BS > 87). The single exception was in *S. oberon*, which consisted of two clades, one of which was well supported (Clade 11, BS = 100) and one which was not (Clade 12, BS = 49). Nevertheless, these two clades were supported as distinct by our species‐delimitation analyses (see below), and largely correspond to the populations of *S. oberon* with predominantly red (Clade 11) versus black (Clade 12) adult male dorsal colouration.

Our phylogenetic analysis (Figure 1) placed two of the clades originally assigned to *S. minor* (Clades 6 and 7) as the sister group of *S. cyanogenys* (Clade 5). The other populations of *S. minor* formed a well‐supported group (Clades 1–4) that was the sister group to all other members of the ingroup (which included *S. cyanogenys*, *S. cyanostictus*, *S. ornatus* and *S. oberon*). Thus, these two sets of *S. minor* populations (Clades 1–4 vs. 6–7) were clearly not conspecific. Hereafter we refer to the group consisting of Clades 6 and 7 as *Sceloporus* sp. (although it might represent two species instead, see below). We refer to these as *Sceloporus* sp. instead of *S. minor* because the type locality of *S. minor* is near Pinos, Zacatecas (Webb and Axtell 1994), where a different clade of *S. minor* populations occurs (Clade 3).

We discuss the results of the five SNAPPER coalescent analyses in detail in Appendix S4, but we provide a brief overview here. The number of taxa analysed ranged from 24 to 37. The number of sites (= SNPs) ranged from 58,058 to 58,551 with 22,704 to 36,342 patterns. A summary of the different sampling strategies can be found in Table S2. For the focal taxa, we used two to three individuals from the 12 clades identified in the concatenated likelihood analysis. Including outgroups (*S. jarrovii*, *S. poinsettii*, *S. sugillatus* and *S. torquatus*) was problematic and led to well‐supported, but demonstrably conflicting trees (see Appendix S4; Figure S1). Therefore, our preferred coalescent tree included only the ingroup taxa (Clades 1–12). This tree also had the highest average posterior probabilities of the five analyses (Figure 2 and Figure S1). This tree was congruent with the concatenated likelihood tree except for the placement of *S. cyanogenys* (Clade 5). This species was placed with *Sceloporus* sp. (Clades 6 and 7) in the concatenated analysis (Figure 1) and with *S. oberon*, *S. ornatus* and *S. cyanostictus* in the SNAPPER analysis.

Prior to species delimitation, we maximised the number of SNPs available by subsampling our RADseq data into four units that corresponded to four relatively deep clades (Figure 1): (I) *S. minor* (BS = 88, Clades 1–4), (II) *S. cyanogenys* + *S* *celoporus* sp. (BS = 99, Clades 5–7), (III) *S. ornatus* + *S. cyanostictus* (BS = 100, Clades 8–10) and (IV) *S. oberon* (BS = 100, Clades 11–12). Three of these deep clades (I, III and IV) were also recovered in all coalescent analyses (Figure 2 and Figure S1). There was also support for deep Clade II in the coalescent analysis with three individuals per tip (Figure S1E). This subsampling into four clades resulted in the following numbers of individuals and SNPs for the STRUCTURE analyses: *S. minor* (*n* = 33, SNPs = 24,547), *S. cyanogenys* + *S* *celoporus* sp. (*n* = 25, SNPs = 15,849), *S. ornatus* + *S. cyanostictus* (*n* = 14, SNPs = 11,809) and *S. oberon* (*n* = 49, SNPs = 17,318).

**FIGURE 3:**
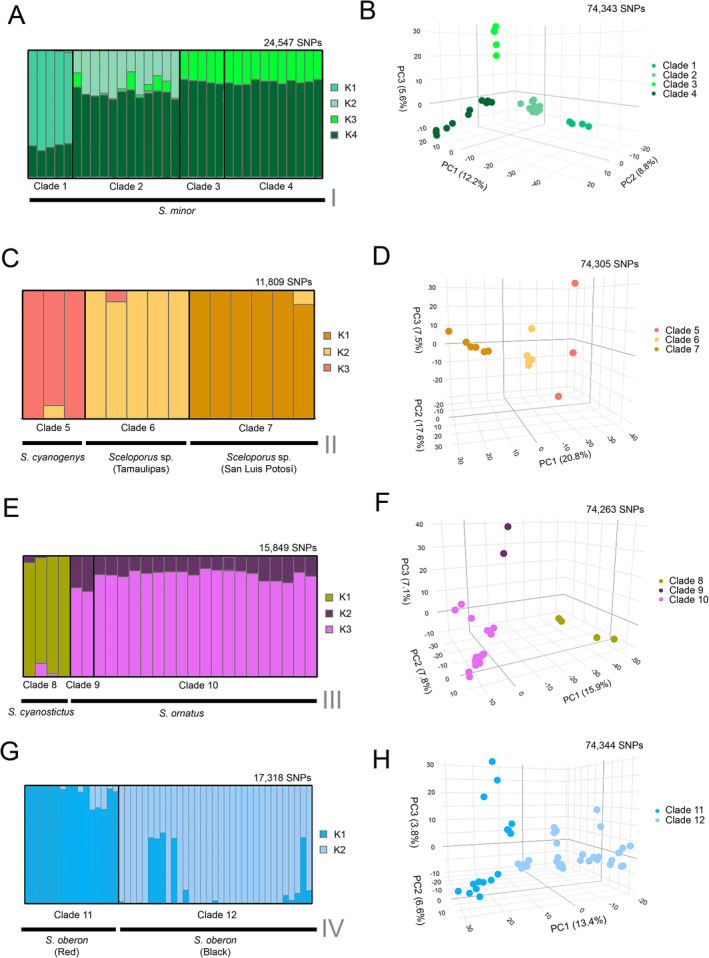
Results of species delimitation analyses applied to four deep clades of *Sceloporus* lizards. STRUCTURE analyses (A, C, E and G) are on the left side and principal component analyses (B, D, F and H) are on the right side. For each analysis, the number of SNPs used is given in the upper right‐hand corner of the graph. Roman numerals correspond to deep clades inferred by the maximum‐likelihood analysis (I–IV, Figure 1). For PCA, the first three PCs are shown. Two‐dimensional plots are shown in Figures S1–S8. The STRUCTURE analyses support only one species in A, three in C, two in E and one or possibly two in G. The number of clusters (*K*) in STRUCTURE analyses is based on the number of younger clades they contain (Clades 1–12, Figure 1). The PCA plots separate three potential species in B, three in D, three in F and two in H. We tentatively followed the more conservative (fewer species) estimates from STRUCTURE.

The results of these analyses are presented in Figure 3A,C,E,G. Based on our species‐delimitation criteria, there was support for eight species in the dataset: (1) *S. minor* (Clades 1–4), (2) *S. cyanogenys* (Clade 5), (3) *S* *celoporus* sp. (Clade 6), (4) *Sceloporus* sp. (Clade 7), (5) *S. cyanostictus* (Clade 8), (6) *S. ornatus* (Clades 9–10), (7) *S. oberon* (Clade 11) and (8) *S. oberon* (Clade 12). There was some evidence of hybridization between Clade 11 (*S. oberon* ‘red’) and Clade 12 (*S. oberon* ‘black’) as also reported by previous authors (Wiens et al. 1999; Lambert et al. 2019). The STRUCTURE analyses did not clearly subdivide clades belonging to *S. minor* (Clades 1–4; Figure 3A) or *S. ornatus* (Clades 9–10; Figure 3E).

We performed PCA using the same four datasets as in the STRUCTURE analyses. However, because we allowed more missing data in the PCAs, this increased the number of SNPs available for each analysis. The total number of SNPs analysed for the PCAs was 74,343 (*S. minor*), 74,305 (*S. cyanogenys* + *Sceloporus* sp.), 74,263 (*S. cyanostictus* + *S. ornatus*) and 74,344 (*S. oberon*). The results of the PCAs are presented in Figure 3B,D,F,H. These plots were visualised using the first three PCs, and we also provide these as two‐dimensional plots in Figures S2–S5. The amount of variance explained across the datasets ranged between 20.8% and 12.2% for PC1, 17.6% and 6.6% for PC2 and 7.5% and 3.8% for PC3. All other axes each explained less than 7% of the overall variance. The percentage of variance explained by each of the first four axes is given in Table S3. In our comparisons of the amounts of missing data to PC scores, we found four significant correlations (Table S4). These were in the *S. cyanostictus* + *S. ornatus* and *S. minor* datasets. Despite these significant correlations, comparisons between the missing‐data permissive strategies and the criteria used in the STRUCTURE analyses (i.e., no more than 50% missing data per individual) revealed highly similar PCA‐inferred clustering patterns (Figures S6–S9), including when the amount of missing data was correlated with some PC scores. Thus, we interpreted this as evidence that the missing data threshold did not greatly influence the clustering patterns in our PCAs.

When viewed in three dimensions, the PCA plots provided some support for all the clades being recognised as distinct species, with clear separation among clades within *S. minor* and *S. ornatus* (Figure 3B,F). However, limiting our comparisons to PC1 and PC2 (Figures S2–S5), clear support is provided for the recognition of nine species: (1) *S. minor* (Clade 1), (2) *S. minor* (Clade 2), (3) *S. minor* (Clades 3–4), (4) *S. cyanogenys* (Clade 5), (5) *Sceloporus* sp. (Clade 6), (6) *Sceloporus* sp. (Clade 7), (7) *S. cyanostictus* (Clade 8), (8) *S. ornatus* (Clades 9–10) and (9) *S. oberon* (Clades 11–12).

Using agreement between the STRUCTURE analyses and PCA, we recognised seven species: (1) *S. minor* (Clades 1–4), (2) *S. cyanogenys* (Clade 5), (3) *Sceloporus* sp. (Clade 6), (4) *Sceloporus* sp. (Clade 7), (5) *S. cyanostictus* (Clade 8), (6) *S. ornatus* (Clades 9–10) and (7) *S. oberon* (Clades 11–12). Hereafter, these seven species are used to define intra‐ and interspecific comparisons for interpreting our results. Importantly, for species represented by multiple clades (*S. minor*, *S. oberon* and *S. ornatus*), all SNAPPER analyses robustly supported their monophyly (Figure 2 and Figure S1), providing further support for this species‐delimitation scheme.

### 3.2 Geographically Proximate Pairs of Lineages, Time and Space

Using the 12 clades, we identified 21 comparisons between geographically adjacent pairs (Table 1; Figure 1). Based on the results of the species‐delimitation analyses, some of these comparisons are clearly between different species (e.g., Comparison 1) whereas others are between conspecifics (e.g., Comparison 19). Pairwise estimates of divergence times calculated between the different lineages ranged from 2.67 to 4.14 million years ago (Figure 1; Table 1). Geographic distances between clade‐based and population‐based estimates were highly correlated (*ρ* = 0.83, *p* < 0.0001; Figure S10). We used clade‐based estimates of geographic distance in our predictive tests of gene flow. Clade‐based geographic distances between the different lineages ranged from 34.7 to 276.9 km (Table 1).

### 3.3 Gene Flow Estimates Between Clades

Genomic datasets for gene flow estimation ranged in size from 5797 to 20,556 SNPs (Table 1). Mean frequency of admixture values ranged from 0 to 13.33 in clade‐based comparisons and 0 to 87.32 in population‐based companions. The proportion of individuals with evidence of admixture ranged from 0 to 0.45 in clade‐based comparisons and 0 to 1.0 in population‐based comparisons (Table 1). Plots of each STRUCTURE analysis used to estimate gene flow are available in Figures S11–S31.

Mean frequency of admixture and proportion of individuals with evidence of admixture were significantly correlated in both clade‐based comparisons (*ρ* = 0.93, *p* < 0.001; Table 2) and population‐based comparisons (*ρ* = 0.89, *p* < 0.001). We also found that the clade‐based and population‐based metrics were significantly correlated for mean frequency of admixture (*ρ* = 0.84, *p* < 0.001) and proportion of individuals with evidence of admixture (*ρ* = 0.83, *p* < 0.001).

**TABLE 2:** Results of Bayesian linear models testing the correlates of gene flow among 21 pairs of populations (clade based).

Model	Intercept (95% CI)	ESS	pMCMC
Gene flow~Time	−5.282 (−8.41, −2.10)	4995	0.0016
Gene flow~Morphology	−0.352 (−0.70, −0.01)	4995	0.0464
Gene flow~Climate	−0.020 (−0.07, 0.03)	4995	0.4328
Gene flow~Space	−0.004 (−0.03, 0.03)	4995	0.7920

*Note:* Mean admixture frequency (gene flow) is the response variable and the fixed predictor variables are divergence times (Time), geographic distance (Space), morphological similarity (Morphology) and climatic‐niche similarity (Climate). Effective sample sizes (ESS) of the posterior of the intercept are indicated along with the 95% confidence intervals and significance scores (pMCMC).

**FIGURE 4:**
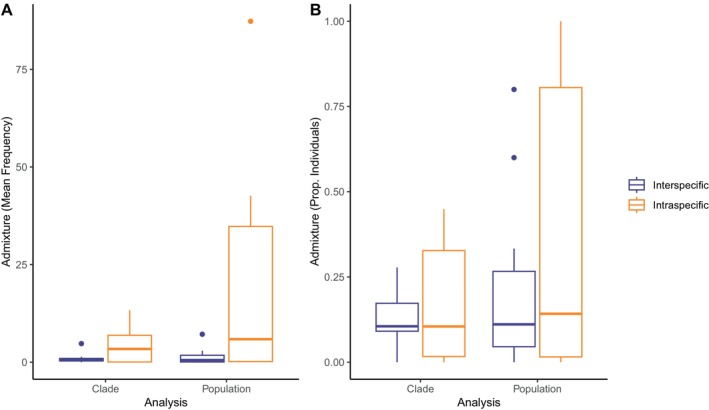
Comparisons of intraspecific (*n* = 6) and interspecific (*n* = 15) gene flow estimates for 21 comparisons among seven putative species of *Sceloporus* included in this study. Box plots are depicted in which dark lines indicate the median among comparisons and boxes indicate the interquartile range. Estimates are from mean admixture frequency (A) and proportion of individuals experiencing admixture (B), for clade‐ and population‐based analyses.

The mean frequency of admixture was generally higher for intraspecific comparisons when compared to interspecific comparisons in both clade‐based and population‐based comparisons (Figure 4A,B). By contrast, there was much less of a difference between intra‐ and interspecific comparisons for the proportion of individuals with evidence of admixture, for both the clade‐based or population‐based comparisons (Figure 4C,D). Given that we expect gene flow to be more common within species than between species, it is possible that the latter metric (proportion of individuals with evidence of admixture) is not as effective as a gene‐flow measure. As such, we only used mean frequency of admixture in our Bayesian GLM tests.

Following the removal of all missing data, the TREEMIX input data matrix contained 4783 SNPs. Using the OptM method, we found that the optimal number of migration edges was 7 (Figure S32), but recall that gene flow between sister clades cannot be inferred. The edge connections were not identical across the three replicates and migration edge weights, which reflect the estimated fraction of introgressed alleles, ranged from 0.02 to 0.59 (Figure S33). Four of the edges connected the ingroup to the outgroup. Among the ingroup edges, direct connections between clades were only inferred for three pairs: (1) Clade 1 to Clade 11 (weight = 0.42), (2) Clade 3 to Clade 4 (weight = 0.43) and (3) Clade 5 to Clade 8 (weight = 0.39). The first two correspond to Comparisons 4 and 11 (Table 1), whereas the third is between nonadjacent species. Overall, the results of STRUCTURE and TREEMIX are similar in suggesting that gene flow is limited between most pairs of species. STRUCTURE shows admixture frequencies < 1.5% between almost all heterospecific pairs (except Comparison 14 at 4.8%; Table 1), whereas TREEMIX also suggests no or negligible gene flow among 13 of the 15 nonsister pairs. On the other hand, the two clades with gene flow inferred by TREEMIX do not necessarily match those with higher admixture frequencies inferred by STRUCTURE.

### 3.4 Morphology and Climate Analyses

**FIGURE 5:**
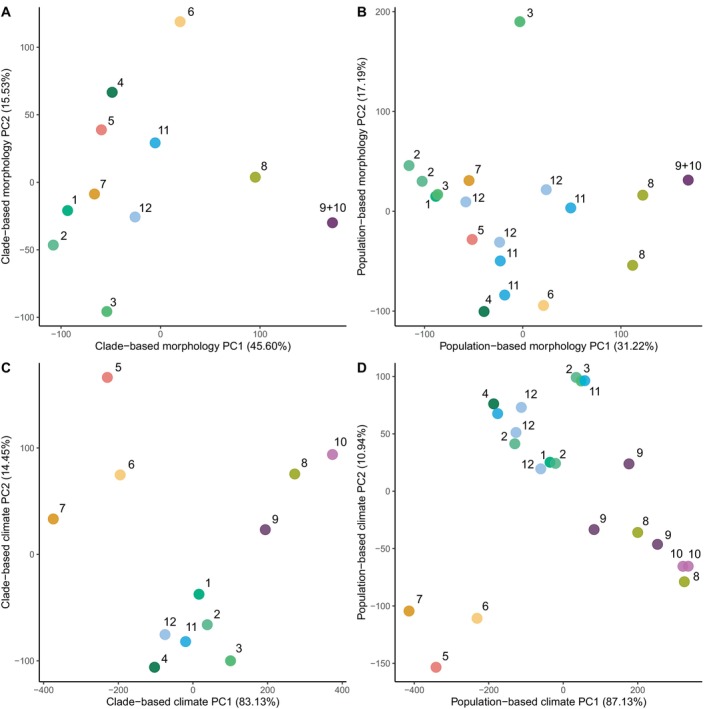
Results of principal component analysis among 12 clades of *Sceloporus* using clade‐based morphological divergence (A), population‐based morphological divergence (B), clade‐based climatic niche divergence (C) and population‐based climatic niche divergence (D). Clades (numbers from Figure 1) are indicated for all data points. Component loadings are given in Tables S5–S8. For *S. ornatus*, we had morphological data only for Clade 9, so Clades 9 and 10 are shown together.

In the clade‐based analysis, morphological PC1 explained 45.4% of the total variance. This PC strongly separated *S. cyanostictus* (Clade 8) and *S. ornatus* (Clades 9–10) from other clades (Figure 5A). PC2 explained 13.9% of the total variance and strongly separated *Sceloporus* sp. from Tamaulipas (Clade 6) from the other species. In the population‐based analysis, PC1 explained 31.2% of the total variance in morphology with a similar separation of *S. cyanostictus* (Clade 8) and *S. ornatus* (Clades 9–10) from other populations. PC2 explained 17.2% of the total variance, but this axis was mostly explained by differences between a single population of Clade 3 (*S. minor*) and the other populations (Figure 5B). The most heavily weighted variables on both PC1 and PC2 were related to colour patterns in both analyses (Tables S5 and S6).

In the clade‐based analysis, climatic PC1 explained 83.1% of the total variance and separated *S. cyanostictus* (Clade 8), *S. ornatus* (Clades 9–10), *S. cyanogenys* (Clade 5) and the two *Sceloporus* spp. (Clades 6–7) from the other species (Figure 5C). PC2 explained 14.4% of the total variance and separated *S. minor* + *S. oberon* (Clades 1–4, 11–12) from the rest of the species. Interestingly, except for two clades of *S. minor*, most *S. oberon* and *S. minor* populations overlapped in multivariate climatic space. The most heavily weighted variable on PC1 was mean annual temperature (Bio1), whereas temperature seasonality (Bio4) was the most heavily weighted variable on PC2 (Table S7). In the population‐based analysis, PC1 explained 87.1% of total variance in climatic niche and PC2 explained 10.9% of the total variance (Figure 5D). Clustering patterns were very similar to the clade‐based analysis, with the first two axes supporting three climatic niche clusters: (1) *S. cyanostictus* + *S. ornatus*, (2) *S. cyanogenys* + the two undescribed *Sceloporus* spp. and (3) *S. minor* + *S. oberon*. Component loadings for the population‐based analysis are in Table S8.

As with the results for geographic distances between pairs, clade‐based and population‐based estimates of divergence were significantly correlated for both morphological (*ρ* = 0.89, *p* < 0.001, Figure S34) and climate (*ρ* = 0.88, *p* < 0.001, Figure S35) data. Given these strong correlations, we focused on the clade‐based results for our predictive tests of gene flow. There were no significant correlations between climatic and morphological divergence in either clade‐based comparisons (*ρ* = 0.22, *p* = 0.33) or populations‐based comparisons (*ρ* = 0.05, *p* = 0.82). We also found no significant correlations between geographic distance and climate divergence (*ρ* = 0.23, *p* = 0.318). However, there was a significant, negative correlation between geographic distance and morphological divergence in clade‐based (*ρ* = −0.47, *p* = 0.033), but not population‐based analyses (*ρ* = −0.20, *p* = 0.38). We found no significant correlations between divergence times and clade‐based climate divergence (*ρ* = 0.29, *p* = 0.205) or morphological divergence (*ρ* = 0.26, *p* = 0.2613).

### 3.5 Bayesian Generalised Linear Models

**FIGURE 6:**
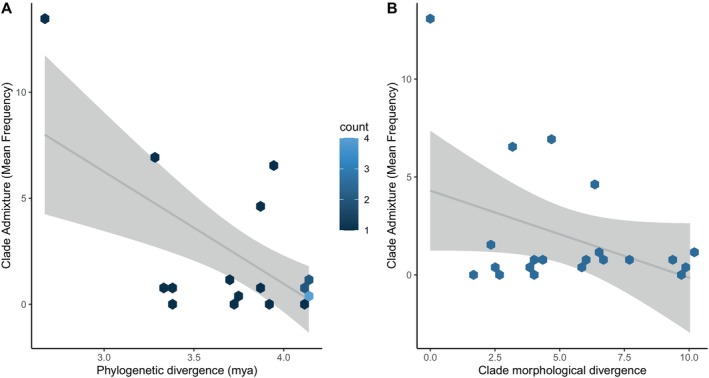
Significant results of Bayesian generalised linear model analysis between mean admixture (clade based) and time (A) and morphology (B). Each data point is 1 of the 21 comparisons of pairs of clades. Grey shading around regression lines corresponds to smoothed conditional means. In the time versus mean admixture plot (A), data point (hexagon) shading corresponds to the number of data points occupying a given hexagon.

Using Bayesian GLMs, we found significant, negative correlations between mean admixture frequency (gene flow) and time (*p* = 0.002, Figure 6A; Table 2). We also found a significant, negative relationship between gene flow and morphological divergence (*p* = 0.046, Figure 6B). This latter relationship remained negative but was not significant if the comparison including the two clades in *S. ornatus* was removed (*p* = 0.306, Figure S36). These two clades were assigned the same value for morphology. As an additional sensitivity test, we removed the four pairwise comparisons (Comparisons 14, 15, 17 and 18; Table 1) involving two species with low sample sizes, *S. cyanogenys* (*n* = 3) and *S. cyanostictus* (*n* = 5). The significant relationships between gene flow and time (*p* = 0.0002, Figure S37A) and gene flow and morphology (*p* = 0.0104, Figure S37B) remained significant when comparisons involving these two species were removed. We did not find significant correlations between gene flow and climatic niche divergence (*p* = 0.4328) or space (*p* = 0.7920). Importantly, time and morphological divergence were not significantly correlated (*ρ* = 0.26, *p* = 0.2613).

We generated 14 models including both single predictor variables and combined predictor variables (Table 3). DIC scores ranged from 104.20 to 116.44. The seven highest‐ranked models all included time as a predictor variable. The best model for predicting gene flow was the model with both time and morphological divergence as predictor variables followed very closely by the model with just time as a predictor variable (Table 3). The difference in support between these two models was negligible.

**TABLE 3:** Results of model comparisons testing the correlates of gene flow.

Rank	Bayesian linear model	R‐structure ESS	DIC score
1	Gene flow~Time + Morphology	4995	104.2022
2	Gene flow~Time	4995	104.2660
3	Gene flow~Time + Space + Morphology	4995	106.2849
4	Gene flow~Time + Space	4995	106.3291
5	Gene flow~Time + Climate	4995	106.3868
6	Gene flow~Morphology + Time + Space + Climate	4995	108.4359
7	Gene flow~Time + Space + Climate	4995	108.5146
8	Gene flow~Morphology	4995	110.4594
9	Gene flow~Morphology + Space	4995	111.6377
10	Gene flow~Morphology + Climate	4995	112.4996
11	Gene flow~Morphology + Climate + Space	4995	113.8157
12	Gene flow~Climate	4995	114.3247
13	Gene flow~Space	4995	114.9962
14	Gene flow~Climate + Space	4995	116.4378

*Note:* Analyses are based on Bayesian generalised linear models (MCMCglmm) used to predict mean admixture frequency (Gene Flow) among 21 pairs of clade‐based populations. Model ranking used the deviance information criteria (DIC). Models are ranked from lowest to highest DIC score, with lower DIC values having better fit. DIC scores < 2 from the best model deserve consideration whereas scores > 3 are far less supported (Spiegelhalter et al. 2002). Predictor variables included divergence times (Time), geographic distances (Space), morphological similarity (Morph) and climatic niche similarity (Climate). Effective sample size (ESS) of the posterior R‐structure is listed.

## 4 Discussion

Understanding the factors that reduce gene flow among populations may be key to understanding speciation. Here, we demonstrate an approach for evaluating the effects of morphological, climatic, temporal and spatial divergence on gene flow among populations using genomic data. Our results from Mexican spiny lizards (*Sceloporus*) showed that divergence time was the best predictor of reduced gene flow among these populations, along with morphological divergence. We found surprisingly little effect of climatic‐niche divergence or geographic distance between pairs. In the sections that follow, we discuss the implications of these results for speciation research, the limitations of our study, some surprising findings related to colour evolution and speciation, and finally the taxonomic implications.

### 4.1 Implications for Speciation Research

Our results in this system suggest that time is the most important factor for explaining reduced gene flow between populations, and potentially for speciation. We found many older pairs with no gene flow and a few younger pairs with more extensive gene flow. One potential explanation for the relationship between time and gene flow is the classic Dobzhanksy–Muller speciation model, in which populations become geographically separated and then evolve intrinsic barriers to gene flow because of epistatic interactions between derived alleles that evolved separately in each population (Coyne and Orr 2004). This model does not require adaptation to different environments, since it can also work when species have different adaptations to the same environment (mutation order speciation; Schluter 2009). Of course, much additional work will be needed to test if this hypothesis is relevant in this system. Nevertheless, there are precedents for the general importance of divergence time for speciation, including in lizards (Singhal and Moritz 2013). We also found that the impact of time on gene flow appears to be independent of other variables, such as climate, morphology and geographic distance. This latter result suggests that time is not simply important as a proxy for divergence in these other variables.

We also found that morphological divergence was a significant predictor of reduced gene flow. This relationship was significant but not strong and was sensitive to inclusion of a single comparison (Clade 9 vs. Clade 10). Additionally, there was gene flow between some population pairs that have strikingly divergent colouration, such as the predominantly black and red populations of *S. oberon* (Clades 11 and 12). In this system, some morphological divergence may be associated with sexual selection on male dorsal colouration, given that much of the variation in colour among populations and species involves sexually dichromatic adult males (Wiens et al. 1999). On the other hand, analyses of populations with strikingly polymorphic male dorsal colouration (e.g., *S. minor*) suggest that there is no female choice on dorsal colouration, nor does it appear to be important in male contest competition (García‐Rosales et al. 2021). Instead, the details of male ventral colouration (i.e., blue belly patches) seem to be more important for female choice. These patches show more limited variation relative to dorsal colouration among these species (Wiens et al. 1999), but the black populations of *S. oberon* (Clade 12) lack blue patches. Yet, these black populations also hybridise with other species and populations with blue patches (e.g., *S. ornatus* and red *S. oberon* populations).

We found that divergence in the climatic niche was not a significant predictor of reduced gene flow. This is surprising because other lines of evidence suggest that climatic niche divergence can be important in lizard speciation, including comparisons of sister species pairs (Jezkova and Wiens 2018) and large‐scale correlations between diversification/speciation rates and rates of climatic niche change among clades (Li and Wiens 2022; Moreira et al. 2024). On the other hand, those studies did not utilise direct measures of gene flow, as we do here. Why might gene flow be uncoupled from niche divergence in this species complex? One potential explanation is that, based on our field experience, most of these species are closely tied to rock outcrops. Combined with behavioural thermoregulation, this may cause them to be exposed to similar thermal environments regardless of large‐scale climate. Furthermore, the spatial isolation of rock outcrops might be more important for limiting gene flow than climatic dissimilarity between habitats. Hypothetically, the spatial scale of the climatic data might explain the lack of a significant effect, but we used relatively fine‐scaled climatic data (1 km^2^), the same scale as in the studies that supported a significant effect of climatic niche divergence on speciation. There might also be other ecological variables besides climate and microhabitat that isolate populations from gene flow (but we are not sure what those would be).

We also found that spatial distances between population pairs did not explain variation in their levels of gene flow. This may be (in part) because most of the pairs compared here are geographically separated and show relatively little gene flow, so this pattern does not change as the distance between populations increases. There might also be a stronger pattern of isolation by distance if we examined populations across the range of each species, rather than focusing on pairwise comparisons as we did here.

We assumed that divergence in morphology and climate would potentially explain limited gene flow. However, we acknowledge that more limited gene flow might also facilitate divergence in morphology and climate (since gene flow may impede morphological and physiological divergence among populations).

Finally, we note that our goal here is to demonstrate an approach for analysing the correlates of gene flow. We do not expect that every study in every group of organisms will have identical results. There may also be many other variables that are relevant to reduced gene flow between populations and incipient species (depending on the species and group), such as divergence in behaviour or microhabitat. There are also various limitations to our study (see next section) that are not limitations of the approach in general. For example, one could use ultraconserved elements (UCEs) or whole‐genome resequencing instead of RADseq data and use different methods for estimating the phylogeny and gene flow.

### 4.2 Limitations

We also note that there are some limitations to the data and methods used in our case study. We address these below. First, all the population pairs compared here are relatively similar in age (2.7–4.1 million years), even those that are ostensibly intraspecific. Different patterns might be found in groups that include much younger divergences. We also note that RADseq datasets that contain only SNPs (i.e., excluding invariant sites) can produce overestimated branch lengths when used for phylogenetic inference, especially in the presence of missing data (Leaché et al. 2015). It is possible that overestimated branch lengths explain the relatively narrow range of divergence times. Yet, despite these potential artefacts and the limited range in inferred divergence times, divergence time was the most important variable for explaining variation in levels of gene flow.

Second, our sampling of species in the group is not complete. Specifically, we did not include *S. gadsdeni* and *S. serrifer*, although both appear to belong to this clade (e.g., Wiens et al. 2010; Wiens, Kozak, and Silva 2013; Leaché et al. 2016; Díaz‐Cárdenas et al. 2017; Lambert et al. 2019). It is also possible that *S. poinsettii* belongs to the ingroup rather than the outgroup (Appendix S4). However, including these species would simply add more species pairs, and should not change results among the many pairs that were already included. Furthermore, our comparisons are not only of sister species. We also note that *S. serrifer* occurs largely outside of the geographic focal area of the study (i.e., northeastern Mexico), whereas *S. poinsettii* is broadly sympatric with many ingroup taxa.

Third, our sampling of populations is also not complete. There are large geographic gaps between the sampling of many clades, such as those between Clade 3 and the other populations (Figure 1). At the same time, we did not find a significant effect of geographic distances between populations on our results. We also note that having more complete geographic sampling may be easier said than done. Our sampling over this huge, mountainous region was limited by where roads are and where these roads intersected with suitable habitats. These species do not occur continuously across landscapes but are generally confined to relatively large rock outcrops. In many areas, these outcrops are very widely spaced (e.g., desert flats). We also note that this region of northern Mexico has become extremely dangerous for fieldwork in recent years (due to drug trafficking and related violence) and some key localities were sampled along the now infamous ‘Highway of Death’ (Mexican Federal Highway 101).

Fourth, we note that different population pairs and species could show different patterns, even within a small group of species. For example, climatic‐niche divergence might be important for one population pair, whereas morphological divergence might be more important for another. Given our small sample sizes, our approach may be insensitive to such variation, and might incorrectly suggest that neither variable is important. We also note that our comparisons include a mixture of sister clades and those that are more distantly related. To make the results more specifically relevant to speciation, it could be restricted to sister taxa only. Alternative measures of gene flow might also be used, and other potentially relevant morphological and ecological variables.

Finally, comparing our approach to gene flow estimation to alternative methods, like TREEMIX (Figure S33), will also be important. In this study, both STRUCTURE and TREEMIX seem to concur that gene flow is relatively limited among these species. It would also be valuable to incorporate methods that can more explicitly distinguish gene flow and incomplete lineage sorting. However, given that our results suggest very limited gene flow among these species, it seems highly unlikely that an alternative method that accounts for incomplete lineage sorting would suggest that levels of gene flow were instead high.

### 4.3 Colour Evolution and Speciation

Species and populations in this group show intriguing variation in male dorsal colouration, with populations ranging from predominantly blue to red, black, yellow and brown–grey. The evolution of these patterns was studied by Wiens et al. (1999), but those authors had only mitochondrial data. Our study provides the opportunity to test those patterns with genomic data.

One of the most intriguing patterns found by those authors was the apparent parallel evolution of blue male dorsal colouration (often with red patches) within *S. minor* (sensu lato). This evolved separately in different montane regions (Tamaulipas in the north and Hidalgo in the south), from among different lowland desert populations in which males tend to have dull brown‐grey dorsal colouration. Our results generally support this pattern but with a surprising twist. Our results suggest that this parallel evolution occurred separately within two different species and that these two species are not closely related (Figure 1). Specifically, we show that *S. minor* is paraphyletic, and that the blue morph evolved both in Clade 4 (Figure 2H) of the restricted *S. minor* and in Clade 6 of *Sceloporus* sp. (Figure 2F; formerly assigned to *S. minor*). The blue morphs in these two species are so similar that they were at one point assigned to the same subspecies (*immucronatus*). In both cases, the blue upland morph seems to have evolved from more dull‐coloured lowland ancestors (for Clade 4 from among Clades 1–3, whereas Clade 6 is sister to lowland Clade 7). Why this blue morph evolved repeatedly, and why it did so in montane populations, remains unclear. The presence of this conspicuous colouration in adult males implies sexual selection, but recent analyses imply that this dorsal colouration is not important in female choice or male contest competition (García‐Rosales et al. 2021).

Another related pattern is that our species‐delimitation analyses here suggest that the southern blue morph (Clade 4) is conspecific with the dull‐coloured lowland morph (Clade 3; Figure 2I). By contrast, the northern blue morph (Clade 6) may be a separate species relative to its closest relative (Clade 7; Figure 2G). However, more population‐level sampling between Clades 6 and 7 (in southern Tamaulipas) would be valuable to confirm this.

We also note that conspicuous sexually dichromatic male colouration has also evolved within other populations, at least in some individuals. These include some montane *S. oberon* (Clade 11, typically with yellow–red body and blue–green head, limbs and tail; Figure 2D), the lowland desert species *S. cyanostictus* (blue and blue–green in some individuals, Clade 8; Figure 2B) and *S. ornatus* (sometimes blue with red patches, Clades 9 and 10; Figure 2A), and in some individuals and populations of other species (e.g., bright blue in some *S. cyanogenys* and bright yellow in some populations of *S. minor*).

### 4.4 Taxonomic Implications

Our results have several implications for the taxonomy of the group. First, as mentioned above, we show that *S. minor* is not monophyletic and consists of two clades that are not closely related. We restrict *S. minor* to Clades 1–4. Based on our trees, Clades 6 and 7, previously assigned to *S. minor* (e.g., Wiens et al. 1999; Wiens and Penkrot 2002), should be recognised as a separate species or possibly two (see above). To our knowledge, there is no available name for this taxon. The mitochondrial analyses of Lambert et al. (2019) show that this species is not conspecific with *S. serrifer* *plioporus*, *S. serrifer* *prezygus* or *S. serrifer*. On the other hand, we find no evidence for the recognition of *S. erythrocyaneus* and *S. immucronatus* as separate species within Clades 1–4 (as suggested by Pérez‐Ramos 2020).

Support for the monophyly of the restricted *S. minor* populations (Clades 1–4) is high in the coalescent analysis (posterior probability = 1.0; Figure 2), but we acknowledge that support is modest in the concatenated analysis (bootstrap = 88%; Figure 1). Nonetheless, their nonmonophyly would suggest that *S. minor* should be recognised as multiple species, not that it is conspecific with another described species (i.e., the clade above *S. minor*, containing the other species in this group, is very strongly supported; Figure 1). Furthermore, our species‐delimitation analyses show only mixed support for recognition of additional species within the restricted *S. minor* (support from PCA but not STRUCTURE; Figure 3).

We suggest that the predominantly red (southern; Clade 11) and black (northern; Clade 12) populations of *S. oberon* may also be distinct species. There are some hybrid individuals between these species, but they otherwise form largely distinct clades and clusters based on RADseq data (Figures 1 and 3) and are very different morphologically. The name *S. oberon* applies to the northern, black populations (Clade 12), but to our knowledge, no name is available for the southern, red populations (Clade 11). Overall, our results suggest that this relatively small clade contains between one and three additional species that should be formally named in the future (Clades 6 and 7 and Clade 11).

Our results also suggest that some taxonomic changes made in this group by Martínez‐Méndez and Méndez de la Cruz (2007) need to be revised. Their analyses were based on mitochondrial data only and included few individuals per species. First, those authors elevated *S. ornatus* *caeruleus* to a distinct species (*S. caeruleus*), and this has been followed by some subsequent authors, including Uetz et al. (2024). Our analyses here (Figure 3) suggest that *S. caeruleus* is not distinct from *S. ornatus*. Specifically, Clade 9, from near the type locality of *S. caeruleus*, does not appear to be distinct from Clade 10. The one individual of *S. ornatus* *caeruleus* included by Martínez‐Méndez and Méndez de la Cruz (2007) was assigned to Clade 9 by Lambert et al. (2019). Second, they considered *S. oberon* to be conspecific with *S. ornatus* based on their placement of one individual of *S. ornatus* within *S. oberon* in their mtDNA trees. But Lambert et al. (2019) showed that this placement was only in mtDNA. Our analyses here further confirm that *S. oberon* and *S. ornatus* are distinct species, with only limited nuclear gene flow between them.

Martínez‐Méndez and Méndez de la Cruz (2007) also synonymised *S. serrifer* *plioporus* with *S. cyanogenys*. However, they had no justification for doing so in their molecular data, since these taxa were reciprocally monophyletic. At the same time, recent molecular analyses suggest that *S. serrifer* *plioporus* is not closely related to *S. serrifer* and *S. prezygus* (Martínez‐Méndez and Méndez de la Cruz 2007; Wiens et al. 2010; Wiens, Kozak, and Silva 2013; Lambert et al. 2019). Thus, it is clear that *S. serrifer plioporus* does not belong in *S. serrifer*, even if it is unclear if it belongs in *S. cyanogenys*. Resolving the relationship with *S. cyanogenys* may be difficult as the population of *S. serrifer plioporus* from its type locality (Encero and Veracruz; Smith 1939) may have gone extinct (N. Martínez‐Méndez, personal observation).

## 5 Conclusions

Here, we demonstrate an approach for analysing the correlates of gene flow among populations and species in a clade of Mexican lizards. We show that divergence time is the most important predictor of gene flow among population pairs in this system, more so than morphology, climatic niche or spatial distance. The general approach used here could be applied to many other organisms. Furthermore, many other types of variables could be included in the same framework (e.g., microhabitat and phenology), depending on the biology of the organisms being studied.

## Author Contributions

J.W.S. and J.J.W. conceptualised the study. J.W.S. conducted analyses. S.M.L., F.R.M.C., N.M.‐M., U.O.G.‐V., A.N.M.O. and J.J.W. conducted fieldwork. S.M.L. conducted laboratory work with assistance from J.W.S. S.M.L. curated raw Illumina data and uploaded them to NCBI. J.W.S. and J.J.W. wrote the initial draft of the manuscript. Additional lizard photographs were obtained by U.O.G.‐V. J.J.W. and S.M.L. acquired funding for the research. All authors contributed to writing and editing the manuscript.

## Conflicts of Interest

The authors declare no conflicts of interest.

## Supporting information


Data S1.

## Data Availability

Raw ddRADseq data are available on the NCBI Sequence Read Archive (Bioproject Accession PRJNA504030). Climate data, morphological data, SNP matrices, phylogenetic trees and R scripts are available on the NHM Data Portal (https://doi.org/10.5519/5p2mvay1). Specifically, the following files are available: (1) R commands for analyses and figure construction, (2) RAxML concatenated alignment, (3) RAxML concatenated tree, (4–11) input data for STRUCTURE and PCA species delimitation analyses, (12) climate data from population comparisons, (13) climate data from clade comparisons, (14) morphology data from population comparisons, (15) morphology data from clade comparisons, (16–17) geographical coordinates of samples and spatial layers for boundaries in Mexico, (18–27) SNAPPER input XML files and resulting tree files and (28–32) SNAPPER log files. The NHM Data Portal is a data repository that is publicly available. Our team of authors represents a collaboration developed among scientists from all countries providing genetic samples (Mexico and United States). Benefits from this collaboration include conservation‐relevant biodiversity discovery. The raw genetic data are publicly accessible as described above.
