# Supplementary material for: What Predicts Gene Flow During Speciation? The Relative Roles of Time, Space, Morphology and Climate
Source: Mol Ecol. 2024 Nov 7;33(23):e17580. doi: 10.1111/mec.17580 (PMC11589662; doi:10.1111/mec.17580)
Supplement: Supplementary file 1 — Data S1. [file MEC-33-e17580-s001.zip › Supplementary_v3/Appendices_25_July_2024.docx]

**Supplementary Appendices.**

**Appendix S1. Comparison of closest populations for gene flow estimates**

To compare adjacent populations, we selected a subset of individuals and populations used in clade-based comparisons for each of the 21 comparisons performed (Table 1). We selected the following pairs of clades:

(1) Clade 1 (*S. minor*) vs. Clade 9 (*S. ornatus*): we used the single population of Clade 1 and the two adjacent populations of Clade 9.

(2)  Clade 1 (*S. minor*) vs. Clade 10 (*S. ornatus*): we used the single population of Clade 1 and the southwesternmost population of Clade 10 (SML 132, 134, 136).

(3) Clade 1 (*S. minor*) vs. Clade 12 (*S. oberon*): we used the single population of Clade 1 and the southernmost populations of Clade 12 (JJW 644–646, 648, 665–666, 668–669). (4) Clade 1 (*S. minor*) vs. Clade 11 (*S. oberon*): we used the single population of Clade 1 and for Clade 11 we used the southwesternmost population (JJW 532–536).

(5) Clade 1 (*S. minor*) vs. Clade 2 (*S. minor*): we used the single population of Clade 1 and for Clade 2 we used the westernmost populations (JJW 700, 702–705).

(6) Clade 1 (*S. minor*) vs. Clade 3 (*S. minor*): we used the single population of Clade 1 and for Clade 3 we used both populations JJW 580–584 (only one individual from population from Zacatecas).

(7) Clade 2 (*S. minor*) vs. Clade 3 (*S. minor*): for Clade 2 we used the westernmost populations (JJW 700, 702–705) and for Clade 3 we used the easternmost population (JJW 580–584).

(8) Clade 2 (*S. minor*) vs. Clade 11 (*S. oberon*): for Clade 2 we used the northernmost populations (SML 162; JJW 708–710, 712) and for Clade 11 we used the southernmost population (SML 158–160).

(9) Clade 2 (*S. minor*) vs. Clade 6 (*Sceloporus* sp.): for Clade 2 we used the southernmost populations (JJW 587–588, 590) and Clade 6 consists of a single sampled population. (10) Clade 2 (*S. minor*) vs. Clade 7 (*Sceloporus* sp.): for Clade 2 we used the southernmost populations (JJW 587–588, 590) and Clade 7 is a single sampled population.

(11) Clade 3 (*S. minor*) vs. Clade 4 (*S. minor*): for Clade 3 we used the southernmost populations (JJW 580–584) and for Clade 4 we used the northernmost population (EPR 741, 743).

(12) Clade 3 (*S. minor*) vs. Clade 7 (*Sceloporus* sp.): for Clade 3 we used the southernmost population (JJW 580–584) and Clade 7 is a single sampled population.

(13) Clade 4 (*S. minor*) vs. Clade 7 (*Sceloporus* sp.): for Clade 4 we used the northernmost population (EPR 741, 743) and Clade 7 is a single sampled population.

(14) Clade 5 (*S. cyanogenys*) vs. Clade 12 (*S. oberon*): for Clade 5 we used the two southwestern populations (JJW 593, 620) and for Clade 12 we used the easternmost population (JW 603–606).

(15) Clade 5 (*S. cyanogenys*) vs. Clade 10 (*S. ornatus*): for Clade 5 we used two southwestern populations (JJW 593, 620) and for Clade 10 we used the easternmost population (JJW 627–629).

(16) Clade 6 (*Sceloporus* sp.) vs. Clade 7 (*Sceloporus* sp.): each clade is represented by a single population.

(17) Clade 8 (S*. cyanostictus*) vs. Clade 9 (*S. ornatus*): for Clade 8 we used the westernmost population (JJW 564) and for Clade 9 we used the westernmost population (SML 128–131).

(18) Clade 8 (*S. cyanostictus*) vs. Clade 10 (*S. ornatus*): for Clade 8 we used the easternmost population (SML 137, 139) and for Clade 10 we used the northernmost population in Coahuila (JJW 677).

(19) Clade 9 (*S. ornatus*) vs. Clade 10 (*S. ornatus*): for Clade 9 we used the easternmost population (SML 128–131) and for Clade 10 we used the westernmost populations (SML 132, 134, 136).

(20) Clade 10 (*S. ornatus*) vs. Clade 12 (*S. oberon*): for Clade 10 we used southeastern populations in Coahuila (JJW 670–672) and for Clade 12 we used northernmost populations (JJW 683–687).

(21) Clade 11 (*S. oberon*) vs. Clade 12 (*S. oberon*): we used the northernmost population of Clade 11 (SML 149–151) and a set of nearby southernmost populations of Clade 12 (JJW 644–646, 648, 665–666, 668–669).

**Appendix S2. Morphological character definitions**

We used the morphological dataset from Wiens and Penkrot (2002), from their Appendix 4. Each character is divided into three categories (squamation, coloration, morphometric) and the original literature source for each character is cited (references below). Note that these references refer to the precedent for the use of the character in a systematic study: they are not the original source of the data. Coloration and morphometric characters were based on males only to reduce potential impacts of sexual dimorphism (in color, size, and shape) and unequal sampling of males and females among populations. Squamation characters were scored for both sexes.

*Squamation*

1. Subnasal-postrostral contact: (0) absent, (1) present (Wiens and Reeder, 1997).

2. Postrostral number (Smith, 1939).

3. Posterior postrostral number (Wiens and Reeder, 1997).

4. Number of anterior frontonasals (Wiens and Reeder, 1997).

5. Contact between lateral and median frontonasals: (0) absent, (1) present (Smith, 1939).

6. Frontals, saggital division: (0) absent, (1) present (Smith, 1939).

7. Frontal-interparietal contact: (0) absent, (1) present (Smith, 1939).

8. Frontal-median frontonasal contact: (0) absent, (1) present (Smith, 1939).

9. Median prefrontal: (0) absent, (1) present (Smith, 1939).

10. Median parietal: (0) absent, (1) present (Smith, 1939).

11. Frontoparietal number (Smith, 1939).

12. Posterior division of the interparietal: (0) absent, (1) present (Wiens and Reeder, 1997).

13. Supraocular-frontoparietal contact: (0) absent, (1) present (Smith, 1939).

14. Number of supraocular scales (all scales between the circumorbitals and superciliaries were counted).

15. Canthal-lorilabial contact: (0) absent, (1) present (Smith, 1939).

16. Preocular division: (0) absent, (1) present (Smith, 1939).

17. Loreal number (Smith, 1939).

18. Number of gular scales (from the level of the mitepockets up to and including the mental scale)

19. Number of dorsals (Smith, 1939).

20. Number of femoral pores per side (Smith, 1939).

21. Number of scales between femoral pore rows (Smith, 1939).

22. Number of interpostanals (males only; Wiens and Reeder, 1997).

23. Scales around forelimb (measured at midlength of forearm).

*Coloration*

25. Head and nuchal and region (in males): (0) not black, (1) black.

26. Width of black nuchal collar (number of scales; modified from Smith, 1939).

27. White posterior border of dark nuchal collar (in males): (0) incomplete mid-dorsally, (1) complete mid-dorsally (Smith, 1939).

28. Lateral blue spot in nuchal collar (in males): (0) absent, (1) present (Smith, 1939)

29. White nuchal spots (in males): (0) absent, (1) present (Smith, 1939).

30. White nuchal stripe (in males): (0) absent, (1) present (Smith, 1939).

31. White postocular stripe (in males): (0) absent, (1) present (Smith, 1939).

32. Black axillary spot (in males): (0) absent, (1) present (Wiens and Reeder, 1997).

33. Black on anterior portion of some or all flank scales (in males): (0) absent, (1) present.

34. Black transverse stripes on flanks (in males): (0) absent, (1) present.

35. Blue gular blotch (in males): (0) extends over entire gular region, (1) on posterior portion of gular region only (Smith, 1939).

36. Black ventral collar (in males): (0) absent, (1) present (Smith, 1939).

37. Contact between belly patches and gular patch (in males): (0) absent, (1) present (Smith, 1939).

38. Median contact of belly patches (in males): (0) absent, (1) present (Smith, 1939).

39. Belly patches (in males): (0) not extending onto hindlimbs, (1) extending onto hindlimbs (Smith, 1939).

40. Black medial border of belly patches (in males): (0) absent, (1) present (Smith, 1939)

*Morphometric*

42. Maximum male SVL.

43. Residual from regression of hindlimb vs. SVL (males only).

44. Residual from regression of head length vs. SVL (males only).

**References (not in the main text)**

Wiens, J. J., and T. W. Reeder. 1997. Phylogeny of the spiny lizards (*Sceloporus*) based on molecular and morphological evidence. *Herpetological Monographs* 11:1–101.

**Appendix S3. Comparison of populations for morphological divergence analyses**

**Adjacent population comparisons**: To compare adjacent populations, we selected the populations that were geographically closest from the dataset of Wiens and Penkrot (2002). We explain the selection of populations below.

(1) Clade 1 (*S. minor*) vs. Clade 9 (*S. ornatus*): we used the single population of Clade 1 and the single morphology-based population of *S. ornatus*.

(2)  Clade 1 (*S. minor*) vs. Clade 10 (*S. ornatus*): we used the single population of Clade 1 and the single morphology-based population of *S. ornatus*.

(3) Clade 1 (*S. minor*) vs. Clade 12 (*S. oberon*): we used the single population of Clade 1 and the northern morphology-based population of Clade 12 (oberon-19 of WP; Clade-12_1 here)).

(4) Clade 1 (*S. minor*) vs. Clade 11 (*S. oberon*): we used the single population of Clade 1 and for Clade 11 we used the more southern population (minor-9 of WP; Clade 11_1 here).

(5) Clade 1 (*S. minor*) vs. Clade 2 (*S. minor*): we used the single population of Clade 1 and for Clade 2 we used the more northern population of Clade 2 (minor-13 of WP; Clade2_1 here), which is closer to Clade 1.

6) Clade 1 (*S. minor*) vs. Clade 3 (*S. minor*): we used the single sampled population of Clade 1 and for Clade 3 we used the northwesternmost population from Zacatecas (minor-18 of WP; Clade3_2), which is marginally closer to Clade 1.

(7) Clade 2 (*S. minor*) vs. Clade 3 (*S. minor*): for Clade 2 we used the westernmost population with morphological data (minor-13 of WP; Clade2_1 here) and for Clade 3 we used the northwesternmost population from Zacatecas (minor-18 of WP; Clade3_2), which is marginally closer to Clade 2.

(8) Clade 2 (*S. minor*) vs. Clade 11 (*S. oberon*): for Clade 2 we used the northernmost population with morphological data (minor-13 of WP; Clade2_1 here) and for Clade 11 we used the southernmost population (minor-12 of WP; Clade11_2 here).

(9) Clade 2 (*S. minor*) vs. Clade 6 (*Sceloporus* sp.): for Clade 2 we used the southernmost population with morphological data (minor-16 of WP; Clade2_2 here) and Clade 6 consists of a single sampled population.

(10) Clade 2 (*S. minor*) vs. Clade 7 (*Sceloporus* sp.): for Clade 2 we used the southernmost population with morphological data (minor-16 of WP; Clade2_2 here) and Clade 7 consists of a single sampled population.

(11) Clade 3 (*S. minor*) vs. Clade 4 (*S. minor*): for Clade 3 we used the southernmost populations (minor-15 of WP; Clade3–1 here) and for Clade 4 we used the most well-sampled northernmost population with morphological data (erythrocyaneus-3 of WP; Clade4_1 here).

(12) Clade 3 (*S. minor*) vs. Clade 7 (*Sceloporus* sp.): for Clade 3 we used the westernmost population (minor-15 of WP; Clade3_1 here) and Clade 7 is a single sampled population.

(13) Clade 4 (*S. minor*) vs. Clade 7 (*Sceloporus* sp.): for Clade 4 we used the

most well-sampled northernmost population with morphological data (erythrocyaneus-3 of WP; Clade4_1 here). Clade 7 is a single sampled population.

(14) Clade 5 (*S. cyanogenys*) vs. Clade 12 (*S. oberon*): for Clade 5 we used the available morphological data for *S. cyanogenys* and for Clade 12 we used the easternmost population (from which the molecular data were obtained; oberon-20 in WP; Clade 12_2 here).

(15) Clade 5 (*S. cyanogenys*) vs. Clade 10 (*S. ornatus*): for Clade 5 we used the available morphological data for *S. cyanogenys* and for Clade 10 we used the used the available morphological data for *S. ornatus.*

(16) Clade 6 (*Sceloporus* sp.) vs. Clade 7 (*Sceloporus* sp.): each clade is represented by a single population.

(17) Clade 8 (*S. cyanostictus*) vs. Clade 9 (*S. ornatus*): for Clade 8 we used the westernmost population (cyanostictus-2 of WP; Clade 8-2 here) and for Clade 9

we used the used the available morphological data for *S. ornatus.*

(18) Clade 8 (*S. cyanostictus*) vs. Clade 10 (*S. ornatus*): for Clade 8 we used the easternmost population (cyanostictus-1 of WP; Clade 8-1 here) and for Clade 10 we used the used the available morphological data for *S. ornatus.*

(19) Clade 9 (*S. ornatus*) vs. Clade 10 (*S. ornatus*): We had morphological data for only one population of *S. ornatus*.

(20) Clade 10 (*S. ornatus*) vs. Clade 12 (*S. oberon*): for Clade 10 we used the used the available morphological data for *S. ornatus* and for Clade 12 we used northernmost the populations of *S. oberon* sampled for morphological data (oberon-19 of WP; Clade12-1 here).

(21) Clade 11 (*S. oberon*) vs. Clade 12 (*S. oberon*): we used the northernmost population of Clade 11 with morphological data (minor-10 of WP; Clade11_2 here) and a set of nearby southernmost populations of Clade 12 (minor-11 of WP; Clade12_3 here).

**Clade-level comparison.** We used the morphological dataset of Wiens and Penkrot (2002) and matched these to the molecular-based clades identified here. When morphological data from multiple populations were available from the same molecular-based clade, we averaged mean population values across populations to obtain a single value for each clade. The populations that were combined are explained below, along with the match to the data from Wiens and Penkrot (2002). Numbered populations are from Wiens and Penkrot (2002). When describing populations sampled in that paper, we followed the taxonomy used in that paper, which treated most species as subspecies of *Sceloporus jarrovii*.

Clade 1: matched: *S. j. minor* population 17.

Clade 2: averaged *S. j. minor* populations 13 and minor 16.

Clade 3: averaged *S. j. minor* populations 15 and minor 18.

Clade 4: averaged *erythocyaneus* populations 3 and 4 and immucronatus population 5.

Clade 5: *S. cyanogenys*.

Clade 6: *S. j. immucronatus* population 6

Clade 7: matched: *S. j. minor* population 14.

Clade 8: averaged *S. j. cyanostictus* populations 1 and 2.

Clades 9 and 10: used morphological data for *S. ornatus caeruleus*.

Clade 11: averaged *S. j. minor* populations 9, 10, and 12.

Clade 12: averaged *S. j. minor* population 11 and *S. j. oberon* populations 19 and 20.

**Appendix S4. Multispecies coalescent analysis of focal *Sceloporus***

We performed a coalescent-based phylogenetic analysis among species and populations using SNAPPER (Stolz et al., 2020). We performed five SNAPPER analyses (Table S2). We initially included only *S. poinsettii* as an outgroup, using two or three individuals per clade (see below). We focused on *S. poinsettii* given that it is the closest outgroup to the ingroup in the concatenated analyses (Fig. 1). However, in our initial analyses using SNAPPER, we found that *S. poinsetii* was placed in the ingroup, and that its placement within the ingroup differed strongly depending on whether two or three individuals were sampled per tip/species (Fig. S1B vs. S1E). Therefore, we also conducted analyses that included all four outgroup taxa (*S. jarrovii, S. poinsettii, S. sugillatus, S. torquatus*) and two individuals per tip. This analysis also placed *S. poinsettii* within the ingroup, with the other three outgroup taxa placed as outgroups (Fig. S1D). We also conducted an analysis in which we used all outgroups except *S. poinsettii*. In this analysis, *S. sugillatus* was placed in the ingroup instead, in the same position (with *S. cyanogenys*) where *S. poinsettii* was placed in the two-tip analyses (Fig. S1C). Given these results, it appeared that the placement of the outgroup taxa was unreliable. Therefore, we also conducted an analysis with no outgroups (Fig. S1A). This is our preferred analysis and the one we present in the main text (Fig. 2). All trees in Fig. S1 and Fig, 2 were rooted automatically in SNAPPER which infers species trees directly by estimating the probability of allele frequency change across ancestor to descendent branches.

To prepare our ddRADseq data for SNAPPER analysis, we used the *vcftophylip.py* python script (Ortiz, 2019) and exported the binary data for input into SNAPPER. We used BEAUti to format input XML files and place individuals in clades for the analysis. Given the time and computational power required for these analyses, we used only 2 individuals (one set of analyses) or 3 (separate set) to represent each of the 12 clades identified from the concatenated analyses, to reduce the computational burden of including many taxa. For these analyses, we selected the most complete 2–3 individuals from Clades 1–12. We included the following individuals (bolded individuals were those used in the two-individuals-per-tip analyses): JJW 634, **635, 637** (Clade 1); **SML 162, JJW 590**, 704 (Clade 2); **JJW 581, 582,** 579 (Clade 3); **EPR 743, JJW 720**, 721 (Clade 4); **TWR 243, JJW 593**, 620 (Clade 5); **JJW 517**, 518, **519** (Clade 6); **JJW 507, 509**, 511 (Clade 7); **SML 137, 139**, JJW 564 (Clade 8); **SML 128, 129**, 130 (Clade 9); **SML 134, JJW 627**, 672 (Clade 10); **SML 151, 154**, JJW 653 (Clade 11); **SML 142, 146**, 147 (Clade 12).

After 1 million generations of sampling, we discarded the first 100 trees as burnin prior to summarizing tree sets and checking effective sample sizes (ESSs) using the tracerer R function (Bilderbeek & Etienne, 2018). We found that in all five SNAPPER analyses some parameters did not reach an ESS of >100 (e.g. some snapper coalescent rate parameters). However, the key parameters of likelihood and posterior distributions had ESSs >200 in all cases (Table S2). Note that these analyses were extremely time intensive, even when using a very large high-performance computer cluster.

Posterior probabilities for each of the five analyses are superimposed on a representative tree from the last generation of each analysis (Fig. S1). The five SNAPPER analyses were congruent in that they all recovered three of the deep clades from the concatenated analysis including Clades 1–4 (I), Clades 8–10 (III), and Clades 11–12 (IV). The three individuals per tip analysis (Fig. S1, E) also recovered deep clade II in some trees, albeit with low support (posterior probability=0.44).

As noted above, we observed outgroup taxa often appeared within the ingroup. In the analyses in which only *S. poinsettii* was used as an outgroup, this species was placed in a well-supported clade with either *S. cyanogenys* (two individuals per tip, Fig. S1, B) or with *S. oberon* (three individuals per tip, Fig. S1, E). Branch support (posterior probabilities) for these relationships ranged from 0.88 to 0.03. When additional outgroups were included, *S. poinsettii* was also in a clade with *S. cyanogenys*, whereas the other outgroups were found to be sister to the ingroup samples with moderate support for the monophyly of the ingroup clade (0.86, Fig. S1, D). When *S. poinsettii* was removed and the other outgroups were left in the analysis (Fig. S1, C), *S. sugillatus* was placed in a well-supported clade with *S. cyanogenys* with high support (0.99). Despite high posterior probabilities placing some putative outgroup species with the ingroup, there are clearly issues with these results. Most notably, the contrasting placements of *S. poinsettii* and *S. sugillatus* in the ingroup (or outgroup) in different positions strongly suggest that the analyses with outgroups are suspect. Therefore, in the main text we present the analyses excluding all outgroups.

We acknowledge that there is some precedent for *S. poinsettii* being placed in the ingroup by coalescent analyses of targeted sequence capture data (Fig. 2b of Leache et al., 2016) but not in the concatenated analyses (Fig. 2a). However, these relationships were not generally well supported, that study generally included only a single individual per species, and key taxa analyzed here were missing (including *S. oberon* and *S. sugillatus*, with only one *S. minor* individual sampled).

**References (not in the main text)**

Ortiz, E. M. (2019). vcf2phylip v2.0: convert a VCF matrix into several matrix formats for phylogenetic analysis. DOI:10.5281/zenodo.2540861._
