# Supplementary material for: What Predicts Gene Flow During Speciation? The Relative Roles of Time, Space, Morphology and Climate
Source: Mol Ecol. 2024 Nov 7;33(23):e17580. doi: 10.1111/mec.17580 (PMC11589662; doi:10.1111/mec.17580)
Supplement: Supplementary file 1 — Data S1. [file MEC-33-e17580-s001.zip › Supplementary_v3/Figure_Legends_Supp_October_2024.docx]

**Supplementary Figure Legends**

FIGURE S1 Posterior probabilities of clades from SNAPPER coalescent analyses mapped on the last tree from the sampling. Analyses differed in containing only ingroups (A), *S. poinsettii* as an outgroup (B), *S. sugillatus* + *S. torquatus* + *S. jarrovii* as outgroups (C), *S. poinsettii* + *S. sugillatus* + *S. torquatus* + *S. jarrovii* as outgroups (D), and *S. poinsettii* as an outgroup when three individuals per tip were used (E). Analyses A, B, C, and D utilized two individuals per tip. Analyses are described in more detail in Table S2.

FIGURE S2 Two dimensional results of Principal Component (PC) analysis results for deep clade I (*Sceloporus minor* [Clades 1, 2, 3, and 4]), based on 74,343 SNPs. Plots are PC1 versus PC2 (left) and PC1 versus PC3 (right).

FIGURE S3 Two dimensional results of Principal Component (PC) analysis results for deep clade II (*Sceloporus cyanogenys + Sceloporus* spp. [Clades 5, 6, and 7]), based on 74,305 SNPs. Plots are PC1 versus PC2 (left) and PC1 versus PC3 (right).

FIGURE S4 Two dimensional results of Principal Component (PC) analysis results for deep clade III (*Sceloporus cyanostictus + S. ornatus* [Clades 8, 9, and 10]), based on 74,263 SNPs. Plots are PC1 versus PC2 (left) and PC1 versus PC3 (right).

FIGURE S5 Two dimensional results of Principal Component (PC) analysis results for deep clade IV (*Sceloporus oberon* [Clades 11 and 12]), based on 74,344 SNPs. Plots are PC1 versus PC2 (left) and PC1 versus PC3 (right).

FIGURE S6 Comparison of missing data thresholds for no more than 50% missing data per SNP (24,547 SNPs, A) and a more permissive missing data strategy based on 74,343 SNPs (B) for deep clade I (*Sceloporus minor* [Clades 1, 2, 3, and 4]). Note similarity in clustering patterns.

FIGURE S7 Comparison of missing data thresholds for no more than 50% missing data per SNP (11,809 SNPs, A) and a more permissive missing data strategy based on 74,305 SNPs (B) for deep clade II (*Sceloporus cyanogenys + Sceloporus* spp. [Clades 5, 6, and 7]). Note similarity in clustering patterns.

FIGURE S8 Comparison of missing data thresholds for no more than 50% missing data per SNP (15,849 SNPs, A) and a more permissive missing data strategy based on 74,263 SNPs (B) for deep clade III (*Sceloporus cyanostictus + S. ornatus* [Clades 8, 9, and 10]). Note similarity in clustering patterns.

FIGURE S9 Comparison of missing data thresholds for no more than 50% missing data per SNP (17,318 SNPs, A) and a more permissive missing data strategy based on 74,344 SNPs (B) for deep clade IV (*Sceloporus oberon* [Clades 11 and 12]). Note similarity in clustering patterns.

FIGURE S10 Comparison of clade-based geographic distances and population-based distances demonstrating significant correlation (*rho*=0.83, *p*<0.0001).

FIGURE S11 Bar plot from STRUCTURE analysis used to estimate gene flow for comparison 1; Clade 1 (*Sceloporus minor*) to Clade 9 (*S. ornatus*).

FIGURE S12 Bar plot from STRUCTURE analysis used to estimate gene flow for comparison 2; Clade 1 (*Sceloporus minor*) to Clade 10 (*S. ornatus*).

FIGURE S13 Bar plot from STRUCTURE analysis used to estimate gene flow for comparison 3; Clade 1 (*Sceloporus minor*) to Clade 12 (*S. oberon*).

FIGURE S14 Bar plot from STRUCTURE analysis used to estimate gene flow for comparison 4; Clade 1 (*Sceloporus minor*) to Clade 11 (*S. oberon*).

FIGURE S15 Bar plot from STRUCTURE analysis used to estimate gene flow for comparison 5; Clade 1 (*Sceloporus minor*) to Clade 2 (*S. minor*).

FIGURE S16 Bar plot from STRUCTURE analysis used to estimate gene flow for comparison 6; Clade 1 (*Sceloporus minor*) to Clade 3 (*S. minor*).

FIGURE S17 Bar plot from STRUCTURE analysis used to estimate gene flow for comparison 7; Clade 2 (*Sceloporus minor*) to Clade 3 (*S. minor*).

FIGURE S18 Bar plot from STRUCTURE analysis used to estimate gene flow for comparison 8; Clade 2 (*Sceloporus minor*) to Clade 11 (*S. oberon*).

FIGURE S19 Bar plot from STRUCTURE analysis used to estimate gene flow for comparison 9; Clade 2 (*Sceloporus minor*) to Clade 6 (*Sceloporus* sp.).

FIGURE S20 Bar plot from STRUCTURE analysis used to estimate gene flow for comparison 10; Clade 2 (*Sceloporus minor*) to Clade 7 (*Sceloporus* sp.).

FIGURE S21 Bar plot from STRUCTURE analysis used to estimate gene flow for comparison 11; Clade 3 (*Sceloporus minor*) to Clade 4 (*S. minor*).

FIGURE S22 Bar plot from STRUCTURE analysis used to estimate gene flow for comparison 12; Clade 3 (*Sceloporus minor*) to Clade 7 (*Sceloporus* sp.).

FIGURE S23 Bar plot from STRUCTURE analysis used to estimate gene flow for comparison 13; Clade 4 (*Sceloporus minor*) to Clade 7 (*Sceloporus* sp.).

FIGURE S24 Bar plot from STRUCTURE analysis used to estimate gene flow for comparison 14; Clade 5 (*Sceloporus cyanogenys*) to Clade 12 (*S. oberon*).

FIGURE S25 Bar plot from STRUCTURE analysis used to estimate gene flow for comparison 15; Clade 5 (*Sceloporus cyanogenys*) to Clade 10 (*S. ornatus*).

FIGURE S26 Bar plot from STRUCTURE analysis used to estimate gene flow for comparison 16; Clade 6 (*Sceloporus* sp.) to Clade 7 (*Sceloporus* sp.).

FIGURE S27 Bar plot from STRUCTURE analysis used to estimate gene flow for comparison 17; Clade 8 (*Sceloporus cyanostictus*) to Clade 9 (*S. ornatus*).

FIGURE S28 Bar plot from STRUCTURE analysis used to estimate gene flow for comparison 18; Clade 8 (*Sceloporus cyanostictus*) to Clade 10 (*S. ornatus*).

FIGURE S29 Bar plot from STRUCTURE analysis used to estimate gene flow for comparison 19; Clade 9 (*Sceloporus ornatus*) to Clade 10 (*S. ornatus*).

FIGURE S30 Bar plot from STRUCTURE analysis used to estimate gene flow for comparison 20; Clade 10 (*Sceloporus ornatus*) to Clade 12 (*S. oberon*).

FIGURE S31 Bar plot from STRUCTURE analysis used to estimate gene flow for comparison 21; Clade 11 (*Sceloporus oberon*) to Clade 12 (*S. oberon*).

FIGURE S32 Plots resulting from OptM analysis for TREEMIX indicating that M=7 is the optimal number of migration edges in the *Sceloporus* dataset.

FIGURE S33 TREEMIX result displaying the optimal number of migration edges (M=7) determined using OptM. Migration weight is equivalent to the genomic proportion shared across the edge.

FIGURE S34 Comparison of clade-based morphological distances and population-based morphological distances demonstrating significant correlation (*rho*=0.89, *p*<0.001).

FIGURE S35 Comparison of clade-based climatic distances and population-based climatic distances demonstrating significant correlation (*rho*=0.88, *p*<0.001).

FIGURE S36 Clade admixture (mean frequency) and morphological divergence levels with S. *ornatus* (Clade 9) to *S. ornatus* (Clade 10) comparison removed. The relationship is negative but not significant (*p*=0.306).

FIGURE S37 Significant results of Bayesian generalized linear model analysis between mean admixture (clade-based) and time (A) and morphology (B) when comparisons containing *Sceloporus cyanogenys* and *S. cyanostictus* are removed. Each data point is one of the 17 comparisons of pairs of clades. Gray shading around regression lines corresponds to smoothed conditional means. In the time versus mean admixture plot (A), data point (hexagon) shading corresponds to the number of data points occupying a given hexagon.
