# Supplementary material for: What Predicts Gene Flow During Speciation? The Relative Roles of Time, Space, Morphology and Climate
Source: Mol Ecol. 2024 Nov 7;33(23):e17580. doi: 10.1111/mec.17580 (PMC11589662; doi:10.1111/mec.17580)
Supplement: Supplementary file 1 — Data S1. [file MEC-33-e17580-s001.zip › Supplementary_v3/Tables_S1-S8_25_July_2024.docx]

**Supplementary Tables S1–S8.**

**Table S1** Locality data for *Sceloporus* specimens used in this study.

**Table S2** Sampling strategy for five multispecies coalescent analyses conducted in SNAPPER.

**Table S3** Results of PCA with percent variance explained for the first ten PCs of the multivariate analysis of the ddRADseq data.

**Table S4** Results for Spearmans correlation between PCA and amount of missing data across individuals in the ddRADseq dataset.

**Table S5** Component loadings for PCA of the clade-based morphological data.

**Table S6** Component loadings for PCA of the population-based morphological data*.*

**Table S7** Component loadings for the clade-based PCA of the climate data.

**Table S8** Component loadings for the population-based PCA of the climate data.

**Table S1** Locality data for *Sceloporus* specimens used in this study. Population designations are taken from Lambert et al. (2019).

| **Individual** | **Species** | **Clade** | **Population** | **Locality** | **Latitude** | **Longitude** | **Museum ID** |
| --- | --- | --- | --- | --- | --- | --- | --- |
|  |  |  |  |  |  |  |  |
| TWR 243 | *cyanogenys* | 5 | cyanogenys | USA: Texas: 9.5 mi. E of the jct. of TX 16 and F. M. 1962, on the Caron Ranch | 28.2118 | -98.4068 | LSUMZ 48852 |
| JJW 593 | *cyanogenys* | 5 | cyanogenys1 | Nuevo Leon: hillside above Horsetail Falls (Salto Cola de Caballo) ca 12 km W Santiago on road to Laguna Sanchez rocky hillside in deciduous forest under large rock crevice | 25.36326 | -100.193 | NA |
| JJW 620 | *cyanogenys* | 5 | cyanogenys2 | Nuevo Leon: Santiago, ca 7 km E Hwy 85 on road to Cadereyta; hillside near dam of Presa Rodrigo Gomez (La Boca) – mountain range on east side of valley on rock faces of road cut in thornscrub | 25.4304 | -100.124 | MZFC 19148 |
| UOGV 2018 | *cyanostictus* | 8 | cuatrocienegas | Coahuila: mountains southwest of the town of Cuatrocienegas | 26.788 | -102.446 | NA |
| UOGV 2019 | *cyanostictus* | 8 | cuatrocienegas | Coahuila: mountains southwest of the town of Cuatrocienegas Cuatrocienegas | 26.788 | -102.446 | NA |
| SML 137 | *cyanostictus* | 8 | cyanostictus | Coahuila: Hwy 57: ca. 12 km south of turnoff for Las Potrancas | 26.304013 | -101.347 | NA |
| SML 139 | *cyanostictus* | 8 | cyanostictus | Coahuila: Hwy 57: ca. 12Km south of turnoff for Las Potrancas | 26.304013 | -101.347 | NA |
| JJW 564 | *cyanostictus* | 8 | cyanostictus1 | Coahuila: 1.0 km S (by road) of San Lorenzo (SW of San Pedro de los Colonias); on rocky hillside in Chihuahuan desert | 25.712226 | -103.156 | NA |
| EPR 741 | *minor* | 4 | minor1 | Queretaro: near Pinal de Amoles | 21.1364 | -99.65 | NA |
| EPR 743 | *minor* | 4 | minor1 | Queretaro: near Pinal de Amoles | 21.1364 | -99.65 | NA |
| JJW 587 | *minor* | 2 | minor10 | Tamaulipas: 33 km NE northern turnoff for Tula on Hwy 101 just W turnoff for Bustamenta; road cut in juniper-oak woodland | 23.2928 | -99.6647 | CM 147656 |
| JJW 587 | *minor* | 2 | minor10 | Tamaulipas: 33 km NE northern turnoff for Tula on Hwy 101 just W turnoff for Bustamenta; road cut in juniper-oak woodland | 23.2928 | -99.6647 | CM 147656 |
| JJW 588 | *minor* | 2 | minor10 | Tamaulipas: 33 km NE northern turnoff for Tula on Hwy 101 just W turnoff for Bustamenta; road cut in juniper-oak woodland | 23.2928 | -99.6647 | NA |
| JJW 590 | *minor* | 2 | minor10 | Tamaulipas: 35 km NE northern turnoff for Tula on Hwy 101 road cut in mixed woodland (oak, juniper, etc) | 23.2928 | -99.6647 | CM 147657 |
| JJW 634 | *minor* | 1 | minor11 | Zacatecas: ca 4 km W Concepcion del Oro on road to Mazapil 8.4 km W turnoff for Concepcion del Oro from Hwy 54 in rock crevices in rock outcrops on rocky hillside in Chihuahuan desert scrub | 24.6120 | -101.4420 | CM 147660 |
| JJW 635 | *minor* | 1 | minor11 | Zacatecas: ca 4 km W Concepcion del Oro on road to Mazapil 8.4 km W turnoff for Concepcion del Oro from Hwy 54 in rock crevices in rock outcrops on rocky hillside in Chihuahuan desert scrub | 24.6120 | -101.4420 | CM 147661 |
| JJW 636 | *minor* | 1 | minor11 | Zacatecas: ca 4 km W Concepcion del Oro on road to Mazapil 8.4 km W turnoff for Concepcion del Oro from Hwy 54 in rock crevices in rock outcrops on rocky hillside in Chihuahuan desert scrub | 24.6120 | -101.4420 | NA |
| JJW 637 | *minor* | 1 | minor11 | Zacatecas: ca 4 km W Concepcion del Oro on road to Mazapil 8.4 km W turnoff for Concepcion del Oro from Hwy 54 in rock crevices in rock outcrops on rocky hillside in Chihuahuan desert scrub | 24.6120 | -101.4420 | NA |
| JJW 639 | *minor* | 1 | minor11 | Zacatecas: ca 4 km W Concepcion del Oro on road to Mazapil 8.4 km W turnoff for Concepcion del Oro from Hwy 54 in rock crevices in rock outcrops on rocky hillside in Chihuahuan desert scrub | 24.6120 | -101.4420 | NA |
| JJW 700 | *minor* | 2 | minor12 | San Luis Potosi: 18.9 km E Matehuela (from Hwy 57) on rd. to Dr. Arroyo: 1 km W border between S.L.P. and Nuevo Leon | 23.6892 | -100.4719 | CM 147678 |
| JJW 702 | *minor* | 2 | minor12 | Nuevo Leon: 22.8 km E Matehuela (from Hwy 57) on road to Dr. Arroyo | 23.6850 | -100.4375 | CM 147679 |
| JJW 703 | *minor* | 2 | minor12 | Nuevo Leon: 22.8 km E Matehuela (from Hwy 57) on road to Dr. Arroyo | 23.6850 | -100.4375 | CM 147680 |
| JJW 704 | *minor* | 2 | minor12 | Nuevo Leon: 22.8 km E Matehuela (from Hwy 57) on road to Dr. Arroyo | 23.6850 | -100.4375 | NA |
| JJW 705 | *minor* | 2 | minor12 | San Luis Potosi: 18.9 km E Matehuela (from Hwy 57) on rd. to Dr. Arroyo: 1 km W border between S.L.P. and Nuevo Leon | 23.6892 | -100.4719 | NA |
| JJW 708 | *minor* | 2 | minor13 | Nuevo Leon: 7.1 km N Dr. Arroyo (Plaza de Armas) on rd. to Galeana | 23.7111 | -100.1361 | NA |
| JJW 709 | *minor* | 2 | minor13 | Nuevo Leon: 7.1 km N Dr. Arroyo (Plaza de Armas) on rd. to Galeana | 23.7111 | -100.1361 | NA |
| JJW 710 | *minor* | 2 | minor13 | Nuevo Leon: 7.1 km N Dr. Arroyo (Plaza de Armas) on rd. to Galeana | 23.7111 | -100.1361 | NA |
| JJW 712 | *minor* | 2 | minor13 | Nuevo Leon: 7.1 km N Dr. Arroyo (Plaza de Armas) on rd. to Galeana | 23.7111 | -100.1361 | CM 147682 |
| JJW 716 | *minor* | 4 | minor14 | Queretaro: 4.9k m S Ezequil Montes on Hwy 120 between Tequisquiapan and Cadereyta rocky hillside/road in grassland area w/ scattered huizatche and *Opuntia* trees | 20.64306 | -99.945 | CM 147683 |
| JJW 717 | *minor* | 4 | minor14 | Queretaro: 4.9k m S Ezequil Montes on Hwy 120 between Tequisquiapan and Cadereyta rocky hillside/road in grassland area w/ scattered huizatche and *Opuntia* trees | 20.64306 | -99.945 | CM 147684 |
| JJW 718 | *minor* | 4 | minor14 | Queretaro: 4.9k m S Ezequil Montes on Hwy 120 between Tequisquiapan and Cadereyta rocky hillside/road in grassland area w/ scattered huizatche and *Opuntia* trees | 20.64306 | -99.945 | MZFC 10735 |
| JJW 720 | *minor* | 4 | minor14 | Queretaro: 4.9k m S Ezequil Montes on Hwy 120 between Tequisquiapan and Cadereyta rocky hillside/road in grassland area w/ scattered huizatche and *Opuntia* trees | 20.64306 | -99.945 | NA |
| JJW 721 | *minor* | 4 | minor15 | Queretaro: 1 km S Cadereyta on Hwy 120; rocky hillside w/ rock outcrops in area of desert scrub | 20.7102 | -99.8340 | CM 147685 |
| JJW 722 | *minor* | 4 | minor15 | Queretaro: 1 km S Cadereyta on Hwy 120; rocky hillside w/ rock outcrops in area of desert scrub | 20.7102 | -99.8340 | MZFC 10738 |
| JJW 723 | *minor* | 4 | minor15 | Queretaro: 1 km S Cadereyta on Hwy 120; rocky hillside w/ rock outcrops in area of desert scrub | 20.7102 | -99.8340 | NA |
| JJW 492 | *minor* | 4 | minor2 | Hidalgo: Barranca de Los Marmoles, Hwy 85 W Jacala, ca 100m from turnoff for San Vicente; on rocks in pine-oak forest | 20.8069 | -99.2675 | CM 147625 |
| JJW 493 | *minor* | 4 | minor2 | Hidalgo: Barranca de Los Marmoles, Hwy 85 W Jacala, ca 100m from turnoff for San Vicente; on rocks in pine-oak forest | 20.8069 | -99.2675 | CM 147626 |
| JJW 494 | *minor* | 4 | minor2 | Hidalgo: Barranca de Los Marmoles, Hwy 85 W Jacala, ca 100m from turnoff for San Vicente; on rocks in pine-oak forest | 20.8069 | -99.2675 | CM 147627 |
| JJW 495 | *minor* | 4 | minor2 | Hidalgo: Barranca de Los Marmoles, Hwy 85 W Jacala, ca 100m from turnoff for San Vicente; on rocks in pine-oak forest | 20.8069 | -99.2675 | NA |
| JJW 502 | *minor* | 4 | minor3 | Hidalgo: Barranca de los Marmotes along Hwy 85 W of Jacala on rocks in pine-oak forest | 20.8069 | -99.2675 | NA |
| JJW 504 | *minor* | 4 | minor4 | Hidalgo: Puerto de la Zorra, between Cuesta Colorado and Jacala near El Pinalito on Hwy 85; on rocks in oak-juniper forest | 21.0247 | -99.1325 | CM 147628 |
| JJW 506 | *minor* | 4 | minor5 | Hidalgo: Puerto de la Zorra, between Cuesta Colorado and Jacala near El Pinalito on Hwy 85; on rocks in oak-juniper forest | 21.0247 | -99.1325 | NA |
| JJW 507 | *Sceloporus* sp. | 7 | minor6 | San Luis Potosi: 14.1 km E Ciudad del Maiz on Hwy 80, road between El Naranjo and Cuidad del Maiz; rocky hillside in desert scrub | 22.4886 | -99.565 | CM 147630 |
| JJW 508 | *Sceloporus* sp. | 7 | minor6 | San Luis Potosi: 14.1 km E Ciudad del Maiz on Hwy 80, road between El Naranjo and Cuidad del Maiz; rocky hillside in desert scrub | 22.4886 | -99.565 | CM 147631 |
| JJW 509 | *Sceloporus* sp. | 7 | minor6 | San Luis Potosi: 14.1 km E Ciudad del Maiz on Hwy 80, road between El Naranjo and Cuidad del Maiz; rocky hillside in desert scrub | 22.4886 | -99.565 | NA |
| JJW 511 | *Sceloporus* sp. | 7 | minor6 | San Luis Potosi: 14.1 km E Ciudad del Maiz on Hwy 80, road between El Naranjo and Cuidad del Maiz; rocky hillside in desert scrub | 22.4886 | -99.565 | NA |
| JJW 512 | *Sceloporus* sp. | 7 | minor6 | San Luis Potosi: 14.1 km E Ciudad del Maiz on Hwy 80, road between El Naranjo and Cuidad del Maiz; rocky hillside in desert scrub | 22.4886 | -99.565 | CM 147632 |
| JJW 513 | *Sceloporus* sp. | 7 | minor6 | San Luis Potosi: 14.1 km E Ciudad del Maiz on Hwy 80, road between El Naranjo and Cuidad del Maiz; rocky hillside in desert scrub | 22.4886 | -99.565 | NA |
| JJW 514 | *Sceloporus* sp. | 6 | minor7 | Tamaulipas: 16.9 km W Ciudad Victoria (nr km 151) on Hwy 101 on road to Jaumave at crest of Sierra Madre (east side); oak forest – rocky hillside | 23.6181 | -99.1925 | NA |
| JJW 515 | *Sceloporus* sp. | 6 | minor7 | Tamaulipas: 21.7 km W Ciudad Victoria (W km 151) on Hwy 101 on road to Jaumave at crest of Sierra Madre (west side); Restuarant “El Madrono”; oak forest, rocky hillside with *Agave* and *Opuntia* | 23.6089 | -99.2225 | NA |
| JJW 516 | *Sceloporus* sp. | 6 | minor7 | Tamaulipas: 21.7 km W Ciudad Victoria (W km 151) on Hwy 101 on road to Jaumave at crest of Sierra Madre (west side); Restuarant “El Madrono”; oak forest, rocky hillside with *Agave* and *Opuntia* | 23.6089 | -99.2225 | CM 147633 |
| JJW 517 | *Sceloporus* sp. | 6 | minor7 | Tamaulipas: 21.7 km W Ciudad Victoria (W km 151) on Hwy 101 on road to Jaumave at crest of Sierra Madre (west side); Restuarant “El Madrono”; oak forest, rocky hillside with *Agave* and *Opuntia* | 23.6089 | -99.2225 | CM 147634 |
| JJW 518 | *Sceloporus* sp. | 6 | minor7 | Tamaulipas: 21.7 km W Ciudad Victoria (W km 151) on Hwy 101 on road to Jaumave at crest of Sierra Madre (west side); Restuarant “El Madrono”; oak forest, rocky hillside with *Agave* and *Opuntia* | 23.6089 | -99.2225 | CM 147635 |
| JJW 519 | *Sceloporus* sp. | 6 | minor7 | Tamaulipas: 21.7 km W Ciudad Victoria (W km 151) on Hwy 101 on road to Jaumave at crest of Sierra Madre (west side); Restuarant “El Madrono”; oak forest, rocky hillside with *Agave* and *Opuntia* | 23.6089 | -99.2225 | NA |
| JJW 579 | *minor* | 3 | minor8 | Zacatecas: 6 km NW Saldana (turnoff for Saldana) on Hwy 49 between Zacatecas and San Luis Potosi; ca. 8 km NW border between Zacatecas and San Luis Potosi; rock crevices on rocky hillside in mesquite-grassland | 22.4272 | -101.463 | MZFC 10692 |
| JJW 580 | *minor* | 3 | minor9 | San Luis Potosi: Colonia Insurgentes, ca. 2.5 km W San Luis Potosi on Hwy 80 (road to Ojuelos de Jalisco), 2.5 km W road to Hwy 57; rocky hillside with huizatche, *Yucca*, and *Opuntia* | 22.1500 | -100.9833 | CM 147653 |
| JJW 581 | *minor* | 3 | minor9 | San Luis Potosi: Colonia Insurgentes, ca. 2.5 km W San Luis Potosi on Hwy 80 (rd to Ojuelos de Jalisco), 2.5 km W road to Hwy 57; rocky hillside with huizatche, *Yucca*, and *Opuntia* | 22.1500 | -100.9833 | CM 147654 |
| JJW 582 | *minor* | 3 | minor9 | San Luis Potosi: Colonia Insurgentes, ca. 2.5 km W San Luis Potosi on Hwy 80 (rd to Ojuelos de Jalisco), 2.5 km W road to Hwy 57; rocky hillside with huizatche, *Yucca*, and *Opuntia* | 22.1500 | -100.9833 | NA |
| JJW 583 | *minor* | 3 | minor9 | San Luis Potosi: Colonia Insurgentes, ca. 2.5 km W San Luis Potosi on Hwy 80 (rd to Ojuelos de Jalisco), 2.5 km W road to Hwy 57; rocky hillside with huizatche, *Yucca*, and *Opuntia* | 22.1500 | -100.9833 | CM 147655 |
| JJW 584 | *minor* | 3 | minor9 | San Luis Potosi: Colonia Insurgentes, ca. 2.5 km W San Luis Potosi on Hwy 80 (rd to Ojuelos de Jalisco), 2.5 km W road to Hwy 57; rocky hillside with huizatche, *Yucca*, and *Opuntia* | 22.1500 | -100.9833 | MZFC 10694 |
| SML 162 | *minor* | 2 | minorS1 | Nuevo Leon: Hwy 61, ca. 4 km S of turnoff for Aramberri | 24.07096 | -99.9474 | NA |
| JJW 532 | *oberon* | 11 | oberon1 | Nuevo Leon; 9.1 km E San Roberto on Hwy 58 on wall of arroyo, earth (not rock); surrounding vegetation Joshua trees, pinyon pine, creosote bush | 24.68825 | -100.206 | CM 147638 |
| JJW 533 | *oberon* | 11 | oberon1 | Nuevo Leon; 9.1 km E San Roberto on Hwy 58 on wall of arroyo, earth (not rock); surrounding vegetation Joshua trees, pinyon pine, creosote bush | 24.68825 | -100.206 | CM 147639 |
| JJW 534 | *oberon* | 11 | oberon1 | Nuevo Leon; 9.1 km E San Roberto on Hwy 58 on wall of arroyo, earth (not rock); surrounding vegetation Joshua trees, pinyon pine, creosote bush | 24.68825 | -100.206 | CM 147640 |
| JJW 535 | *oberon* | 11 | oberon1 | Nuevo Leon; 9.1 km E San Roberto on Hwy 58 on wall of arroyo, earth (not rock); surrounding vegetation Joshua trees, pinyon pine, creosote bush | 24.68825 | -100.206 | NA |
| JJW 536 | *oberon* | 11 | oberon1 | Nuevo Leon; 9.1 km E San Roberto on Hwy 58 on wall of arroyo, earth (not rock); surrounding vegetation Joshua trees, pinyon pine, creosote bush | 24.68825 | -100.206 | NA |
| JJW 683 | *oberon* | 12 | oberon10 | Coahuila: crest of sierra north of town of El Diamante dry rocky hillside (desert scrub) surrounded by pine forest (pinyon primarily) | 25.37 | -100.872 | CM 147673 |
| JJW 684 | *oberon* | 12 | oberon10 | Coahuila: crest of sierra north of town of El Diamante dry rocky hillside (desert scrub) surrounded by pine forest (pinyon primarily) | 25.37 | -100.872 | CM 147674 |
| JJW 685 | *oberon* | 12 | oberon10 | Coahuila: crest of sierra north of town of El Diamante dry rocky hillside (desert scrub) surrounded by pine forest (pinyon primarily) | 25.37 | -100.872 | NA |
| JJW 686 | *oberon* | 12 | oberon10 | Coahuila: crest of sierra north of town of El Diamante dry rocky hillside (desert scrub) surrounded by pine forest (pinyon primarily) | 25.37 | -100.872 | NA |
| JJW 687 | *oberon* | 12 | oberon10 | Coahuila: crest of sierra north of town of El Diamante dry rocky hillside (desert scrub) surrounded by pine forest (pinyon primarily) | 25.37 | -100.872 | NA |
| JJW 690 | *oberon* | 12 | oberon11 | Nuevo Leon: 2.1 km S Santa Clara de Cienega (19.9 km SW by road from turnoff for road between Hwy 57 and San Antonio de las Alazanas); dry rocky hillside and rock fence with sotol and lechuguilla in valley between mountains with pine forest | 25.18667 | -100.477 | CM 147675 |
| JJW 691 | *oberon* | 12 | oberon11 | Nuevo Leon: 2.1 km S Santa Clara de Cienega (19.9 km SW by road from turnoff for road between Hwy 57 and San Antonio de las Alazanas); dry rocky hillside and rock fence with sotol and lechuguilla in valley between mountains with pine forest | 25.18667 | -100.477 | MZFC 10725 |
| JJW 692 | *oberon* | 12 | oberon11 | Nuevo Leon: 2.1 km S Santa Clara de Cienega (19.9 km SW by road from turnoff for road between Hwy 57 and San Antonio de las Alazanas); dry rocky hillside and rock fence with sotol and lechuguilla in valley between mountains with pine forest | 25.18667 | -100.477 | CM 147676 |
| JJW 693 | *oberon* | 12 | oberon11 | Nuevo Leon: 2.1 km S Santa Clara de Cienega (19.9 km SW by road from turnoff for road between Hwy 57 and San Antonio de las Alazanas); dry rocky hillside and rock fence with sotol and lechuguilla in valley between mountains with pine forest | 25.18667 | -100.477 | MZFC 10726 |
| JJW 694 | *oberon* | 12 | oberon11 | Nuevo Leon: 2.1 km S Santa Clara de Cienega (19.9 km SW by road from turnoff for road between Hwy 57 and San Antonio de las Alazanas); dry rocky hillside and rock fence with sotol and lechuguilla in valley between mountains with pine forest | 25.18667 | -100.477 | CM 147677 |
| JJW 695 | *oberon* | 12 | oberon11 | Nuevo Leon: 2.1 km S Santa Clara de Cienega (19.9 km SW by road from turnoff for road between Hwy 57 and San Antonio de las Alazanas); dry rocky hillside and rock fence with sotol and lechuguilla in valley between mountains with pine forest | 25.18667 | -100.477 | MZFC 10727 |
| JJW 696 | *oberon* | 12 | oberon11 | Nuevo Leon: 2.1 km S Santa Clara de Cienega (19.9 km SW by road from turnoff for road between Hwy 57 and San Antonio de las Alazanas); dry rocky hillside and rock fence with sotol and lechuguilla in valley between mountains with pine forest | 25.18667 | -100.477 | NA |
| JJW 697 | *oberon* | 12 | oberon11 | Nuevo Leon: 2.1 km S Santa Clara de Cienega (19.9 km SW by road from turnoff for road between Hwy 57 and San Antonio de las Alazanas); dry rocky hillside and rock fence with sotol and lechuguilla in valley between mountains with pine forest | 25.18667 | -100.477 | NA |
| JJW 538 | *oberon* | 12 | oberon2 | Coahuila: 22.3 km E by road from San Antonio de Las Alazanas on rocks in road cut in pine forest | 25.25 | -100.408 | CM 147641 |
| JJW 539 | *oberon* | 12 | oberon2 | Coahuila: 22.3 km E by road from San Antonio de Las Alazanas on rocks in road cut in pine forest | 25.25 | -100.408 | CM 147642 |
| JJW 540 | *oberon* | 12 | oberon2 | Coahuila: 22.3 km E by road from San Antonio de Las Alazanas on rocks in road cut in pine forest | 25.25 | -100.408 | CM 147643 |
| JJW 541 | *oberon* | 12 | oberon2 | Coahuila: 22.3 km E by road from San Antonio de Las Alazanas on rocks in road cut in pine forest | 25.25 | -100.408 | NA |
| JJW 542 | *oberon* | 12 | oberon2 | Coahuila: 22.3 km E by road from San Antonio de Las Alazanas on rocks in road cut in pine forest | 25.25 | -100.408 | NA |
| JJW 543 | *oberon* | 12 | oberon2 | Coahuila: 22.3 km E by road from San Antonio de Las Alazanas on rocks in road cut in pine forest | 25.25 | -100.408 | NA |
| JJW 544 | *oberon* | 12 | oberon2 | Coahuila: 22.3 km E by road from San Antonio de Las Alazanas on rocks in road cut in pine forest | 25.25 | -100.408 | NA |
| JJW 603 | *oberon* | 12 | oberon3 | Nuevo Leon: 2.5 km E San Isidro (turnoff for Laguna Sanchez on Santiago-Saltillo rd. Nuevo Leon Hwy 20); on rock walls of steep-sided canyon in dry pine forest area | 25.35705 | -100.307 | MZFC 10698 |
| JJW 604 | *oberon* | 12 | oberon3 | Nuevo Leon: 2.5 km E San Isidro (turnoff for Laguna Sanchez on Santiago-Saltillo rd. Nuevo Leon Hwy 20); on rock walls of steep-sided canyon in dry pine forest area | 25.35705 | -100.307 | CM 147658 |
| JJW 605 | *oberon* | 12 | oberon3 | Nuevo Leon: 2.5 km E San Isidro (turnoff for Laguna Sanchez on Santiago-Saltillo rd. Nuevo Leon Hwy 20); on rock walls of steep-sided canyon in dry pine forest area | 25.35705 | -100.307 | CM 147659 |
| JJW 606 | *oberon* | 12 | oberon3 | Nuevo Leon: 2.5 km E San Isidro (turnoff for Laguna Sanchez on Santiago-Saltillo rd. Nuevo Leon Hwy 20); on rock walls of steep-sided canyon in dry pine forest area | 25.35705 | -100.307 | NA |
| JJW 644 | *oberon* | 12 | oberon4 | Nuevo Leon: 10 km E Hwy 57 (San Rafael) on road to San Pablo-Cienega del Toro; road cut in open pinyon forest | 25.0625 | -100.478 | NA |
| JJW 645 | *oberon* | 12 | oberon5 | Nuevo Leon: 12.6 km E Hwy 57 (San Rafael) on road to San Pablo-Cienega del Toro | 25.06931 | -100.457 | MZFC 10707 |
| JJW 646 | *oberon* | 12 | oberon6 | Nuevo Leon: 4 km E San Pablo on road to Cienega del Toro; road cut in open pinyon-pine forest | 25.07444 | -100.386 | CM 147664 |
| JJW 648 | *oberon* | 12 | oberon6 | Nuevo Leon: 4 km E San Pablo on road to Cienega del Toro; road cut in open pinyon-pine forest | 25.07444 | -100.386 | NA |
| JJW 650 | *oberon* | 11 | oberon7 | Nuevo Leon: Pablillo (S of Galeana on Nuevo Leon Hwy 67); rock wall surrounding pasture at edge of town; surrounding hills with pine-oak forest | 24.59128 | -99.9955 | CM 147665 |
| JJW 651 | *oberon* | 11 | oberon7 | Nuevo Leon: Pablillo (S of Galeana on Nuevo Leon Hwy 67); rock wall surrounding pasture at edge of town; surrounding hills with pine-oak forest | 24.59128 | -99.9955 | CM 147666 |
| JJW 652 | *oberon* | 11 | oberon7 | Nuevo Leon: Pablillo (S of Galeana on Nuevo Leon Hwy 67); rock wall surrounding pasture at edge of town; surrounding hills with pine-oak forest | 24.59128 | -99.9955 | CM 147667 |
| JJW 653 | *oberon* | 11 | oberon7 | Nuevo Leon: Pablillo (S of Galeana on Nuevo Leon Hwy 67); rock wall surrounding pasture at edge of town; surrounding hills with pine-oak forest | 24.59128 | -99.9955 | NA |
| JJW 654 | *oberon* | 11 | oberon7 | Nuevo Leon: Pablillo (S of Galeana on Nuevo Leon Hwy 67); rock wall surrounding pasture at edge of town; surrounding hills with pine-oak forest | 24.59128 | -99.9955 | NA |
| JJW 660 | *oberon* | 11 | oberon8 | Nuevo Leon: 3.1 – 7.4 km N Pablillo on Nuevo Leon Hwy 67; road cut in pine forest | 24.65056 | -100.028 | CM 147669 |
| JJW 661 | *oberon* | 11 | oberon8 | Nuevo Leon: 3.1 – 7.4 km N Pablillo on Nuevo Leon Hwy 67; road cut in pine forest | 24.65056 | -100.028 | MZFC 10716 |
| JJW 662 | *oberon* | 11 | oberon8 | Nuevo Leon: 3.1 – 7.4 km N Pablillo on Nuevo Leon Hwy 67; road cut in pine forest | 24.65056 | -100.028 | MZFC 10717 |
| JJW 663 | *oberon* | 11 | oberon8 | Nuevo Leon: 3.1 – 7.4 km N Pablillo on Nuevo Leon Hwy 67; road cut in pine forest | 24.65056 | -100.028 | NA |
| JJW 664 | *oberon* | 11 | oberon8 | Nuevo Leon: 3.1 – 7.4 km N Pablillo on Nuevo Leon Hwy 67; road cut in pine forest | 24.65056 | -100.028 | CM 147670 |
| JJW 665 | *oberon* | 12 | oberon9 | Nuevo Leon: 0.8 km W San Pablo, 19.7 km E San Rafael (turnoff from Hwy 57); rock outcrop in grassland area surrounded by open pinyon-pine forest | 25.07583 | -100.415 | MZFC 10719 |
| JJW 666 | *oberon* | 12 | oberon9 | Nuevo Leon: 0.8 km W San Pablo, 19.7 km E San Rafael (turnoff from Hwy 57); rock outcrop in grassland area surrounded by open pinyon-pine forest | 25.07583 | -100.415 | CM 147671 |
| JJW 668 | *oberon* | 12 | oberon9 | Nuevo Leon: 0.8 km W San Pablo, 19.7 km E San Rafael (turnoff from Hwy 57); rock outcrop in grassland area surrounded by open pinyon-pine forest | 25.07583 | -100.415 | CM 147672 |
| JJW 669 | *oberon* | 12 | oberon9 | Nuevo Leon: 0.8 km W San Pablo, 19.7 km E San Rafael (turnoff from Hwy 57); rock outcrop in grassland area surrounded by open pinyon-pine forest | 25.07583 | -100.415 | NA |
| SML 142 | *oberon* | 12 | oberonS1 | Coahuila: ca 1.5 km S of San Antonio de Las Alazanas along gravel road, <1km N of border with Nuevo Leon | 25.25413 | -100.576 | NA |
| SML 143 | *oberon* | 12 | oberonS2 | Nuevo Leon: ca 2 km S Santa Clara de Cienega | 25.18608 | -100.477 | NA |
| SML 144 | *oberon* | 12 | oberonS2 | Nuevo Leon: ca 2 km S Santa Clara de Cienega | 25.18608 | -100.477 | NA |
| SML 145 | *oberon* | 12 | oberonS2 | Nuevo Leon: ca 2 km S Santa Clara de Cienega | 25.18608 | -100.477 | NA |
| SML 146 | *oberon* | 12 | oberonS2 | Nuevo Leon: ca 2 km S Santa Clara de Cienega | 25.18608 | -100.477 | NA |
| SML 147 | *oberon* | 12 | oberonS2 | Nuevo Leon: ca 2 km S Santa Clara de Cienega | 25.18608 | -100.477 | NA |
| SML 149 | *oberon* | 11 | oberonS3 | Nuevo Leon: ca 7 km S of Cienega del Toro | 25.0287 | -100.305 | NA |
| SML 150 | *oberon* | 11 | oberonS3 | Nuevo Leon: ca 7 km S of Cienega del Toro | 25.0287 | -100.305 | NA |
| SML 151 | *oberon* | 11 | oberonS3 | Nuevo Leon: ca 7 km S of Cienega del Toro | 25.0287 | -100.305 | NA |
| SML 152 | *oberon* | 11 | oberonS4 | Nuevo Leon: ca 2.5 km N by road of Dieciocho de Marzo | 24.9038 | -100.196 | NA |
| SML 153 | *oberon* | 11 | oberonS4 | Nuevo Leon: ca 2.5 km N by road of Dieciocho de Marzo | 24.9038 | -100.196 | NA |
| SML 154 | *oberon* | 11 | oberonS4 | Nuevo Leon: ca 2.5 km N by road of Dieciocho de Marzo | 24.9038 | -100.196 | NA |
| SML 156 | *oberon* | 11 | oberonS5 | Nuevo Leon: ca 8.6 km N of Galeana on road to Rayones | 24.88588 | -100.087 | NA |
| SML 158 | *oberon* | 11 | oberonS6 | Nuevo Leon: Hwy 61, ca 11 km S of Pablillo | 24.52398 | -99.9885 | NA |
| SML 159 | *oberon* | 11 | oberonS6 | Nuevo Leon: Hwy 61, ca 11 km S of Pablillo | 24.52398 | -99.9885 | NA |
| SML 160 | *oberon* | 11 | oberonS6 | Nuevo Leon: Hwy 61, ca 11 km S of Pablillo | 24.52398 | -99.9885 | NA |
| JJW 627 | *ornatus* | 10 | ornatus1 | Nuevo Leon: Sierra El Fraile, 1.3 km S Grutas de Garcia; rocky hillside and rock cliffs in Chihuahuan desert scrub | 25.84998 | -100.53 | MFZC 11230 |
| JJW 628 | *ornatus* | 10 | ornatus1 | Nuevo Leon: Sierra El Fraile, 1.3 km S Grutas de Garcia; rocky hillside and rock cliffs in Chihuahuan desert scrub | 25.84998 | -100.53 | MFZC 11231 |
| JJW 629 | *ornatus* | 10 | ornatus1 | Nuevo Leon: Sierra El Fraile, 1.3 km S Grutas de Garcia; rocky hillside and rock cliffs in Chihuahuan desert scrub | 25.84998 | -100.53 | MFZC 11232 |
| JJW 670 | *ornatus* | 10 | ornatus2 | Coahuila: 2.8 km N turnoff for Ramos Arizpe on Hwy 57 between Saltillo and Monclova (182 km S Monclova); rock outcrop/rocky hillside in Chihuahuan desert scrub | 25.5417 | -101.023 | MFZC 11238 |
| JJW 671 | *ornatus* | 10 | ornatus2 | Coahuila: 2.8 km N turnoff for Ramos Arizpe on Hwy 57 between Saltillo and Monclova (182 km S Monclova); rock outcrop/rocky hillside in Chihuahuan desert scrub | 25.5417 | -101.023 | NA |
| JJW 672 | *ornatus* | 10 | ornatus2 | Coahuila: 2.8 km N turnoff for Ramos Arizpe on Hwy 57 between Saltillo and Monclova (182 km S Monclova); rock outcrop/rocky hillside in Chihuahuan desert scrub | 25.5417 | -101.023 | MFZC 11239 |
| JJW 673 | *ornatus* | 10 | ornatus3 | Coahuila: San Miguel, 17.4 km N turnoff for Ramos Arizpe on Hwy 57 (Saltillo-Monclova); rock outcrop/rocky hillside in Chihuahuan desert scrub | 25.6 | -101.1 | MFZC 11240 |
| JJW 674 | *ornatus* | 10 | ornatus3 | Coahuila: San Miguel, 17.4 km N turnoff for Ramos Arizpe on Hwy 57 (Saltillo-Monclova); rock outcrop/rocky hillside in Chihuahuan desert scrub | 25.6 | -101.1 | MFZC 11241 |
| JJW 675 | *ornatus* | 10 | ornatus3 | Coahuila: San Miguel, 17.4 km N turnoff for Ramos Arizpe on Hwy 57 (Saltillo-Monclova); rock outcrop/rocky hillside in Chihuahuan desert scrub | 25.6 | -101.1 | MFZC 11242 |
| JJW 676 | *ornatus* | 10 | ornatus3 | Coahuila: San Miguel, 17.4 km N turnoff for Ramos Arizpe on Hwy 57 (Saltillo-Monclova); rock outcrop/rocky hillside in Chihuahuan desert scrub | 25.6 | -101.1 | MFZC 11243 |
| JJW 677 | *ornatus* | 10 | ornatus4 | Coahuila: 4 km N Fraustro, 55.5 km N turnoff from Ramos Arizpe on Hwy 57 (Saltillo-Monclova); rock outcrop/rocky hillside in Chihuahuan desert scrub | 25.9225 | -101.158 | MFZC 11244 |
| NM 167 | *ornatus* | 10 | ornatus5 | Coahuila: Ojo Caliente, Municipio de Ramos Arizpe | 25.6271 | -100.838 | IBH 18038 |
| NM 170 | *ornatus* | 10 | ornatus5 | Coahuila: Ojo Caliente, Municipio de Ramos Arizpe | 25.6271 | -100.838 | IBH 18041 |
| NM 171 | *ornatus* | 10 | ornatus5 | Coahuila: Ojo Caliente, Municipio de Ramos Arizpe | 25.6271 | -100.838 | IBH 18042 |
| NM 173 | *ornatus* | 10 | ornatus5 | Coahuila: Ojo Caliente, Municipio de Ramos Arizpe | 25.6271 | -100.838 | IBH 18044 |
| NM 175 | *ornatus* | 10 | ornatus6 | Coahuila: north of Saltillo, Coahuila, next to kilometer 17 of Hwy 57 between Saltillo-Monclova, a 1 km north of the turnoff for San Martín de las Vacas | 25.55213 | -101.029 | IBH 18046 |
| NM 176 | *ornatus* | 10 | ornatus6 | Coahuila: north of Saltillo, Coahuila, next to kilometer 17 of Hwy 57 between Saltillo-Monclova, a 1 km north of the turnoff for San Martín de las Vacas | 25.55213 | -101.029 | IBH 18047 |
| NM 177 | *ornatus* | 10 | ornatus6 | Coahuila: north of Saltillo, Coahuila, next to kilometer 17 of Hwy 57 between Saltillo-Monclova, a 1 km north of the turnoff for San Martín de las Vacas | 25.55213 | -101.029 | IBH 18048 |
| NM 178 | *ornatus* | 10 | ornatus6 | Coahuila: north of Saltillo, Coahuila, next to kilometer 17 of Hwy 57 between Saltillo-Monclova, a 1 km north of the turnoff for San Martín de las Vacas | 25.55213 | -101.029 | IBH 18049 |
| JAM 652 | *ornatus* | ? | ornatusJAM | Coahuila, Cerro El Sol, 0.4 mi. N of Hwy 40, 10.5 mi. E of the intersection of Hwy 40 and Hwy 31 | 25.62195 | -102.637 | NA |
| SML 126 | *ornatus* | ? | ornatusS1 | Coahuila: ca. 1 km S of pulloff on Hwy 40 (Saltillo-Matamoros), 1.5km E of Casa de Cobro “La Cuchilla” | 25.61782 | -102.864 | NA |
| SML 128 | *ornatus* | 9 | ornatusS1 | Coahuila: ca. 1 km S of pulloff on Hwy 40 (Saltillo-Matamoros), 1.5km E of Casa de Cobro “La Cuchilla” | 25.61782 | -102.864 | NA |
| SML 129 | *ornatus* | 9 | ornatusS1 | Coahuila: ca. 1 km S of pulloff on Hwy 40 (Saltillo-Matamoros), 1.5km E of Casa de Cobro “La Cuchilla” | 25.61782 | -102.864 | NA |
| SML 130 | *ornatus* | 9 | ornatusS1 | Coahuila: ca. 1 km S of pulloff on Hwy 40 (Saltillo-Matamoros), 1.5km E of Casa de Cobro “La Cuchilla” | 25.61782 | -102.864 | NA |
| SML 131 | *ornatus* | 9 | ornatusS1 | Coahuila: ca. 1 km S of pulloff on Hwy 40 (Saltillo-Matamoros), 1.5km E of Casa de Cobro “La Cuchilla” | 25.61782 | -102.864 | NA |
| SML 132 | *ornatus* | 10 | ornatusS2 | Coahuila: ca. 27 km W from General Cepeda on Hwy 105 | 25.32583 | -101.704 | NA |
| SML 134 | *ornatus* | 10 | ornatusS3 | Coahuila: ca 24.5km W from General Cepeda on Hwy 105 | 25.32749 | -101.686 | NA |
| SML 136 | *ornatus* | 10 | ornatusS3 | Coahuila: ca 24.5 km W from General Cepeda on Hwy 105 | 25.32749 | -101.686 | NA |
| JJW 571 | *jarrovii* | Outgroup | NA | Zacatecas: Sierra Fresnillo, E of Colonia Guanajuato, ca. 24 km W Fresnillo (by road) road between Fresnillo and Sauceda | not georeferenced | not georeferenced | CM 147651 |
| TWR 938 | *poinsettii* | Outgroup | NA | Texas: Brewster Co.: ~5 mi SW of Marathon. | not georeferenced | not georeferenced | NA |
| JJW 485 | *sugillatus* | Outgroup | NA | Morelos: Lagos de Zempoala (Lago #4) on Hwy 95 W Huitzilac (east of other lakes) on large rocks at W end of lake/pine forest | not georeferenced | not georeferenced | CM 147623 |
| SML 155 | *torquatus* | Outgroup | NA | Nuevo Leon: ca 8.6 km N of Galeana on road to Rayones | 24.88588 | -100.087 | NA |
|  |  |  |  |  |  |  |  |

**Table S2** Sampling strategy for five multispecies coalescent analyses conducted in SNAPPER. Effective sample sizes (ESS) were calculated after removing 100 generations as burnin.

| **Tree Fig. S1** | **No. taxa** | **No. sites** | **No. patterns** | **Outgroups** | **Individuals per tip** | **ESS likelihood** | **ESS posterior** |
| --- | --- | --- | --- | --- | --- | --- | --- |
|  |  |  |  |  |  |  |  |
| A | 24 | 58,551 | 22,704 | None | Two | 1537.9 | 1515.5 |
| B | 25 | 58,551 | 24,529 | *poinsettii* | Two | 295.8 | 286.2 |
| C | 27 | 58,058 | 29,378 | *sugillatus*, *torquatus*, *jarrovii* | Two | 526.0 | 514.33 |
| D | 28 | 58,058 | 31,142 | *poinsettii*, *sugillatus*, *torquatus*, *jarrovii* | Two | 343.1 | 325.6 |
| E | 37 | 58,551 | 36,342 | *poinsettii* | Three | 830.4 | 824.3 |
|  |  |  |  |  |  |  |  |

**Table S3** Results of PCA with percent variance explained for the first ten PCs of the multivariate analysis of the ddRADseq data. Clade I = *S. minor*, Clade II = *S. cyanogenys* + *Sceloporus* spp., Clade III = *S. cyanostictus* + S *ornatus*, Clade IV = *S. oberon*.

| **Dataset** | **PC1** | **PC2** | **PC3** | **PC4** | **PC5** | **PC6** | **PC7** | **PC8** | **PC9** | **PC10** |
| --- | --- | --- | --- | --- | --- | --- | --- | --- | --- | --- |
|  |  |  |  |  |  |  |  |  |  |  |
| Clade I | 12.1 | 8.8 | 5.6 | 4.7 | 4.2 | 3.5 | 3.3 | 3.3 | 3.1 | 3.0 |
| Clade II | 20.8 | 17.6 | 7.5 | 7.0 | 6.4 | 6.2 | 5.8 | 5.6 | 5.2 | 4.9 |
| Clade III | 15.9 | 7.8 | 7.1 | 6.2 | 5.7 | 4.2 | 4.1 | 4.0 | 3.7 | 3.6 |
| Clade IV | 13.4 | 6.6 | 3.8 | 3.4 | 3.1 | 2.9 | 2.4 | 2.3 | 2.3 | 2.2 |
|  |  |  |  |  |  |  |  |  |  |  |

**Table S4** Results for Spearmans correlation between PCA and amount of missing data across individuals in the ddRADseq dataset. Clade I = *S. minor*, Clade II = *S. cyanogenys* + *Sceloporus* spp., Clade III = *S. cyanostictus* + S *ornatus*, Clade IV = *S. oberon*.

| **Dataset** | **PC1** | **PC2** | **PC3** | **PC4** |
| --- | --- | --- | --- | --- |
|  |  |  |  |  |
| Clade I | S = 6545, *rho* = -0.08, p = 0.663 | **S = 8540, *rho* = -0.43, *p* = 0.012** | S = 5924, *rho* = 0.01, *p* = 0.956 | S = 5794, *rho* = 0.03, p = 0.861 |
| Clade II | S = 440, *rho* = 0.03, p = 0.916 | S = 340, *rho* = 0.25, *p* = 0.383 | S = 634, *rho* = -0.39, *p* = 0.165 | S = 240, *rho* = 0.47, *p* = 0.09 |
| Clade III | **S = 3678, rho = -0.41, p =0.040** | **S = 3720, *rho* = -0.43, *p* = 0.031** | S = 2310, *rho* = 0.11, *p* = 0.594 | **S = 1570, *rho* = 0.40, *p* = 0.051** |
| Clade IV | S = 15076, *rho* = 0.23, *p* = 0.111 | S = 22438, *rho* = -0.14, *p* = 0.320 | S = 21548, *rho* = -0.10, *p* = 0.406 | S = 21756, *rho* = -0.11, *p* = 0.451 |
|  |  |  |  |  |

**Table S5** Component loadings for PCA of the clade-based morphological data*.* See Appendix S1 for definitions of morphological characters.

| **Character** | **PC1** | **PC2** | **PC3** | **PC4** |
| --- | --- | --- | --- | --- |
|  |  |  |  |  |
| 1 | -0.08935 | 0.011295 | -0.03663 | -0.28045 |
| 2 | -0.00129 | 0.002478 | -0.00154 | 0.000676 |
| 3 | 1.76E-05 | -0.00126 | 0.000379 | 0.001276 |
| 4 | -0.00015 | 0.000299 | 0.001324 | 0.001857 |
| 5 | -0.01077 | -0.08122 | -0.04273 | -0.03565 |
| 6 | 0.02002 | -0.04818 | -0.01627 | -0.11743 |
| 7 | 0.029937 | 0.155356 | 0.13684 | 0.041026 |
| 8 | -0.03949 | 0.073441 | 0.048682 | -0.00976 |
| 9 | -0.00044 | 0.0069 | -0.00078 | 0.011129 |
| 10 | -0.02589 | -0.35893 | -0.2139 | -0.16275 |
| 11 | -0.00014 | -0.00104 | -0.00014 | 0.000749 |
| 12 | -0.03779 | -0.20416 | -0.0197 | -0.23838 |
| 13 | 0.033596 | 0.00674 | 0.143946 | 0.008901 |
| 14 | -0.00131 | -0.03261 | 0.012891 | 0.00721 |
| 15 | -0.04564 | 0.154025 | -0.0153 | -0.20519 |
| 16 | 0.231058 | 0.015086 | -0.05467 | -0.2748 |
| 17 | 0.001201 | -0.00118 | -0.00142 | -0.00068 |
| 18 | 0.001928 | -0.00446 | -0.01322 | 0.005644 |
| 19 | 0.037197 | -0.02238 | -0.02512 | 0.011516 |
| 20 | 0.003211 | 0.01066 | 0.013926 | 0.009983 |
| 21 | -0.00066 | -0.01287 | -0.00751 | 0.009048 |
| 22 | 5.55E-05 | -0.00215 | -0.00182 | -0.00137 |
| 23 | 0.000475 | 0.000615 | 0.000919 | -0.00289 |
| 25 | -0.25273 | -0.39042 | 0.142002 | 0.171026 |
| 26 | -0.00014 | -0.01153 | -0.00515 | -0.00203 |
| 27 | 0.094335 | -0.25762 | -0.53986 | 0.210233 |
| 28 | -0.08762 | 0.038259 | -0.08274 | 0.303829 |
| 29 | 0.006787 | -0.01918 | -0.05699 | -0.03941 |
| 30 | 0.016618 | -0.01209 | 0.179869 | 0.035051 |
| 31 | -0.31701 | -0.42226 | 0.085504 | -0.01297 |
| 32 | -0.29141 | -0.24332 | 0.365952 | 0.342669 |
| 33 | 0.230399 | -0.0716 | 0.59844 | -0.13457 |
| 34 | 0 | 0 | 0 | 0 |
| 35 | -0.0139 | -0.00914 | 0.002683 | 0.01829 |
| 36 | -0.24464 | -0.19561 | 0.052758 | -0.56841 |
| 37 | 0.317348 | -0.29873 | -0.08598 | -0.08102 |
| 38 | 0.363853 | -0.29994 | 0.10177 | 0.025915 |
| 39 | 0.374885 | -0.27814 | 0.016545 | 0.042734 |
| 40 | -0.41037 | 0.01325 | -0.16869 | -0.07802 |
| 42 | -0.0868 | 0.077719 | 0.037493 | -0.24463 |
| 43 | -0.00721 | 0.017618 | -0.00245 | -0.01579 |
| 44 | -0.00043 | 0.001438 | -0.00523 | -0.00672 |
|  |  |  |  |  |
| % Variance | 45.60 | 15.53 | 12.04 | 9.03 |
|  |  |  |  |  |

**Table S6** Component loadings for PCA of the population-based morphological data*.* See Appendix S1 for definitions of morphological characters.

| **Character** | **PC1** | **PC2** | **PC3** | **PC4** |
| --- | --- | --- | --- | --- |
|  |  |  |  |  |
| 1 | -0.07076 | 0.029126 | -0.06314 | -0.03407 |
| 2 | -0.00055 | -0.00114 | -0.00356 | 0.004065 |
| 3 | -0.00042 | 0.001377 | 0.001077 | -0.00151 |
| 4 | 0.000416 | 0.000756 | 0.000935 | 0.001815 |
| 5 | 0.01006 | 0.127873 | -0.0271 | 0.043991 |
| 6 | -0.00822 | 0.077296 | 0.061889 | -0.15345 |
| 7 | 0.07875 | -0.07671 | 0.085263 | 0.186568 |
| 8 | -0.02538 | -0.03051 | 0.077765 | -0.06051 |
| 9 | -0.00331 | -0.02741 | -0.01445 | 0.015629 |
| 10 | -0.12656 | 0.32372 | 0.066293 | -0.44141 |
| 11 | -0.00019 | 7.70E-05 | 0.000271 | -0.00066 |
| 12 | 0.007299 | 0.283844 | -0.06779 | 0.073182 |
| 13 | 0.06413 | -0.03529 | 0.068181 | 0.157533 |
| 14 | 0.000809 | 0.017085 | 0.002924 | 0.017759 |
| 15 | -0.01788 | -0.00303 | 0.000804 | -0.04232 |
| 16 | 0.20261 | 0.066607 | 0.064224 | -0.38765 |
| 17 | 0.00069 | 0.000692 | -0.00019 | -0.00266 |
| 18 | 0.000444 | 0.00383 | -0.00944 | -0.00899 |
| 19 | 0.029108 | 0.007443 | -0.01598 | -0.02543 |
| 20 | 0.006748 | -0.00911 | 0.009471 | 0.00683 |
| 21 | -0.00196 | 0.010888 | -0.00751 | 0.002354 |
| 22 | 0.000105 | 0.003758 | -0.00053 | -0.0012 |
| 23 | 0.001136 | 0.001527 | 0.000791 | -0.00037 |
| 25 | -0.26577 | 0.383631 | 0.376562 | -0.01142 |
| 26 | -0.00284 | 0.005205 | -0.00132 | -0.00629 |
| 27 | 0.012391 | 0.233672 | -0.53368 | -0.07608 |
| 28 | -0.03486 | 0.030191 | -0.18821 | 0.349607 |
| 29 | -0.02669 | 0.015847 | 0.091346 | -0.07862 |
| 30 | 0.016802 | -0.00153 | 0.183959 | -0.00494 |
| 31 | -0.2924 | 0.433056 | 0.197935 | 0.134539 |
| 32 | -0.24956 | 0.035513 | 0.124894 | 0.535893 |
| 33 | 0.228204 | -0.15966 | 0.549528 | 0.085646 |
| 34 | 0 | 0 | 0 | 0 |
| 35 | -0.00982 | 0.003023 | 0.002324 | -0.00034 |
| 36 | -0.20958 | 0.259946 | 0.091165 | -0.09652 |
| 37 | 0.342518 | 0.303059 | -0.0661 | 0.079464 |
| 38 | 0.404787 | 0.298739 | 0.067699 | 0.190626 |
| 39 | 0.420494 | 0.327216 | -0.09677 | 0.188923 |
| 40 | -0.38179 | 0.015321 | -0.26795 | 0.140907 |
| 42 | -0.06933 | -0.06411 | 0.011392 | -0.04297 |
| 43 | -0.00844 | -0.0166 | -0.01039 | -0.00337 |
| 44 | -0.00197 | 0.001497 | 0.000879 | -0.00807 |
|  |  |  |  |  |
| % Variance | 31.22 | 17.19 | 14.46 | 8.28 |
|  |  |  |  |  |

**Table S7** Component loadings for the clade-based PCA of the climate data. BIO1 = Annual Mean Temperature, BIO2 = Mean Diurnal Range (Mean of monthly (max temp - min temp)), BIO3 = Isothermality (BIO2/BIO7) (* 100), BIO4 = Temperature Seasonality (standard deviation *100), BIO5 = Max Temperature of Warmest Month, BIO6 = Min Temperature of Coldest Month, BIO7 = Temperature Annual Range (BIO5-BIO6), BIO8 = Mean Temperature of Wettest Quarter, BIO9 = Mean Temperature of Driest Quarter, BIO10 = Mean Temperature of Warmest Quarter, BIO11 = Mean Temperature of Coldest Quarter, BIO12 = Annual Precipitation, BIO13 = Precipitation of Wettest Month, BIO14 = Precipitation of Driest Month, BIO15 = Precipitation Seasonality (Coefficient of Variation), BIO16 = Precipitation of Wettest Quarter, BIO17 = Precipitation of Driest Quarter, BIO18 = Precipitation of Warmest Quarter, BIO19 = Precipitation of Coldest Quarter. Bolded variables are the most heavily weighted on PCs 1 and 2.

| **Climate Variable** | **PC1** | **PC2** | **PC3** | **PC4** |
| --- | --- | --- | --- | --- |
|  |  |  |  |  |
| BIO10 | 0.002241 | 0.032869 | -0.04294 | 0.007917 |
| BIO19 | -0.03558 | 0.016292 | 0.446089 | -0.04065 |
| BIO16 | -0.42356 | 0.085648 | -0.56005 | 0.139411 |
| BIO2 | 0.003003 | -0.00915 | -0.01563 | -0.01013 |
| BIO4 | 0.205817 | 0.970306 | 0.024259 | 0.019667 |
| BIO12 | -0.808 | 0.140503 | 0.336584 | 0.200518 |
| BIO3 | -0.00565 | -0.07303 | -0.01214 | -0.00948 |
| BIO11 | -0.00271 | 0.009077 | -0.04348 | 0.005626 |
| BIO1 | -6.83E-05 | 0.02186 | -0.04364 | 0.003812 |
| BIO13 | -0.19188 | 0.110559 | -0.17358 | 0.265504 |
| BIO7 | 0.007683 | 0.016067 | -0.01981 | -0.0058 |
| BIO6 | -0.00417 | 0.012332 | -0.03449 | 0.009617 |
| BIO5 | 0.003508 | 0.028399 | -0.05431 | 0.003813 |
| BIO17 | -0.05348 | 0.014701 | 0.449278 | -0.0087 |
| BIO18 | -0.28915 | 0.096301 | -0.11113 | -0.92727 |
| BIO14 | -0.01427 | 0.006053 | 0.140184 | 0.010528 |
| BIO9 | 0.002269 | 0.013593 | -0.05551 | -0.00881 |
| BIO8 | 0.002903 | 0.030426 | -0.04264 | -0.0056 |
| BIO15 | -0.02156 | 0.005292 | -0.30664 | 0.085167 |
|  |  |  |  |  |
| % Variance | 83.13 | 14.45 | 1.59 | 0.73 |
|  |  |  |  |  |

**Table S8** Component loadings for the population-based PCA of the climate data. BIO1 = Annual Mean Temperature, BIO2 = Mean Diurnal Range (Mean of monthly (max temp - min temp)), BIO3 = Isothermality (BIO2/BIO7) (* 100), BIO4 = Temperature Seasonality (standard deviation *100), BIO5 = Max Temperature of Warmest Month, BIO6 = Min Temperature of Coldest Month, BIO7 = Temperature Annual Range (BIO5-BIO6), BIO8 = Mean Temperature of Wettest Quarter, BIO9 = Mean Temperature of Driest Quarter, BIO10 = Mean Temperature of Warmest Quarter, BIO11 = Mean Temperature of Coldest Quarter, BIO12 = Annual Precipitation, BIO13 = Precipitation of Wettest Month, BIO14 = Precipitation of Driest Month, BIO15 = Precipitation Seasonality (Coefficient of Variation), BIO16 = Precipitation of Wettest Quarter, BIO17 = Precipitation of Driest Quarter, BIO18 = Precipitation of Warmest Quarter, BIO19 = Precipitation of Coldest Quarter. Bolded variables are the most heavily weighted on PCs 1 and 2.

| **Climate Variable** | **PC1** | **PC2** | **PC3** | **PC4** |
| --- | --- | --- | --- | --- |
|  |  |  |  |  |
| BIO10 | 0.008175 | -0.03652 | -0.03009 | 0.032591 |
| BIO19 | -0.03886 | 0.045964 | 0.403703 | -0.24656 |
| BIO16 | -0.40634 | -0.24361 | -0.52478 | 0.076231 |
| BIO2 | 0.002593 | 0.00761 | -0.00884 | 0.033018 |
| BIO4 | 0.322829 | -0.9179 | 0.160304 | -0.09401 |
| BIO12 | -0.78284 | -0.16982 | 0.208507 | -0.29415 |
| BIO3 | -0.01324 | 0.068877 | -0.01259 | 0.051606 |
| BIO11 | 0.000342 | -0.01399 | -0.03354 | 0.035768 |
| BIO1 | 0.004534 | -0.02607 | -0.03108 | 0.035837 |
| BIO13 | -0.18057 | -0.17521 | -0.2563 | -0.20756 |
| BIO7 | 0.010306 | -0.01613 | -0.01183 | 0.030755 |
| BIO6 | -0.00111 | -0.01666 | -0.02712 | 0.020469 |
| BIO5 | 0.009192 | -0.03279 | -0.03896 | 0.051224 |
| BIO17 | -0.05529 | 0.055183 | 0.374669 | -0.27502 |
| BIO18 | -0.28176 | -0.13725 | 0.409606 | 0.818741 |
| BIO14 | -0.01592 | 0.015918 | 0.111067 | -0.09809 |
| BIO9 | 0.005448 | -0.02203 | -0.03789 | 0.05025 |
| BIO8 | 0.008618 | -0.03415 | -0.02446 | 0.0376 |
| BIO15 | -0.02121 | -0.06366 | -0.31404 | 0.155089 |
|  |  |  |  |  |
| % Variance | 87.13 | 10.94 | 1.00 | 0.66 |
|  |  |  |  |  |
