# Supplementary figures and images for: What Predicts Gene Flow During Speciation? The Relative Roles of Time, Space, Morphology and Climate
Source: Mol Ecol. 2024 Nov 7;33(23):e17580. doi: 10.1111/mec.17580 (PMC11589662; doi:10.1111/mec.17580)

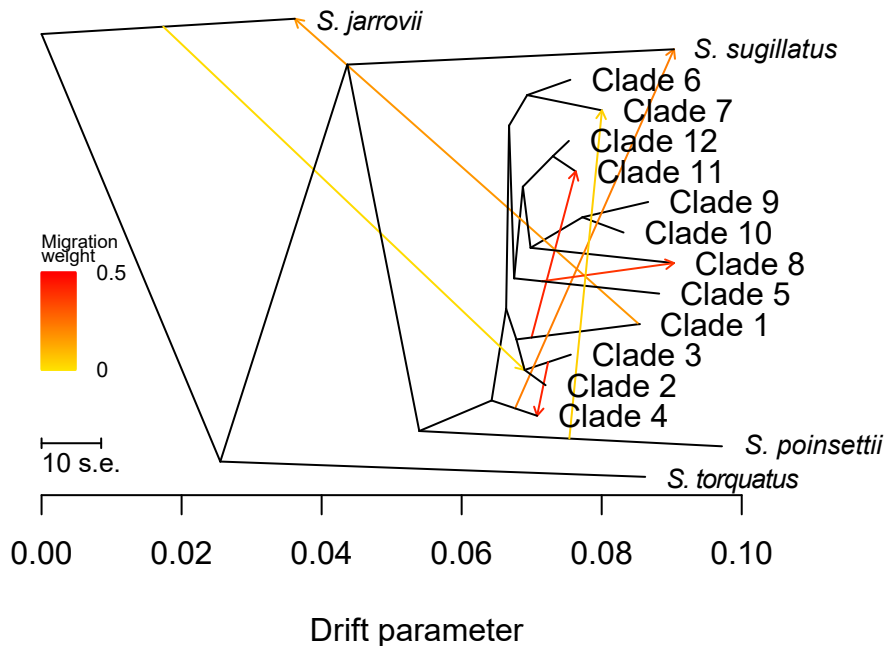

Supplement: Supplementary file 1 — Data S1. [file MEC-33-e17580-s001.zip › Supplementary_v3/FigS33.pdf]

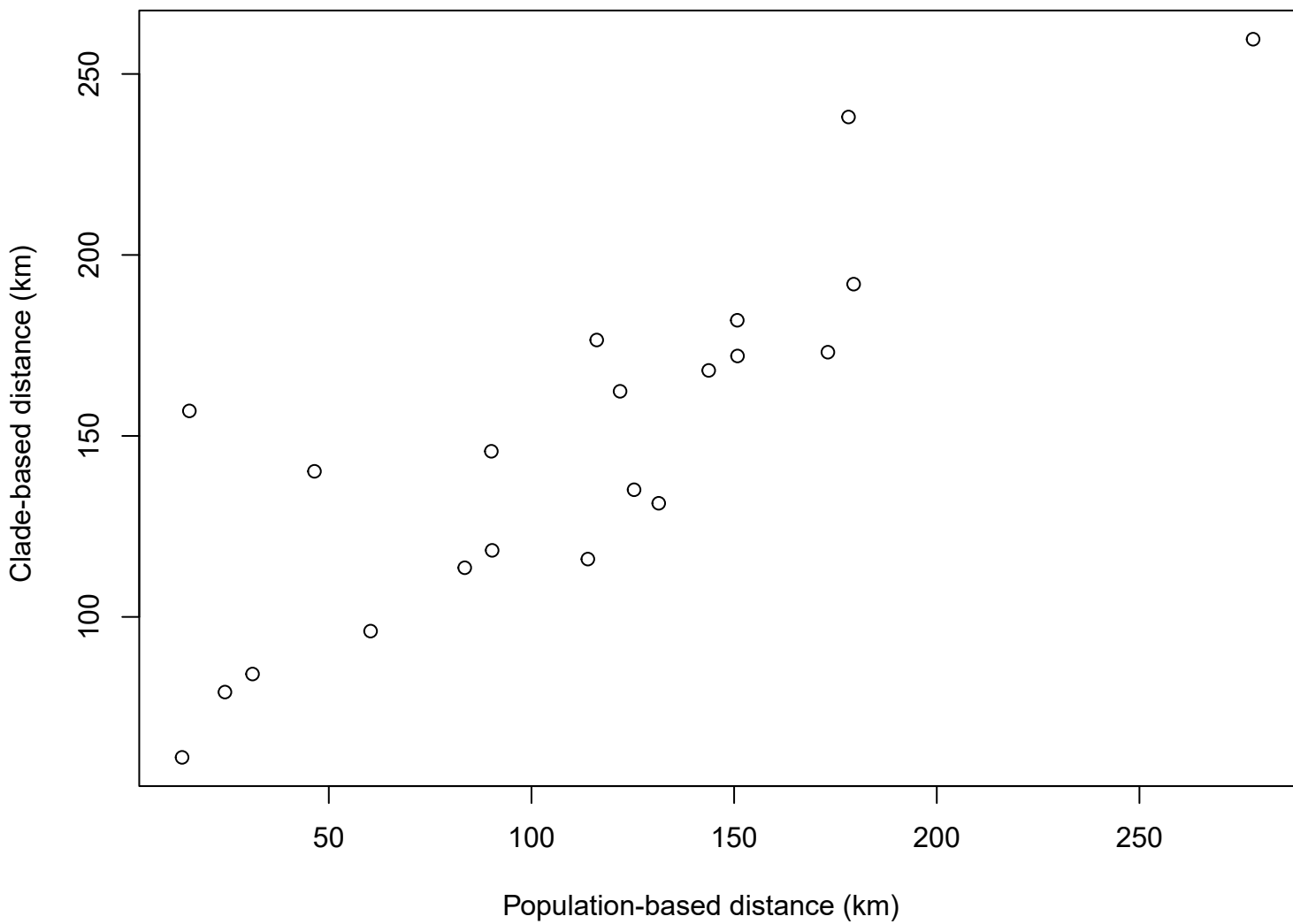

Supplement: Supplementary file 1 — Data S1. [file MEC-33-e17580-s001.zip › Supplementary_v3/Fig_S10_geo_distances.pdf]

**A**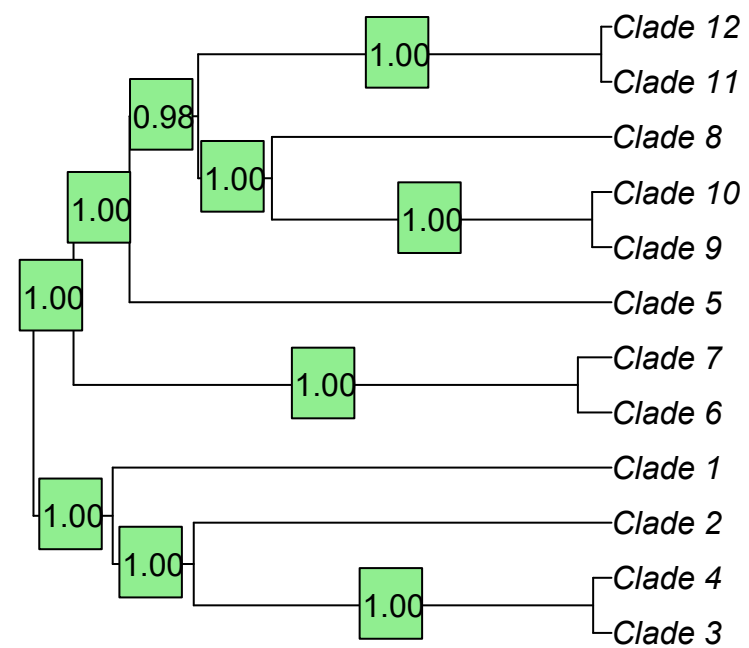**B**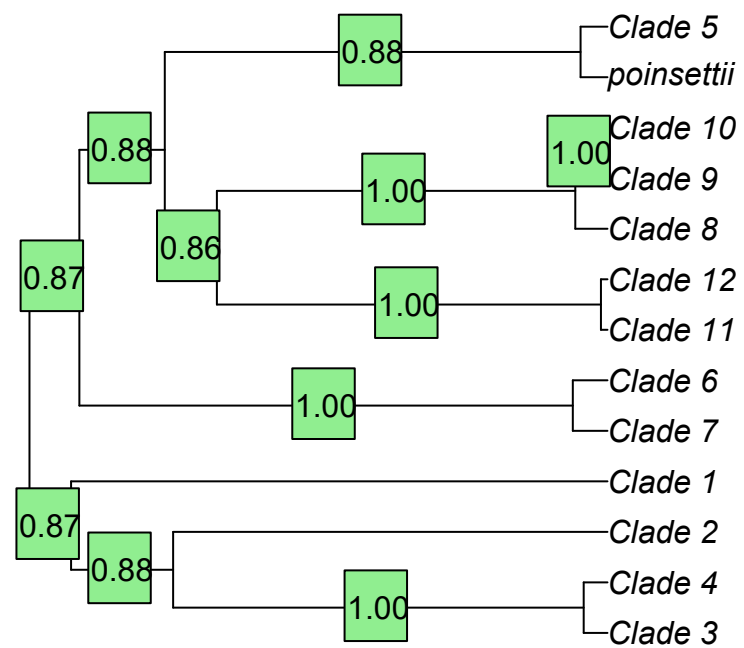**C**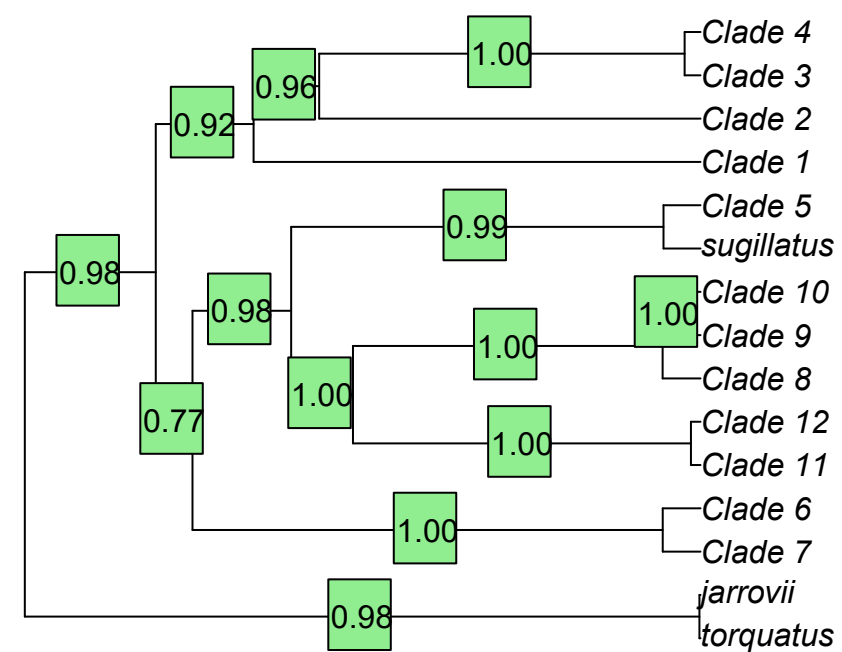**D**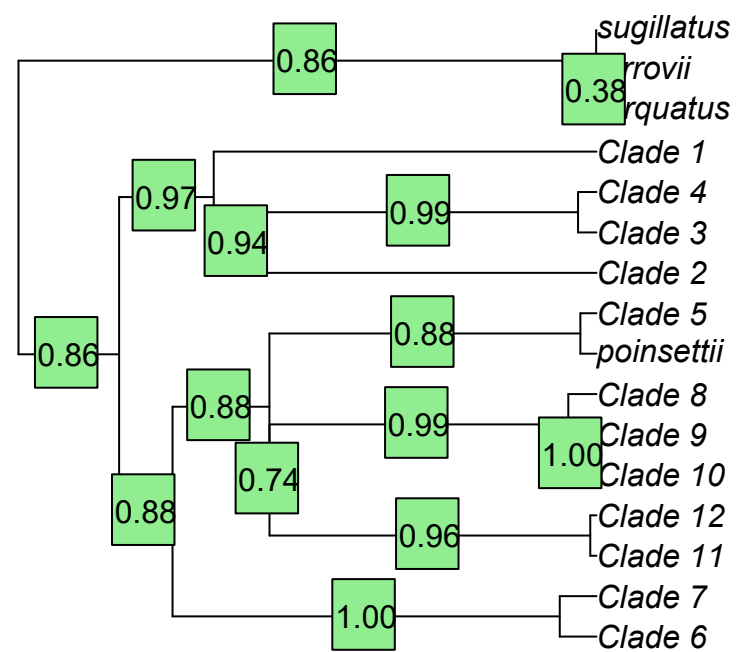**E**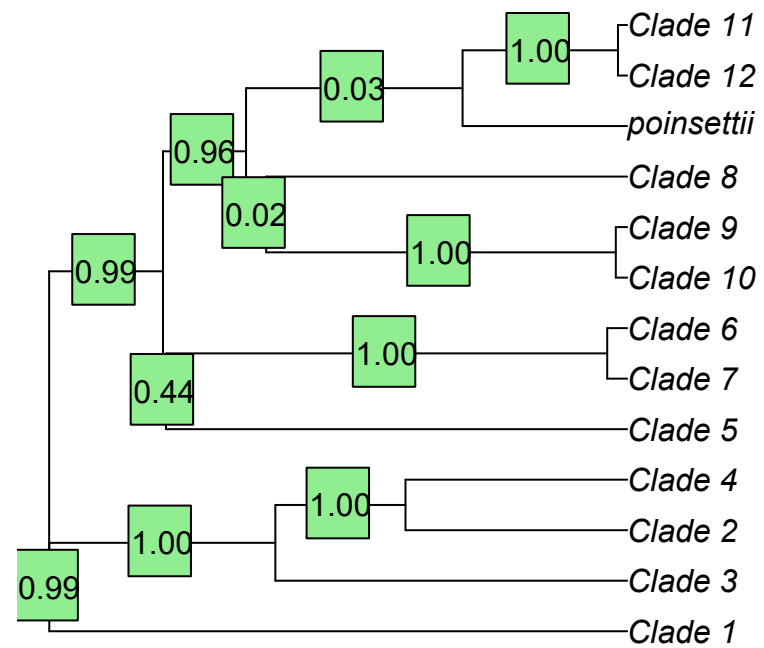

Supplement: Supplementary file 1 — Data S1. [file MEC-33-e17580-s001.zip › Supplementary_v3/Fig_S1_July_2024.pdf]

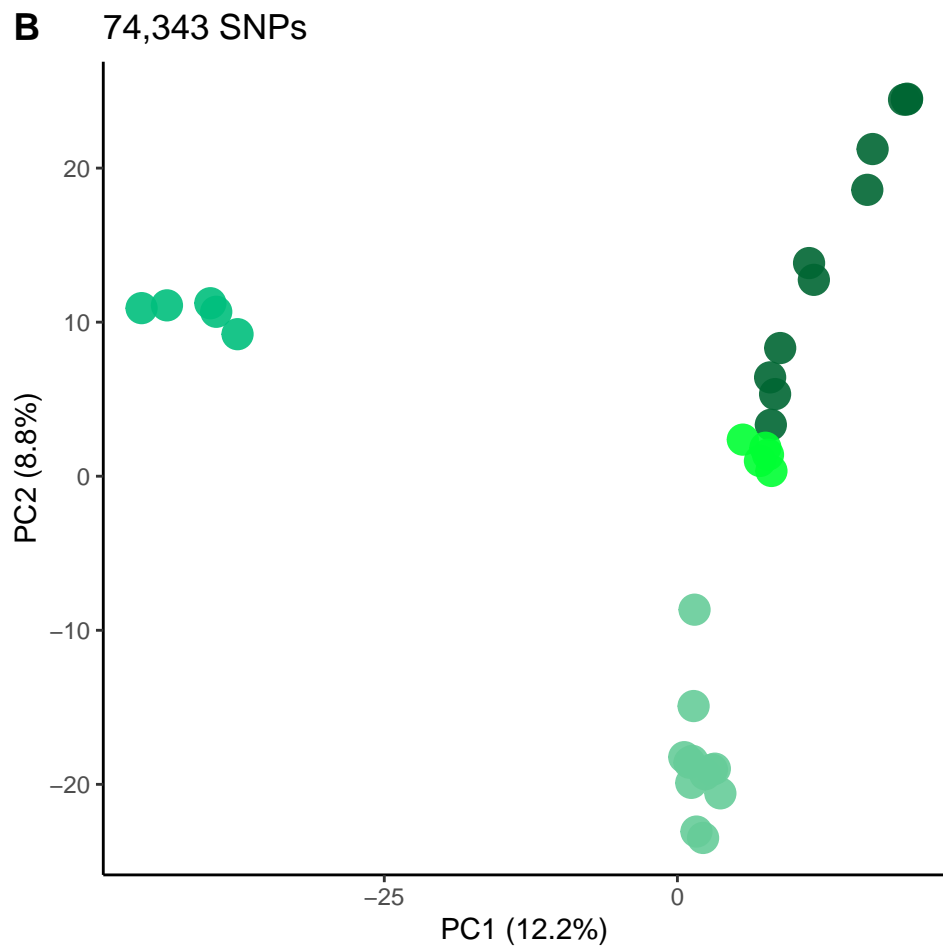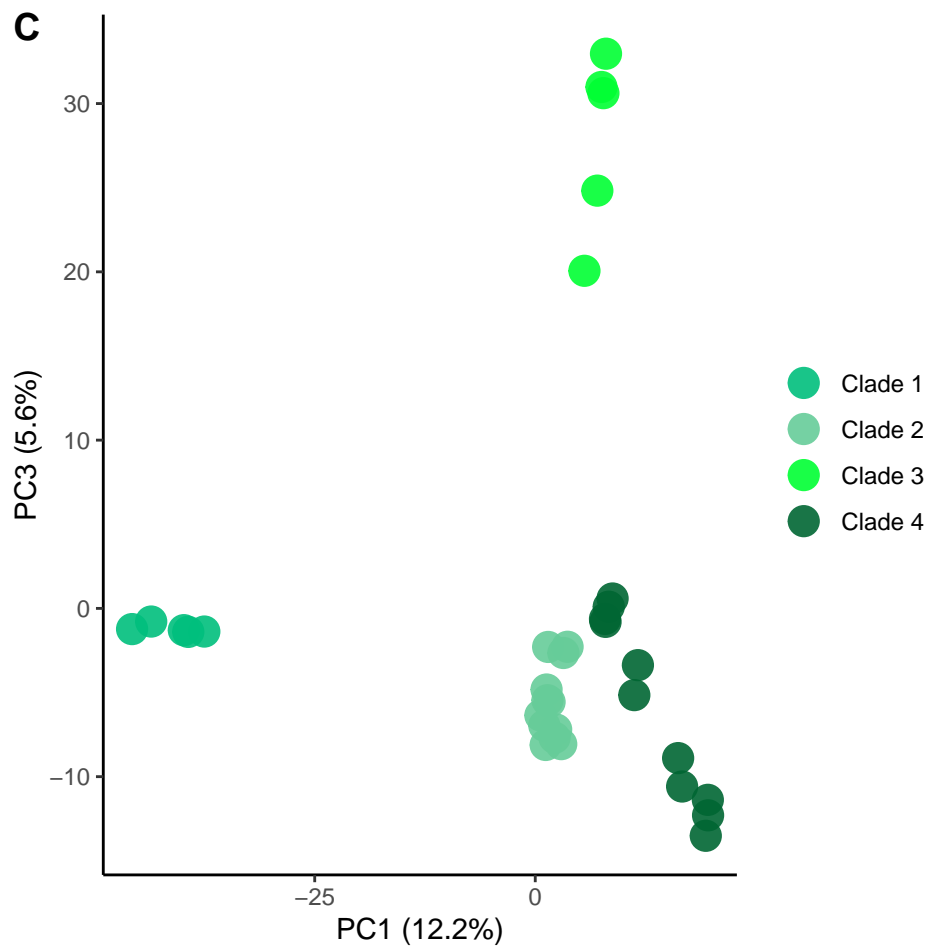

Supplement: Supplementary file 1 — Data S1. [file MEC-33-e17580-s001.zip › Supplementary_v3/Fig_S2.pdf]

**E** 74,305 SNPs

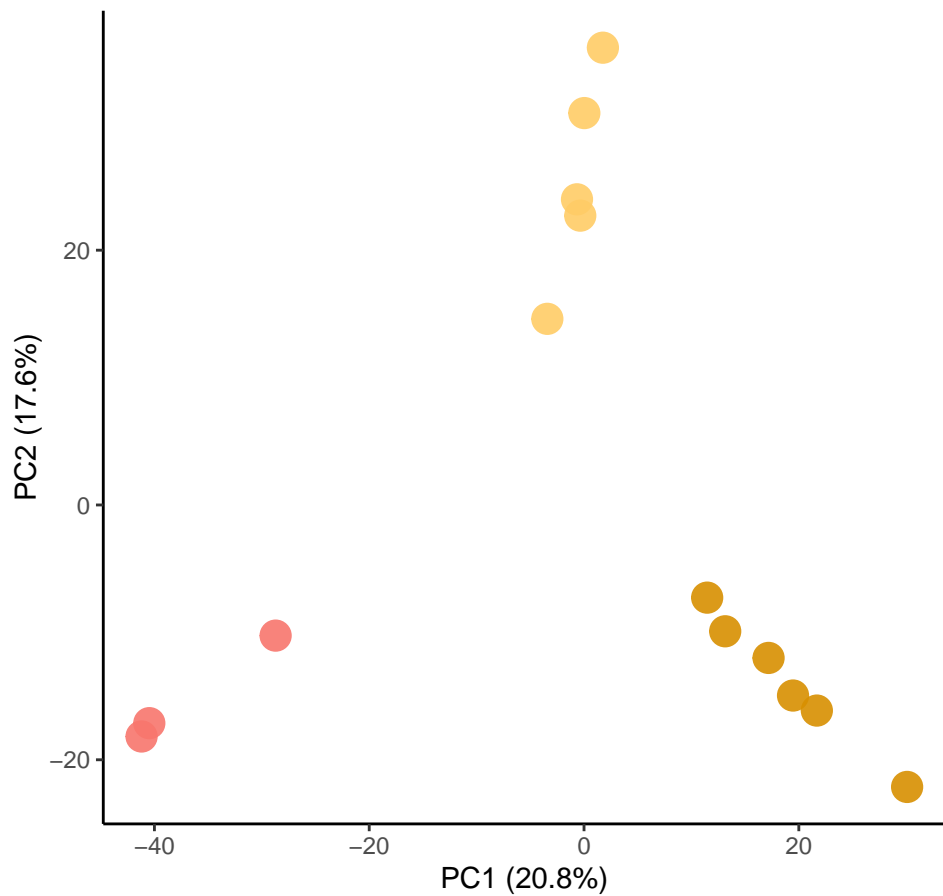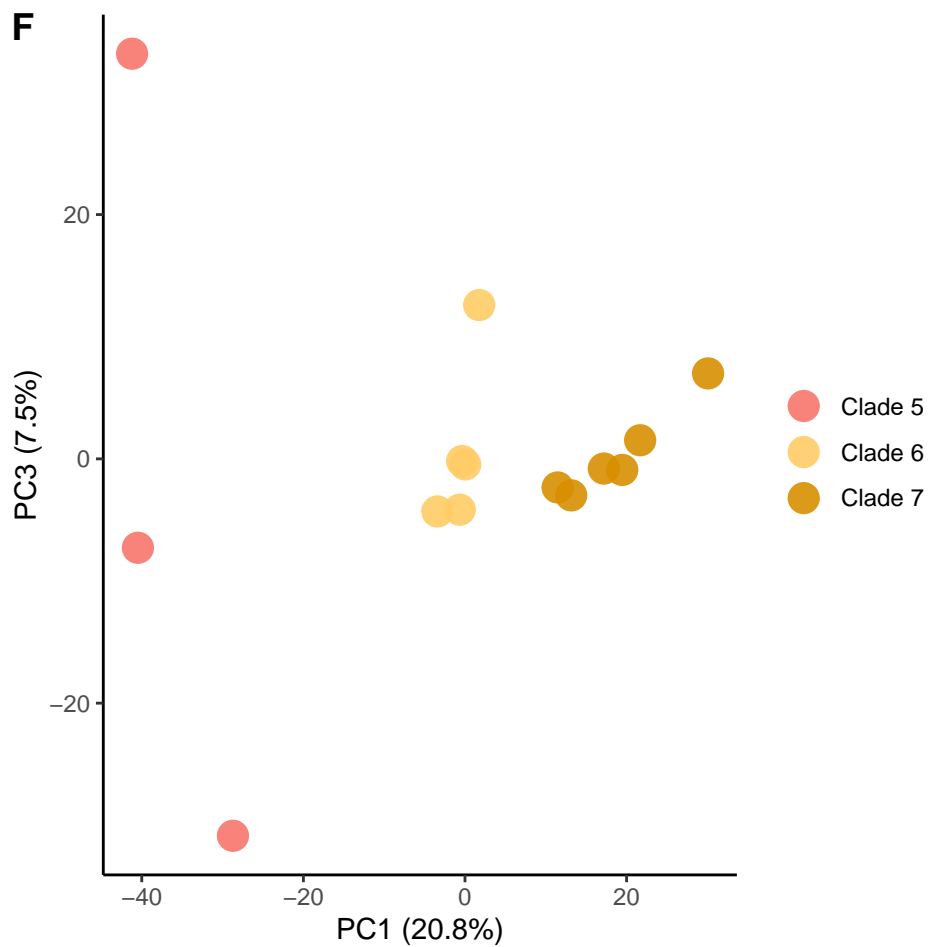

Supplement: Supplementary file 1 — Data S1. [file MEC-33-e17580-s001.zip › Supplementary_v3/Fig_S3.pdf]

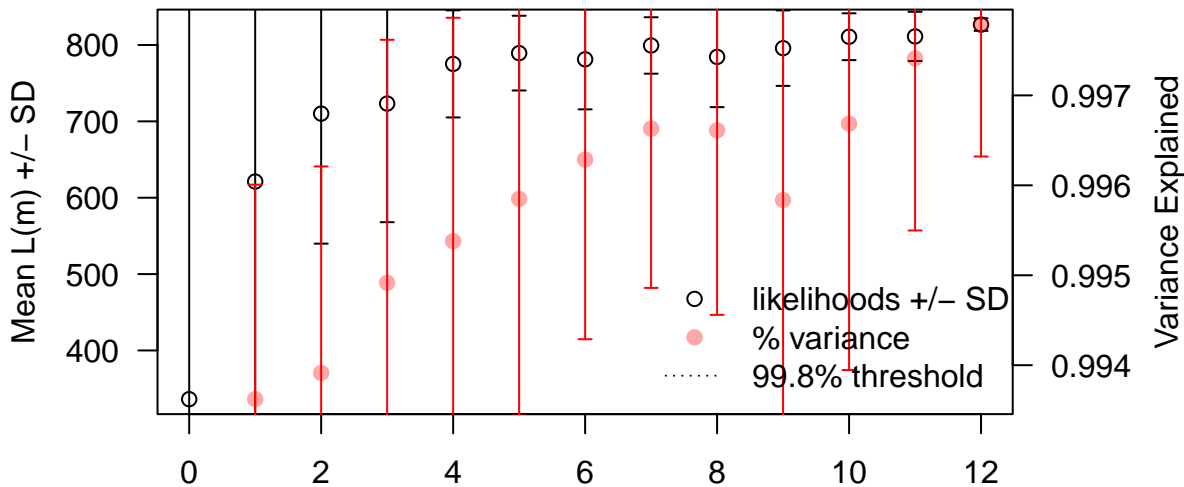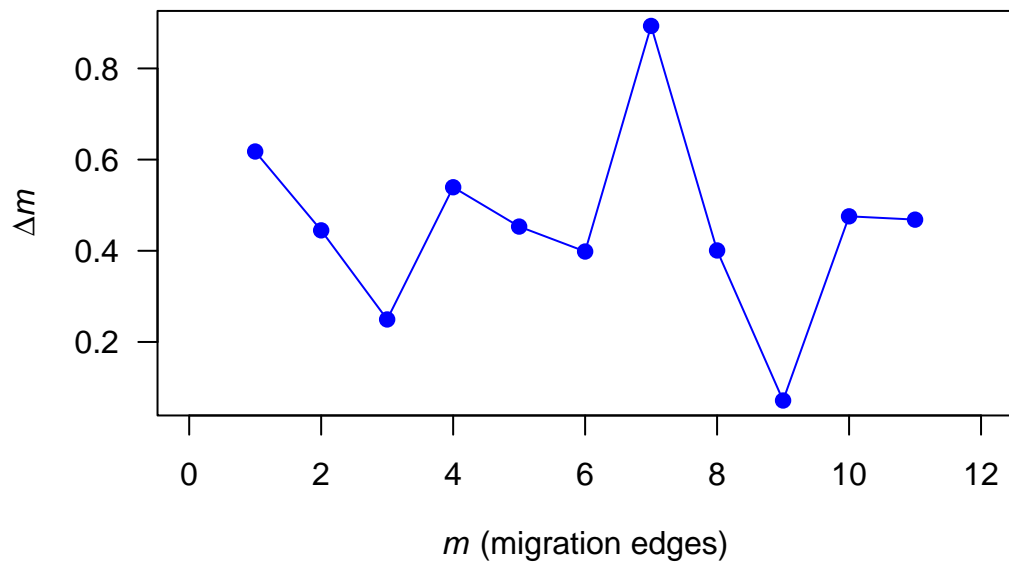

Supplement: Supplementary file 1 — Data S1. [file MEC-33-e17580-s001.zip › Supplementary_v3/Fig_S32_OptM.pdf]

Population-based morphological divergence

8  
6  
4  
2  
0

0

5

10

15

Clade-based morphological divergence

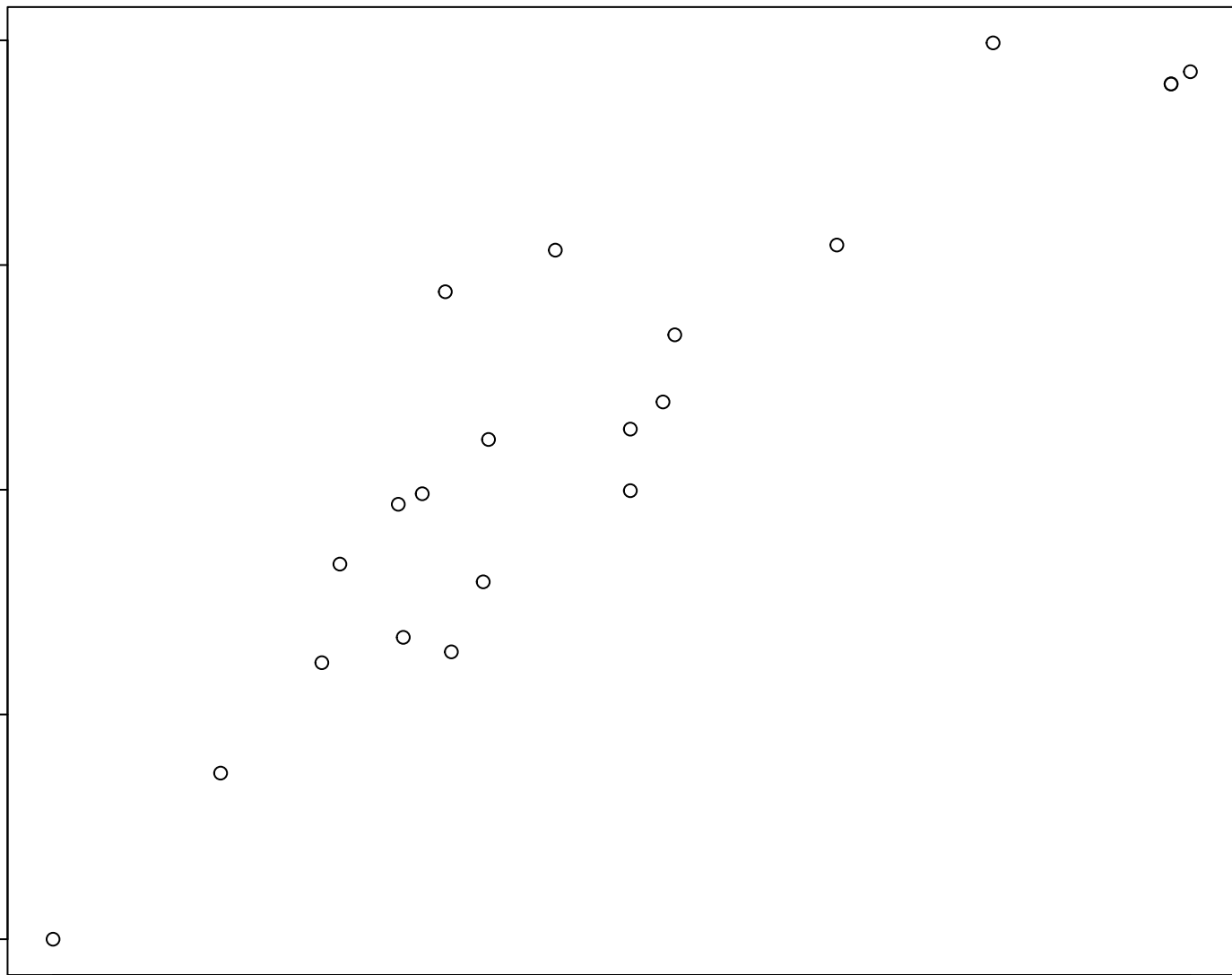

Supplement: Supplementary file 1 — Data S1. [file MEC-33-e17580-s001.zip › Supplementary_v3/Fig_S34_Population_Clade_morphology.pdf]

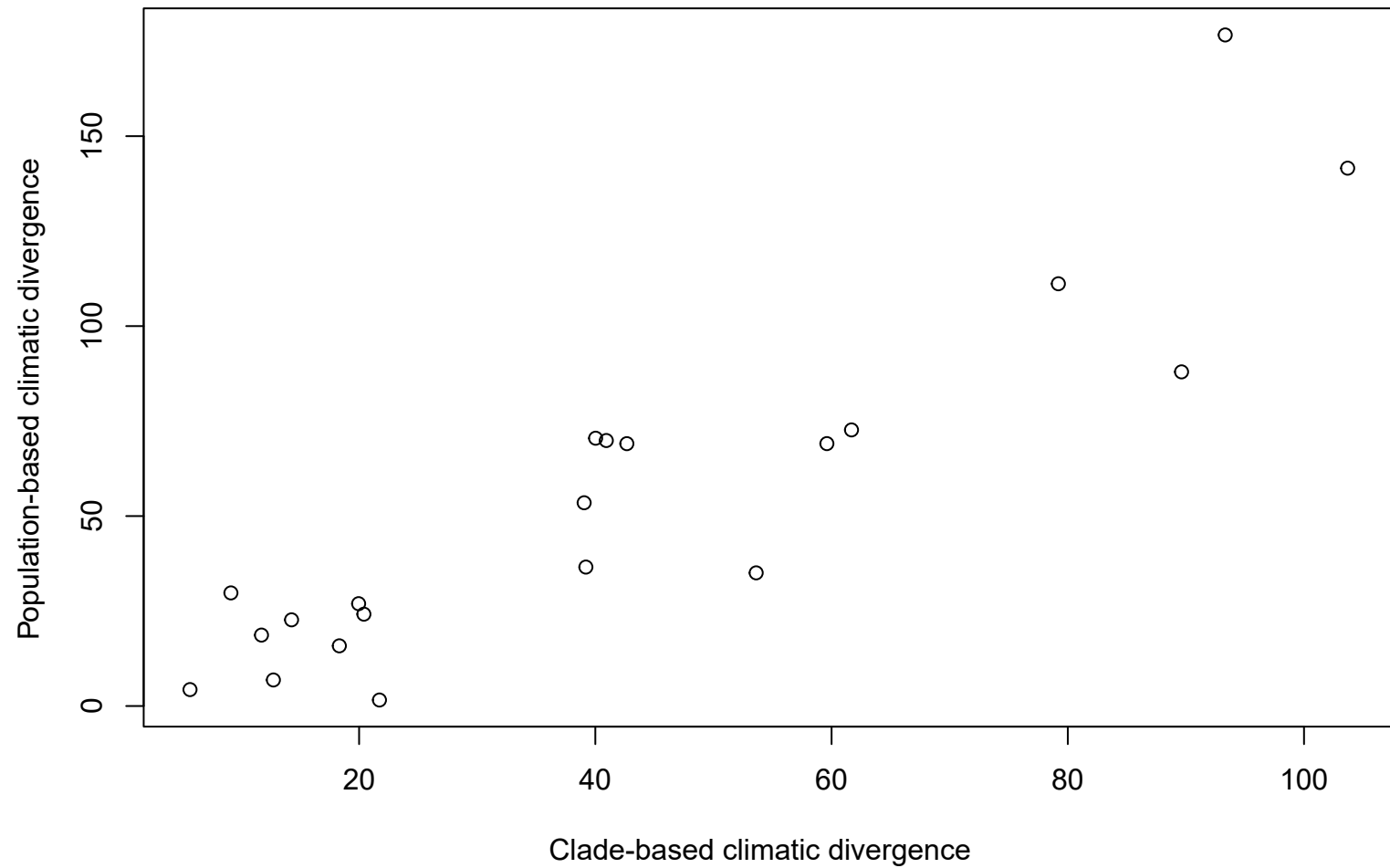

Supplement: Supplementary file 1 — Data S1. [file MEC-33-e17580-s001.zip › Supplementary_v3/Fig_S35_Population_Clade_climate.pdf]

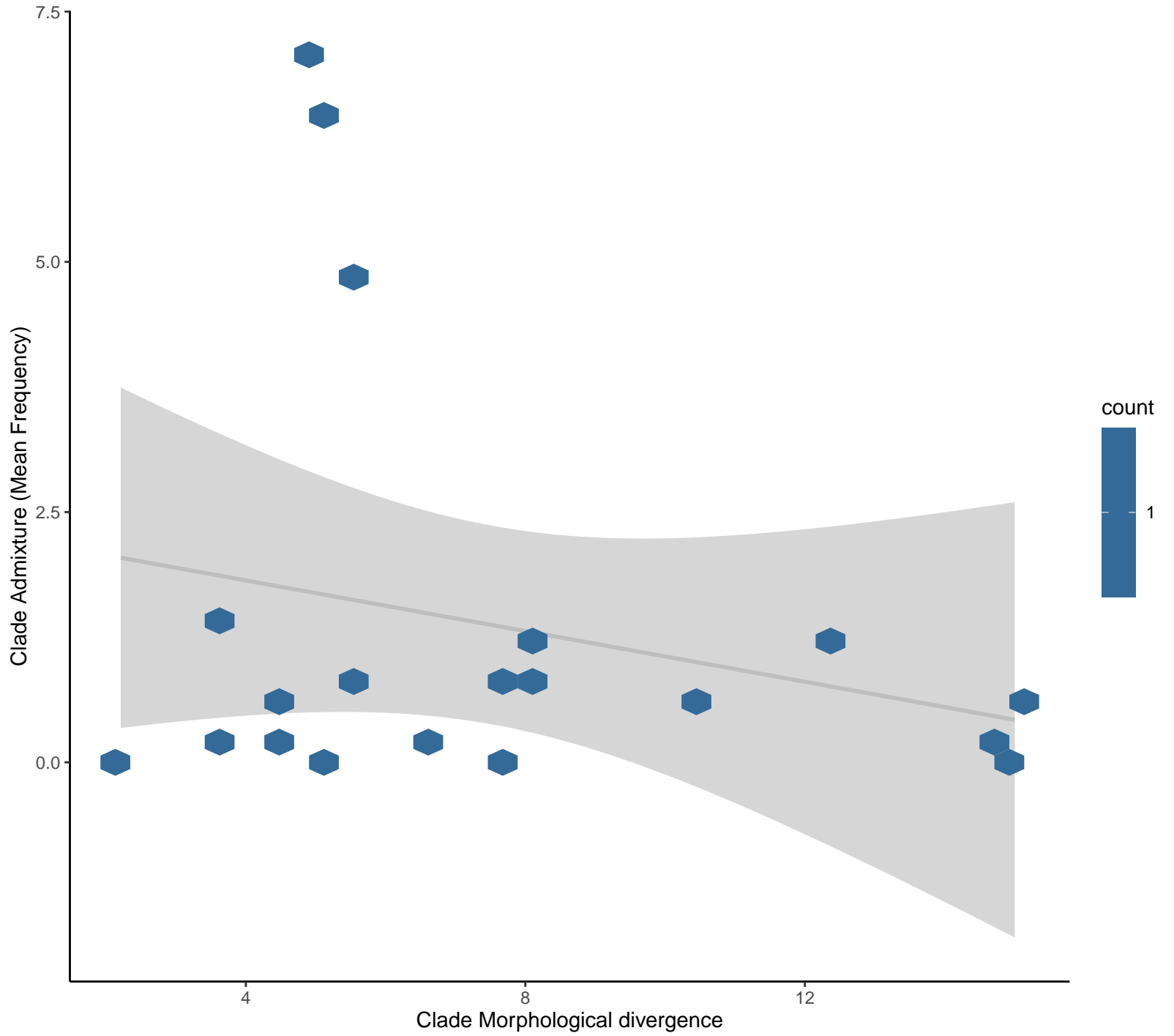

Supplement: Supplementary file 1 — Data S1. [file MEC-33-e17580-s001.zip › Supplementary_v3/Fig_S36_July_2024.pdf]

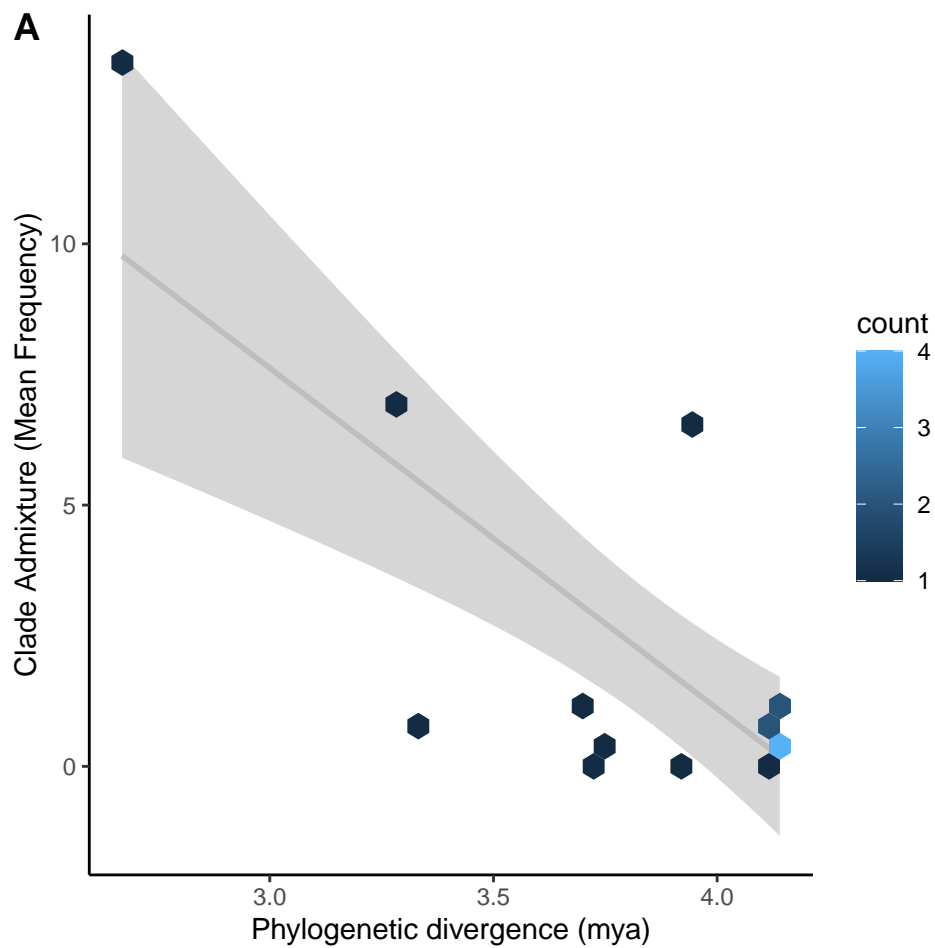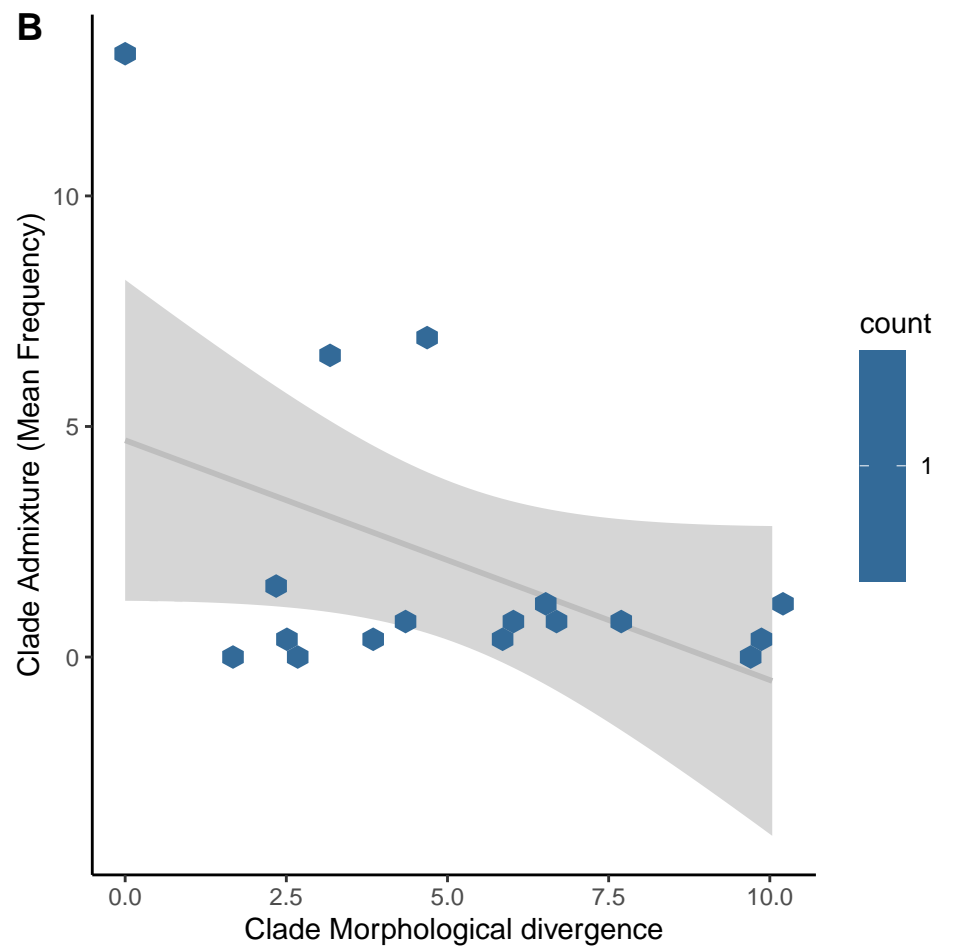

Supplement: Supplementary file 1 — Data S1. [file MEC-33-e17580-s001.zip › Supplementary_v3/Fig_S37_nocyanos.pdf]

H

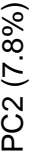

- Clade 8
- Clade 9
- Clade 10

Supplement: Supplementary file 1 — Data S1. [file MEC-33-e17580-s001.zip › Supplementary_v3/Fig_S4.pdf]

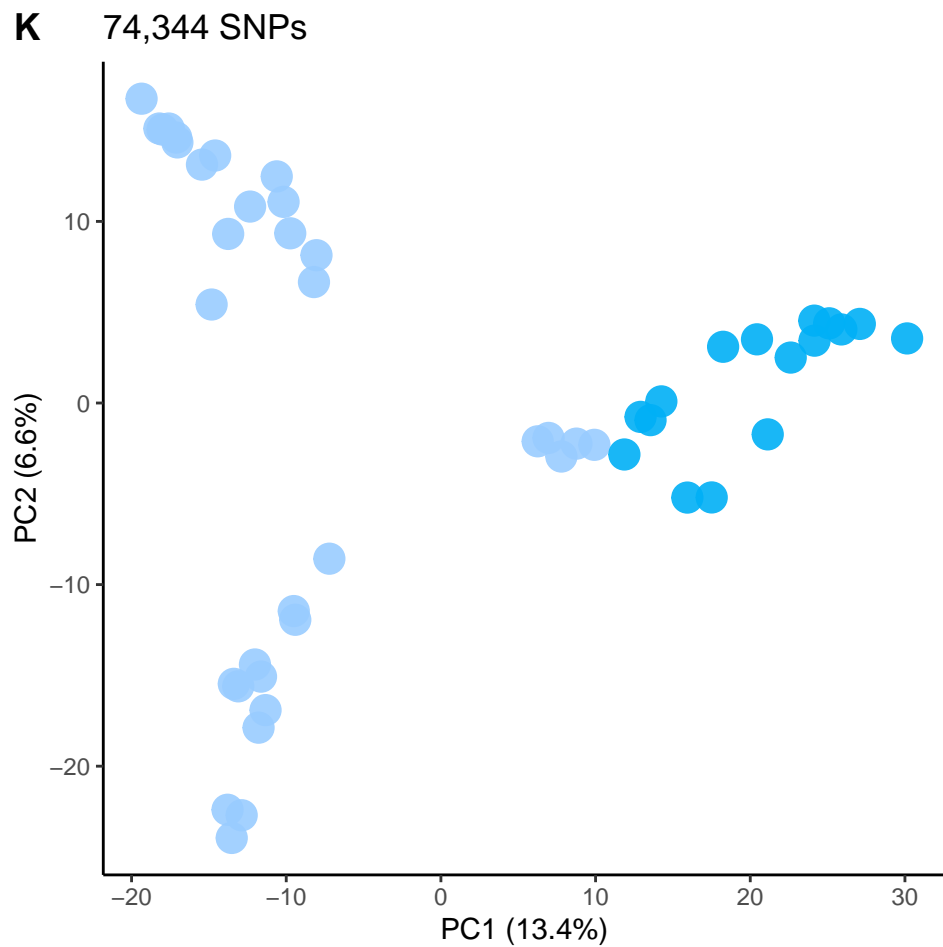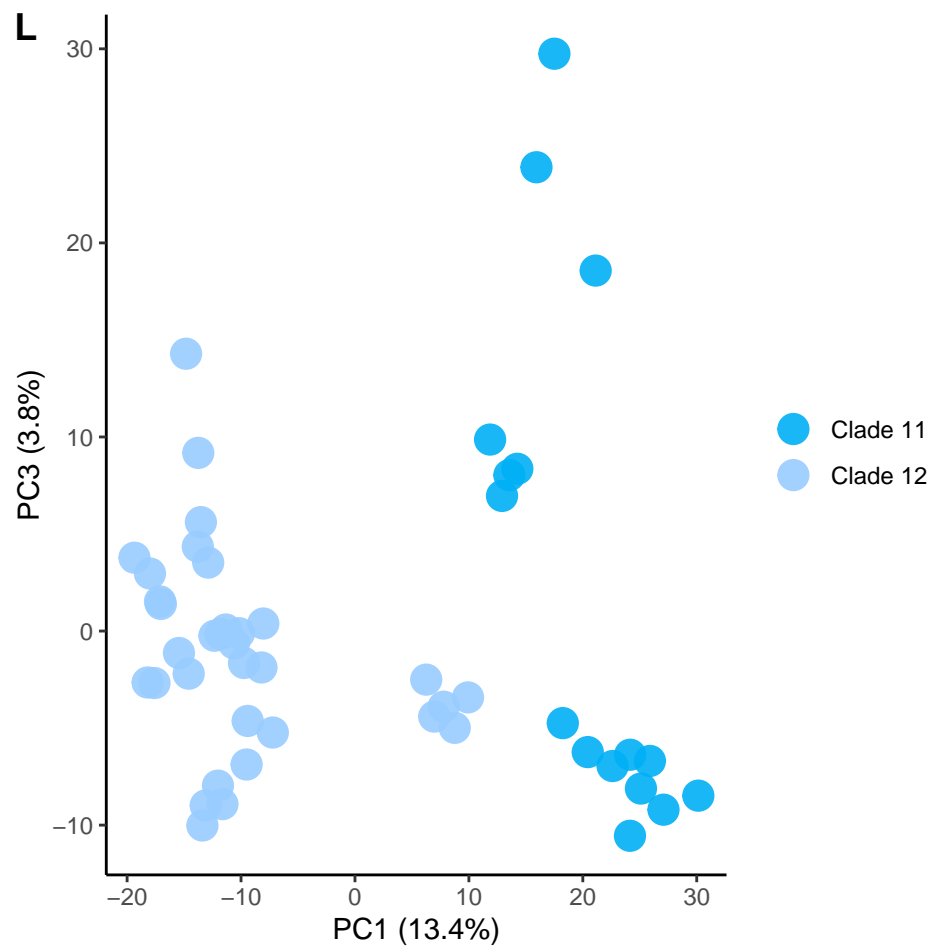

Supplement: Supplementary file 1 — Data S1. [file MEC-33-e17580-s001.zip › Supplementary_v3/Fig_S5.pdf]

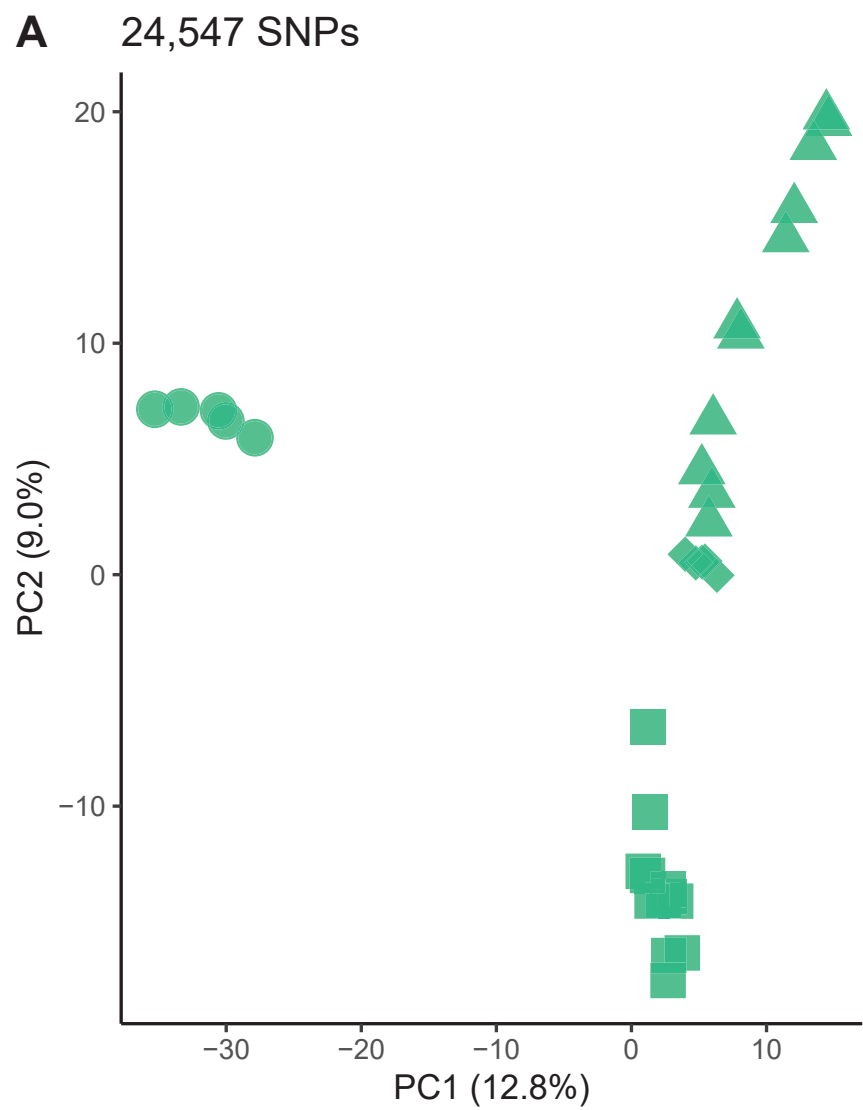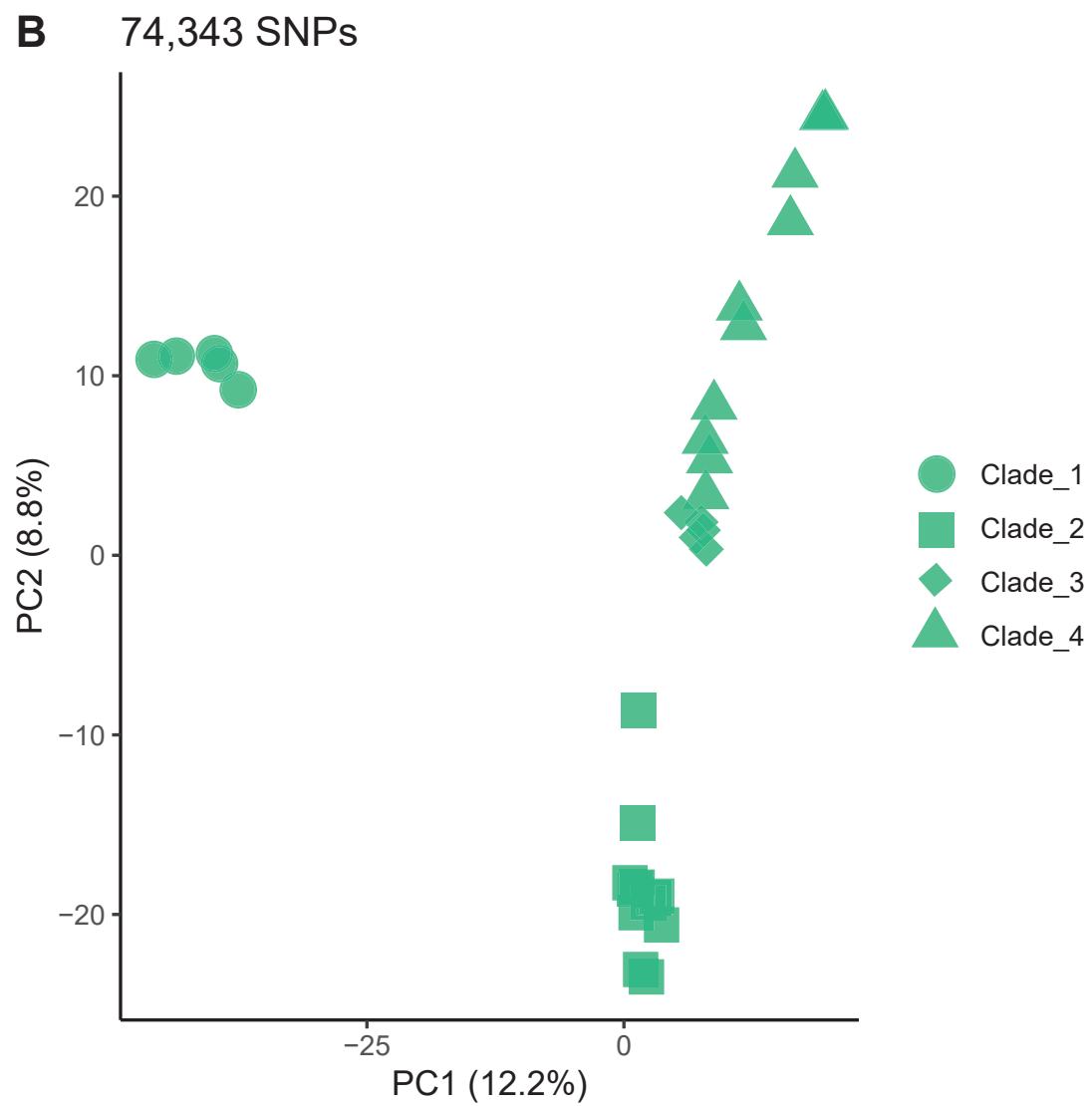

Supplement: Supplementary file 1 — Data S1. [file MEC-33-e17580-s001.zip › Supplementary_v3/Fig_S6_v2.pdf]

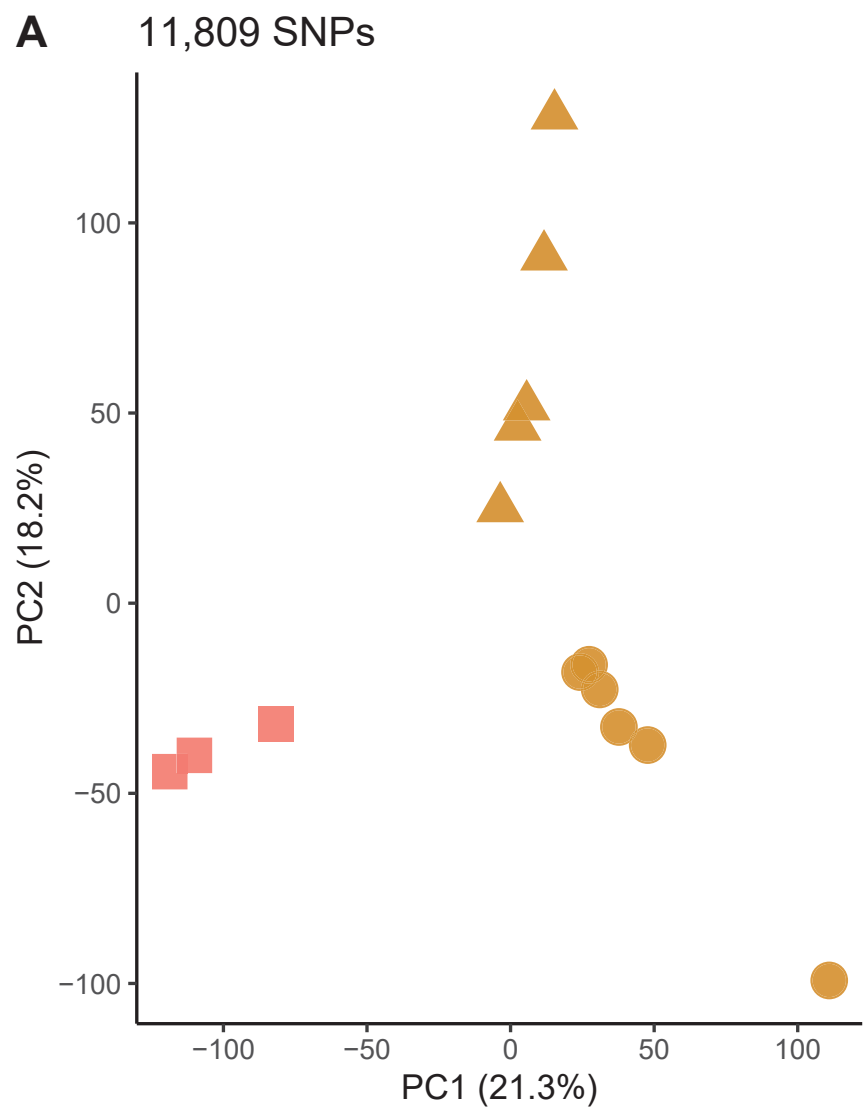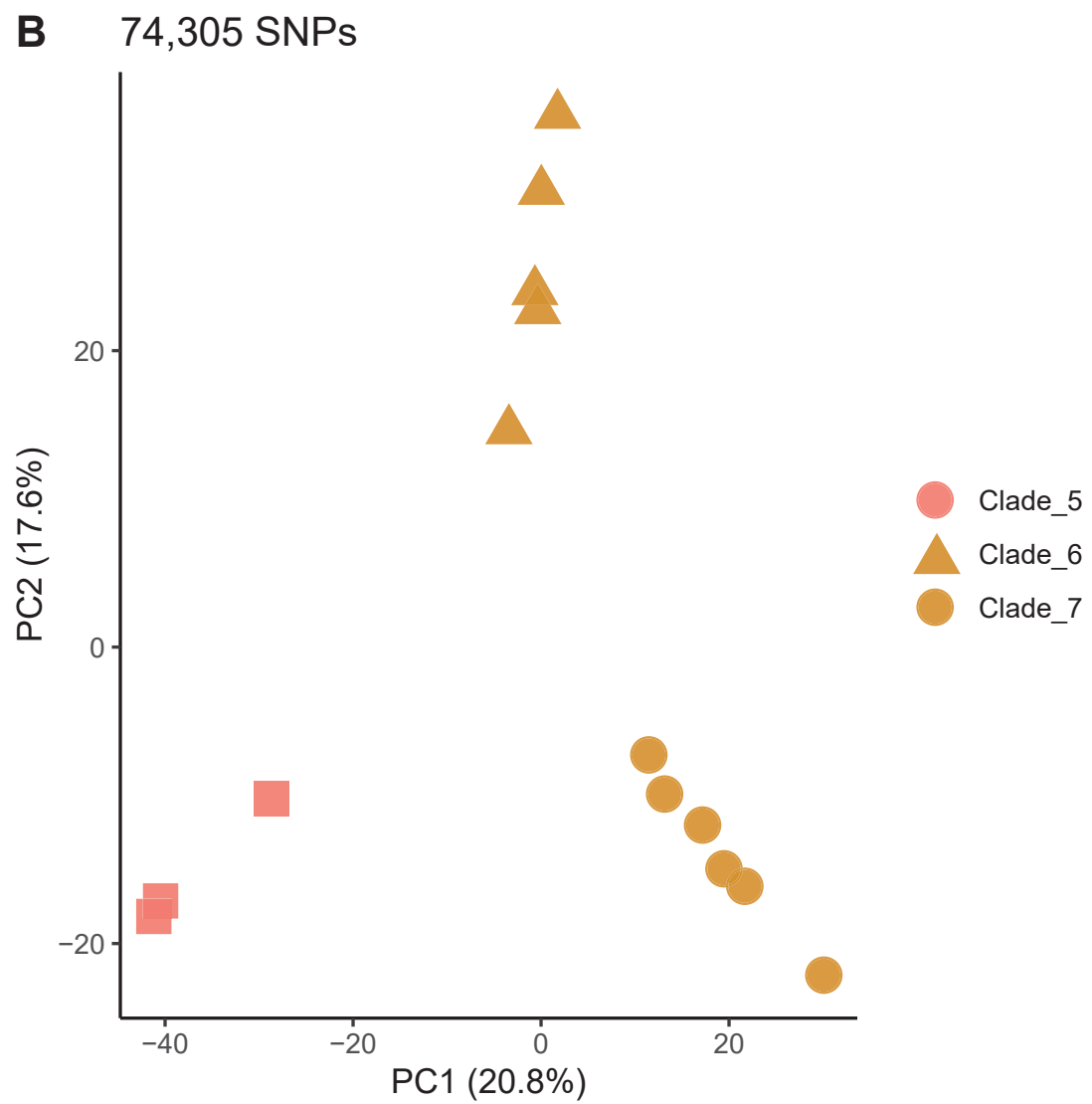

Supplement: Supplementary file 1 — Data S1. [file MEC-33-e17580-s001.zip › Supplementary_v3/Fig_S7_v2.pdf]

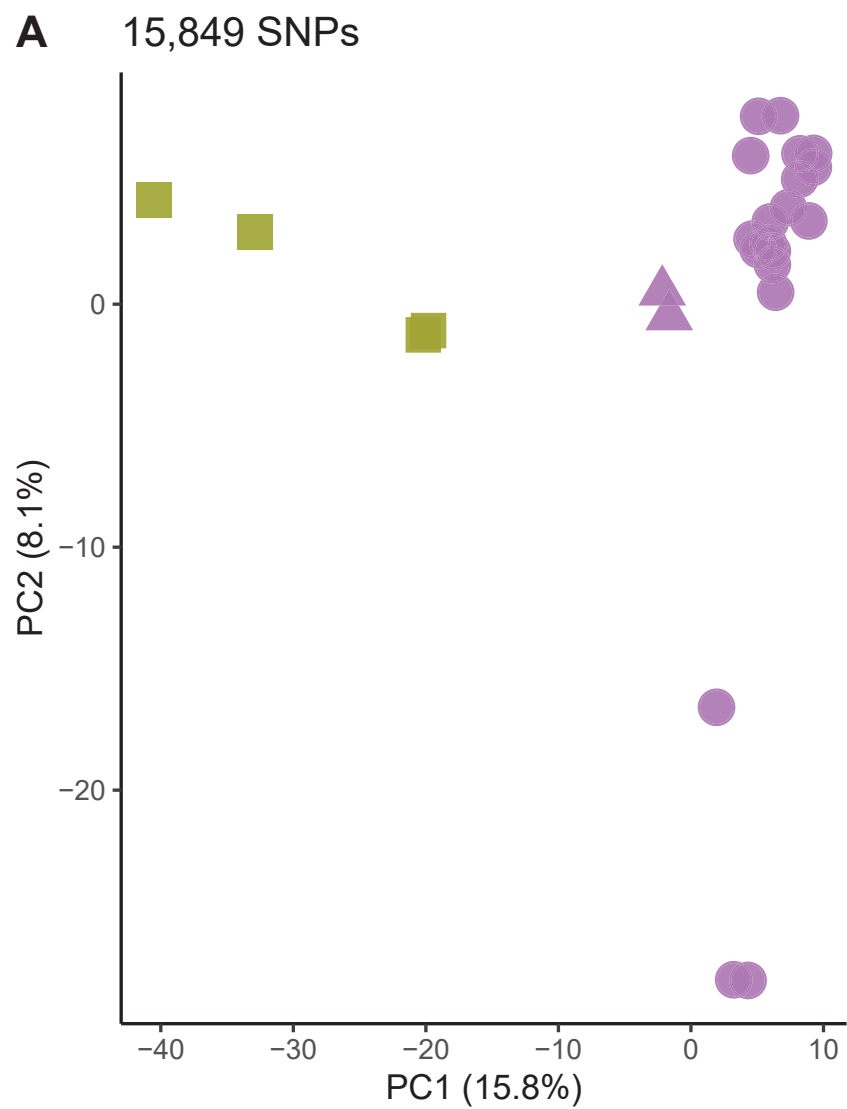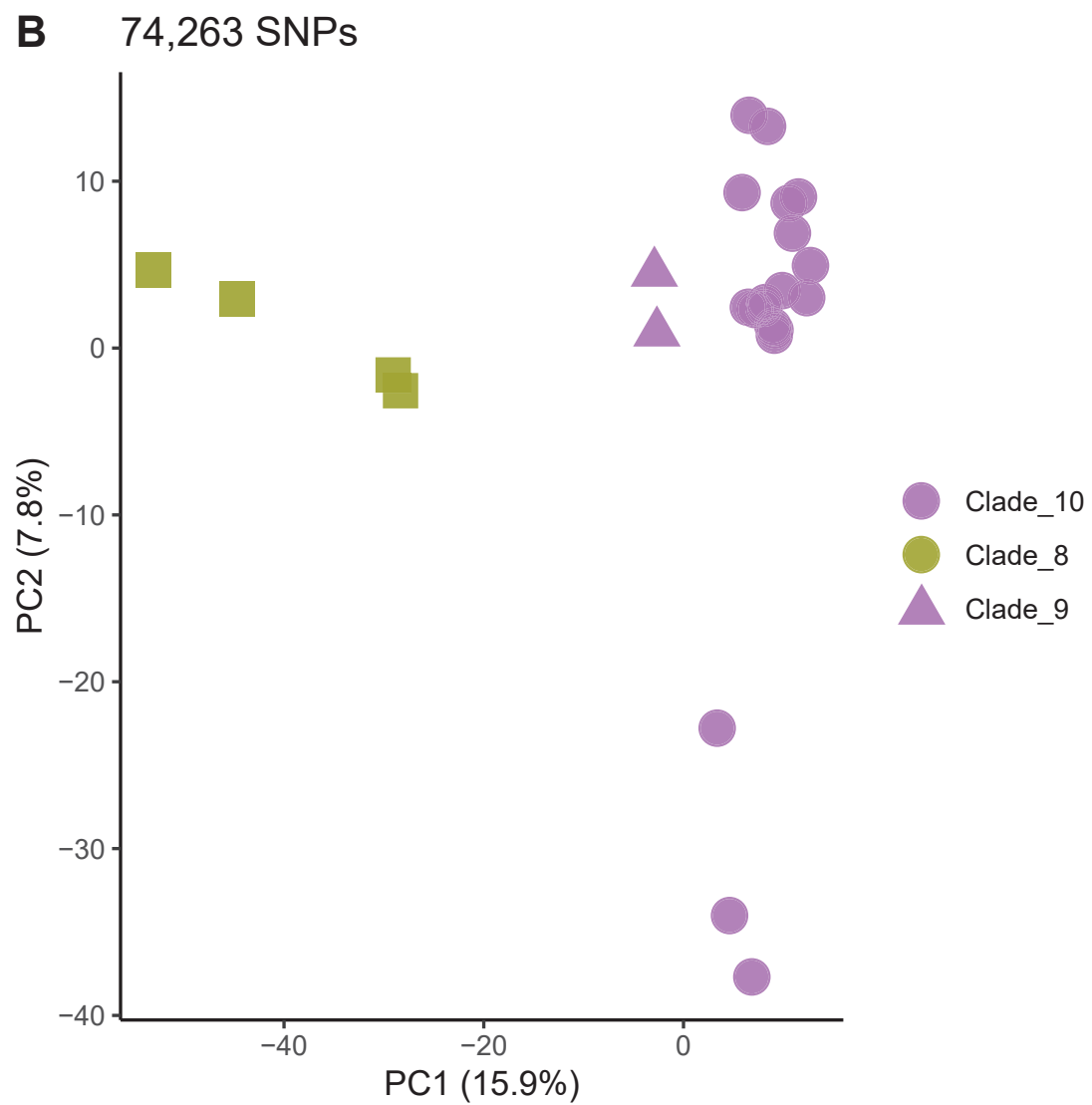

Supplement: Supplementary file 1 — Data S1. [file MEC-33-e17580-s001.zip › Supplementary_v3/Fig_S8_v2.pdf]

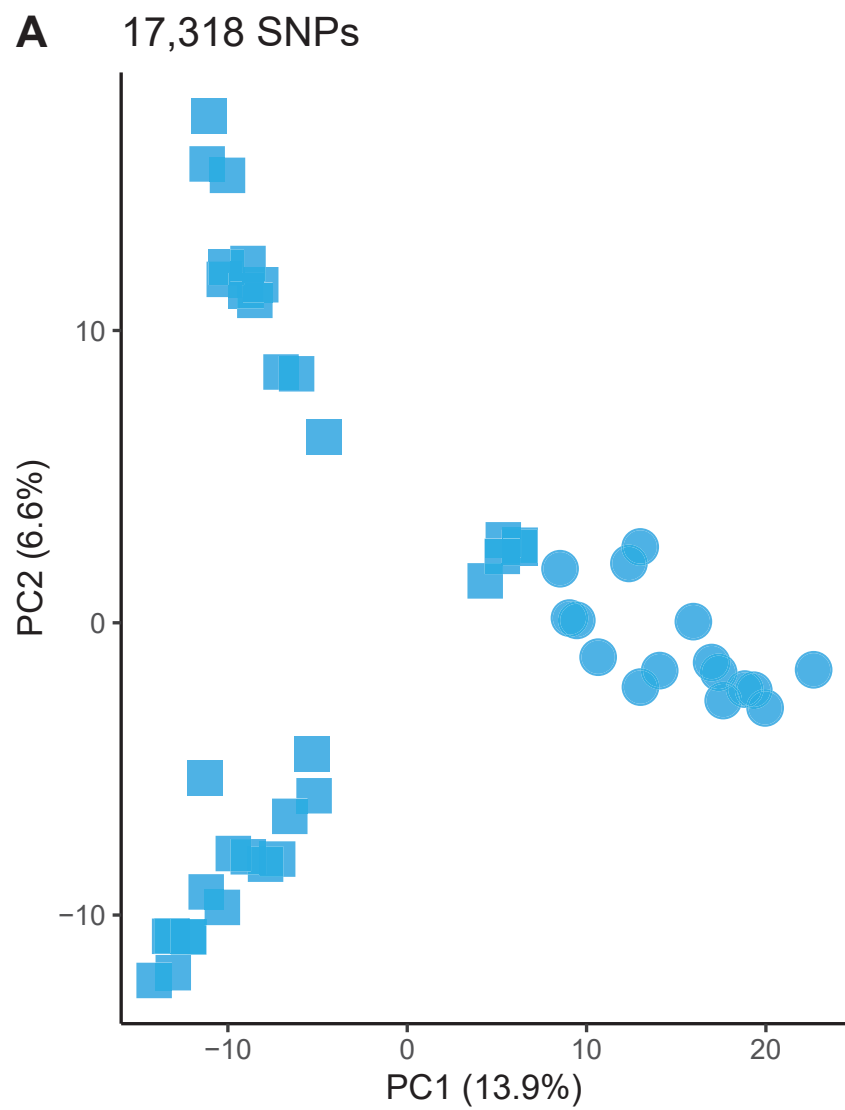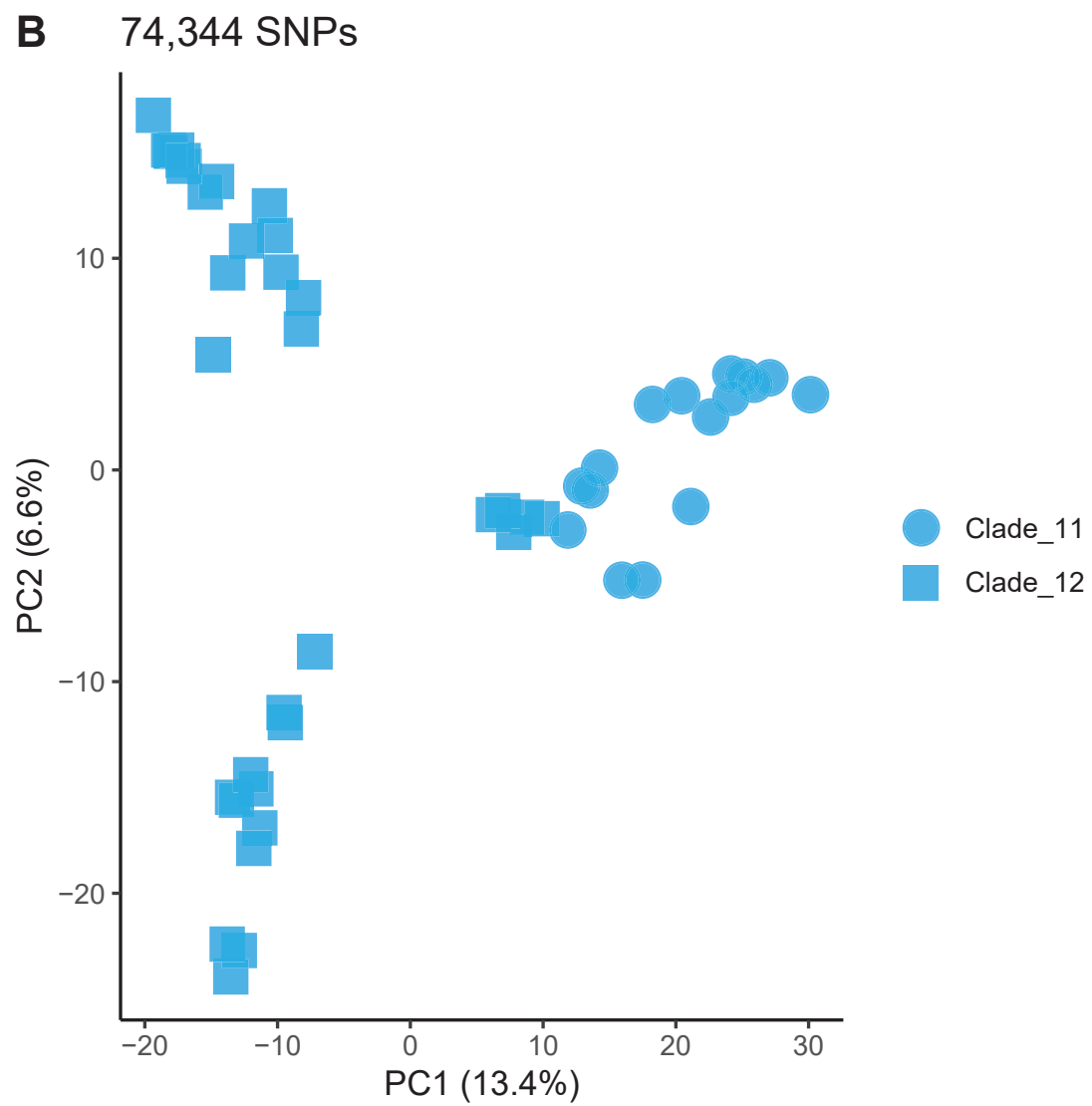

Supplement: Supplementary file 1 — Data S1. [file MEC-33-e17580-s001.zip › Supplementary_v3/Fig_S9_v2.pdf]
